# Unusual peptide-binding proteins guide pyrroloindoline alkaloid formation in crocagin biosynthesis

**DOI:** 10.1038/s41557-023-01153-w

**Published:** 2023-03-09

**Authors:** Sebastian Adam, Dazhong Zheng, Andreas Klein, Carsten Volz, William Mullen, Sally L. Shirran, Brian O. Smith, Olga V. Kalinina, Rolf Müller, Jesko Koehnke

**Affiliations:** 1 Workgroup Structural Biology of Biosynthetic Enzymes, Helmholtz Institute for Pharmaceutical Research Saarland (HIPS), Helmholtz Centre for Infection Research (HZI), Saarland University, Saarbrücken, Germany; 2 School of Chemistry, University of Glasgow, Glasgow, UK; 3 Department of Microbial Natural Products, HIPS; HZI; Department of Pharmacy, Saarland University, Saarbrücken, Germany; 4 Institute of Cardiovascular and Medical Sciences, University of Glasgow, Glasgow, UK; 5 Biomedical Sciences Research Complex, University of St Andrews, St Andrews, UK; 6 School of Molecular Biosciences, University of Glasgow, Glasgow, UK; 7 Drug Bioinformatics Group, HIPS, HZI, Saarland University, Saarbrücken, Germany; 8 Medical Faculty, Saarland University, Homburg, Germany; 9 Center for Bioinformatics, Saarbrücken, Germany; 10 Hannover-Braunschweig Site, German Centre for Infection Research (DZIF), Hanover, Germany

**Keywords:** Peptides, X-ray crystallography

## Abstract

Ribosomally synthesized and post-translationally modified peptide natural products have provided many highly unusual scaffolds. This includes the intriguing alkaloids crocagins, which possess a tetracyclic core structure and whose biosynthesis has remained enigmatic. Here we use in vitro experiments to demonstrate that three proteins, CgnB, CgnC and CgnE, are sufficient for the production of the hallmark tetracyclic crocagin core from the precursor peptide CgnA. The crystal structures of the homologues CgnB and CgnE reveal them to be the founding members of a peptide-binding protein family and allow us to rationalize their distinct functions. We further show that the hydrolase CgnD liberates the crocagin core scaffold, which is subsequently *N*-methylated by CgnL. These insights allow us to propose a biosynthetic scheme for crocagins. Bioinformatic analyses based on these data led to the discovery of related biosynthetic pathways that may provide access to a structurally diverse family of peptide-derived pyrroloindoline alkaloids.

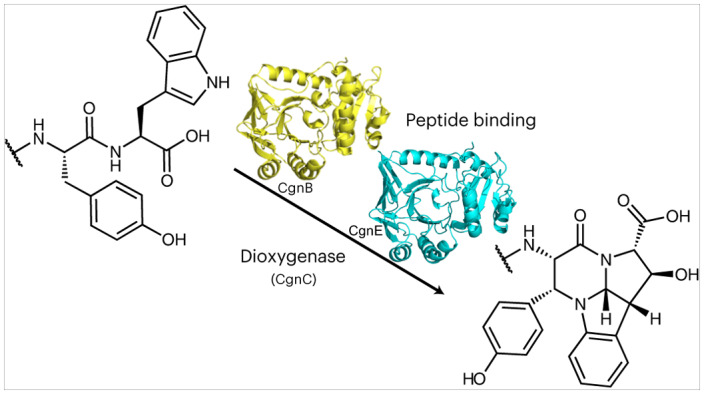

## Abstract (web summary)

The alkaloids crocagins are derived from a ribosomal peptide through a series of enzymatic post-translational modifications. A combination of biochemistry and structural biology techniques has now been used to elucidate this biosynthetic pathway, propose a mechanism for the formation of the tetracyclic core structure and enable genome mining for related natural products.

## Main

**Fig. 1:**
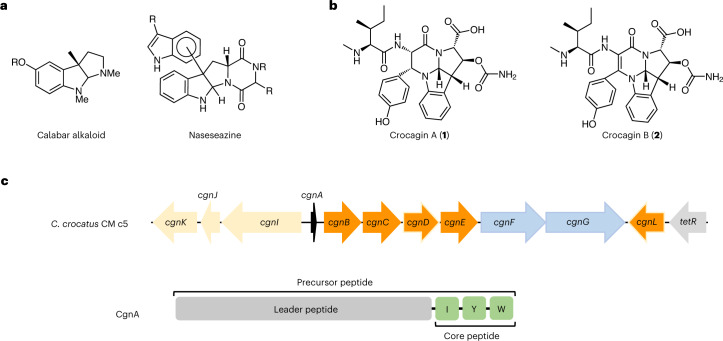
Natural products containing pyrroloindoline moieties and the crocagin BGC. **a**, Structures of the calabar alkaloid (physostigmine) and naseseazine. **b**, Chemical structures of crocagins A (**1**) and B (**2**). **c**, Crocagin BGC found in Chondromyces crocatus CM c5. Genes encoding for putative biosynthetic proteins are shown in orange (dark orange is part of this study); predicted transport proteins, in blue; a regulatory protein, in grey; and the precursor peptide CgnA, in black, which is depicted below the BGC. CgnA is 21 amino acids long and consists of a leader peptide (grey) and a three-amino-acid core peptide (green). Relative sizes of genes and intergenic regions are approximate.

Natural products and their derivatives have been vital for modern medicine and a main contributor to the increase in our quality of life over the past century^1,2^. Alkaloids are a profoundly important class of natural products that includes prominent compounds such as morphine and strychnine. A common motif found in alkaloids is the hexahydropyrrolo[2,3-*b*]indole, also referred to as pyrroloindoline. Pyrroloindoline-containing natural products (Fig. 1a) possess diverse and potent bioactivities, but have presented formidable synthetic challenges^3–5^. A better understanding of their biosynthesis may advance synthetic efforts or provide semi-synthetic routes to access these valuable molecules. Pyrroloindolines are biosynthetically derived from tryptophan, and different enzymatic routes have been reported. They all result in bond formation between the tryptophan’s α-amino group and its indoles’ C2/δ-carbon: in dibrevianamide^6^, naseseazine^6^ and (−)-ditryptophenaline biosynthesis^7^, P450 enzymes create indole radicals (Supplementary Fig. 1).

In the case of okaramine^8^ or himastatin^9^, a flavin-dependent monooxygenase or P450 enzyme, respectively, has been proposed to catalyse the 2,3-epoxidation of the indole, which, after ring-opening of the epoxide, leads to a hydroxylated indole C3 and the formation of the pyrroloindoline (Supplementary Fig. 1). Finally, enzymatic methylation (cyclic ditryptophan^10^, physostigmine^11^), prenylation (ardeemin^12^, acetylaszonalenin^13^) or farnesylation (drimentine^14^) of the indole C3 can trigger pyrroloindoline formation (Supplementary Fig. 1).

Ribosomally synthesized and post-translationally modified peptides (RiPPs) are a rapidly growing, major class of natural products and can possess strong and varied bioactivities^15,16^. Their biosynthesis begins with the ribosomal expression of a short gene to yield a precursor peptide that usually consists of an N-terminal ‘leader’ and a C-terminal ‘core’ peptide. While the leader peptide is important for substrate recognition by the enzyme(s) installing class-defining modifications, the core peptide is ultimately converted into the final product and can be extensively modified^17–19^. Few pyrroloindoline-containing RiPP classes have been identified to date (for example, omphalotins^20^, ComX^21^ and kawaguchipeptin A^22^), and in each case, a prenyltransferase appears to trigger pyrroloindoline formation as described above^23^. Crocagins (**1** and **2**; Fig. 1b)^24^ are very small, intriguing RiPPs that possess a tetracyclic ring system, which consists of a pyrroloindoline fused to a tetrahydropyrimidinone moiety. This rare scaffold, identified during metabolome mining, is thus far unique among RiPPs^24^. While the biosynthetic gene cluster (BGC) for **1** and **2** could be identified^24^, it does not encode for an enzyme previously associated with pyrroloindoline formation. The steps involved in the biosynthesis of **1** and **2** from the precursor peptide CgnA are unknown, as are the functions of the predicted proteins found in their BGC (Fig. 1c) with the exception of the carbamoyltransferase CgnI^24^. It has been demonstrated that **1** inhibits the highly conserved, key global regulator of the bacterial transcriptome, CsrA, in vitro^24^. CsrA coordinates the expression of a variety of proteins including specific virulence factors as well as biofilm formation and represents a novel target^25–27^.

We were intrigued by the novelty of the scaffold of **1** and **2** and the possibility of establishing an unprecedented biosynthetic route to peptide-derived pyrroloindoline alkaloids. Here, we demonstrate that four proteins, CgnB, CgnC, CgnD and CgnE, are sufficient to produce the core scaffold of **1** and **2** in vitro and propose a biosynthetic scheme for **1** and **2**. We identify CgnB and CgnE as unusual peptide-binding proteins, which enabled genome mining that led to the in silico discovery of putative related and unrelated biosynthetic pathways.

## Results

### In vitro biosynthesis of the tetracyclic crocagin core

It had been reported that the protein CgnB binds to the precursor peptide CgnA, and that a *cgnB* knockout abolished the production of crocagins A and B^24^. Interestingly, a knockout of the *cgnB* homologue *cgnE* (38% sequence identity; Supplementary Fig. 2) resulted in a reduction of crocagin production and the detection of two new species: one had a mass suggestive of **1** unmodified at the Trp Cβ position, while the other agreed with a *des*-*N*-methyl, *des*-carbamoyl **1** (Supplementary Fig. 2).

**Fig. 2:**
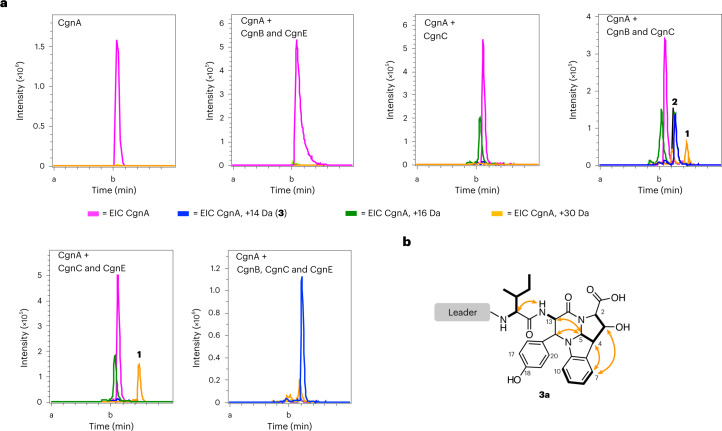
In vitro biosynthesis of the crocagin core structure. **a**, Extracted ion chromatograms (EICs) of CgnA (magenta) and reactions of CgnA with CgnB and CgnE; CgnC; CgnB and CgnC; CgnC and CgnE; or CgnB, CgnC and CgnE (from top left to right). Addition of CgnC with cofactors to CgnA triggered the formation of a minor +16 Da species (green). Adding CgnA to CgnB, CgnE and CgnC with cofactors resulted in the consumption of CgnA (magenta) and the formation of a major product (+14 Da, blue) as well as minor products (+16 Da, green; +30 Da (a and b are peaks of the same mass, but with different retention times), orange). All EICs reflect the +5 charge state of CgnA (mutants) and products ±0.01 Da. Representative experiments were repeated independently at least three times with similar results. Details can be found in Supplementary Fig. 5. **b**, NMR structure of **3a**.

To begin our investigation of the biosynthesis of **1** and **2**, we decided to focus on the operon containing *cgnA*, which encodes the precursor peptide CgnA, and putative biosynthetic gene products CgnB, CgnC, CgnD, and CgnE. CgnC was predicted to be a dioxygenase, and the putative esterase CgnD was a likely candidate for leader peptide removal. We thus initially focused on CgnB, CgnC and CgnE. Incubation of CgnA with either CgnB, CgnE or both did not result in any observable product peak (Fig. 2a and Supplementary Fig. 3). Expression of CgnC required the use of an alternative, upstream start codon (Supplementary Fig. 4), and incubation with CgnA did not lead to observable product formation, unless FeCl_2_, ascorbic acid and α-ketoglutarate were added (Fig. 2a and Supplementary Fig. 5). The addition of these cofactors, always implied in CgnC reactions from this point forward, yielded a new peak with a mass suggestive of hydroxylation (+16 Da), but the predominant peak remained unmodified CgnA (Fig. 2a and Supplementary Fig. 5). The addition of CgnE to CgnA–CgnC led to the formation of an additional, minor peak with a mass shift of +28 Da, which could indicate two oxidation events, for example, oxidation to ketones at the Cβ atoms of the Tyr and Trp residues of the core peptides (Fig. 2a and Supplementary Fig. 5). Intriguingly, the addition of the CgnE homologue CgnB led to a different result, with two additional product peaks detected when compared to CgnA–CgnE or CgnA–CgnC reactions (Fig. 2a and Supplementary Fig. 5). One of these had a mass shift of +14 Da, which could indicate the formation of the tetracyclic core structure (**3a**; Fig. 2a and Supplementary Fig. 5). These data agree with the knockout studies, where the inactivation of *cgnE* led to reduced product formation, while the inactivation of *cgnB* abolished the production of **1** and **2**. The addition of both CgnB and CgnE to CgnA–CgnC reactions had a profound effect and led to the formation of a major product with high turnover (Fig. 2a and Supplementary Fig. 5). This product, **3a**, had a mass shift of +14 Da, the same retention time as that observed for the +14 Da product of CgnA–CgnB–CgnC reactions, and lost its UV absorption at 280 nm, implying loss of aromaticity of the Trp side chain (Supplementary Fig. 5).

Tandem mass spectrometry (MS^2^) of the +16 Da species suggested hydroxylation of the core peptide’s Trp (Supplementary Fig. 6). MS^2^ of the +28 Da species was not possible due to low abundance, but MS^2^ of the +30 Da peak placed +14 Da on the core peptide’s Tyr (keto group) and +16 Da on the core peptide’s Trp residue (hydroxylation; Supplementary Fig. 6). We were able to identify a low abundance +32 Da species, and, as expected, MS^2^ analysis placed hydroxylations on the core peptide’s Tyr and Trp (Supplementary Fig. 6). All mass errors can be found in Supplementary Table 1. A minor product with a mass shift of +12 Da, **3b**, was also observed. We reasoned that this could be an intermediate towards **2**, with a mass of –2 Da compared to crocagin A due to its additional double bond (Supplementary Fig. 5). NMR analysis of **3a** confirmed the presence of the hexahydropyrrolo[2,3-*b*]indole fused to a tetrahydropyrimidinone moiety (Fig. 2b and Supplementary Table 2) and thus the successful in vitro biosynthesis of the tetracyclic crocagin core.

### CgnB and CgnE may have evolved from Zn^2+^-dependent aminopeptidases

**Fig. 3:**
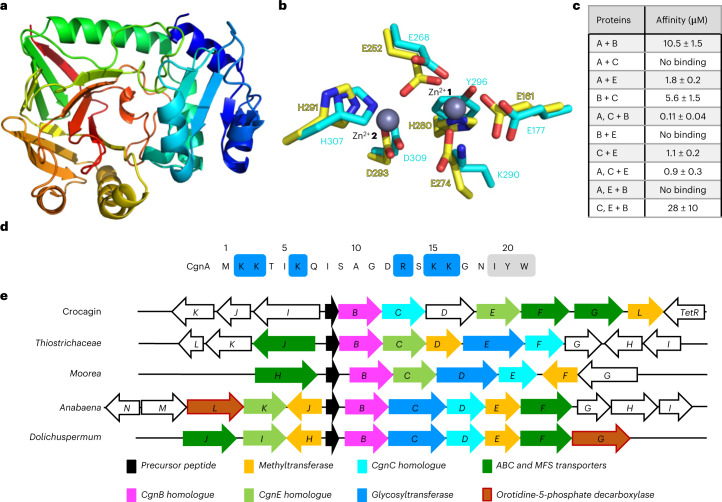
Structures of CgnB and CgnE, affinity measurements and genome mining. **a**, Overall structure of CgnE. Cartoon representation using the rainbow colour scheme (N terminus, blue; to C terminus, red). **b**, Metal binding site found in CgnB (yellow) and CgnE (cyan). Two key residues are mutated in CgnE (Glu to Lys and His to Tyr). Zn^2+^ ions are numbered **1** and **2**. **c**, Table of affinities to investigate substrate binding and complex formation. Components following the ‘+’ are titrated, while the other components are held constant. Representative experiments were repeated independently three times with similar results. **d**, Sequence of CgnA with residues numbered. Positively charged side chains are highlighted in blue, while the core peptide is in grey. **e**, Representatives of the five distinct BGC types identified. Common genes are colour-coded. White arrows represent genes with functions not found in all clusters. ABC, ATP-binding cassette; MFS, major facilitator superfamily. Details are reported in Supplementary Tables 4 and 5.

We were very intrigued by the different behaviour of the homologues CgnB and CgnE in biochemical assays. CgnB and CgnE did not show similarity to any family in the Pfam database, and a sequence-based search using the HHpred server^28^ returned only three hits with more than 50% sequence coverage, which were aminopeptidases (Pfam family Peptidase_M29). We did not observe aminopeptidase activity, and the sequence identities of CgnB and CgnE with the aminopeptidases were <25% (Supplementary Fig. 7). It was thus apparent that structural information would be valuable to better characterize these two proteins. First, the structure of CgnE was determined to 2.0 Å (Fig. 3a and Supplementary Fig. 8). Details for all reported protein structures can be found in the experimental section, and all data collection and refinement statistics can be found in Supplementary Table 3.

CgnB was recalcitrant to crystallization until a slight molar excess of CgnA was added prior to crystallization trials. We determined the CgnB structure to 2.3 Å resolution but observed no electron density for CgnA, so the peptide may simply have acted as an additive to promote crystallization. As expected for homologous proteins, the Cα root-mean-square deviation (r.m.s.d.) between the structures of CgnB and CgnE was only 1.2 Å over 284 residues (Supplementary Fig. 9). Curiously, CgnB contained a dinuclear metal centre that was partially occupied. The CheckMyMetal validation server^29^ suggested that either Zn^2+^ or Co^2+^ could be bound and inductively coupled plasma mass spectrometry (ICP-MS) identified the metals as zinc, with only traces of cobalt found (Supplementary Fig. 10). The binding sites comprised CgnB residues Glu161, Glu252 (shared between Zn^2+^ **1** and **2**), Glu274 and His280 (Zn^2+^ **1**) and His291 and Asp293 (Zn^2+^ **2**; Fig. 3b and Supplementary Fig. 10). In CgnE, the metal binding site of Zn^2+^ **1** was disrupted by mutations in two metal-coordinating residues (CgnB numbering), Glu274Lys and His280Tyr, and no density for metal ions was observed in the CgnE structure (Fig. 3b and Supplementary Fig. 10). To exclude the possibility that CgnE may bind Fe^2+^, which is added as a CgnC cofactor to the reactions and could be oxidized and released from CgnE before structure determination, we incubated the protein with a large excess of fresh FeCl_2_ and processed the sample immediately for analysis by ICP-MS. No iron could be detected (Supplementary Fig. 10).

A search for structural homologues of CgnB and CgnE using the DALI server^30^ returned only three viable hits, which concurred with the sequence-based results obtained from HHpred^28^: aminopeptidase T (Protein Data Bank (PDB) no. 2ayi), aminopeptidase PepS (PDB no. 4ics) and aminopeptidase AMPS (PDB no. 1zjc), which are structural homologues (Cα r.m.s.d. 4ics/1zjc = 2.2 Å; Cα r.m.s.d. 4ics/2ayi = 4.3 Å over >90% of residues; Supplementary Fig. 10). The key difference between CgnB or CgnE and the aminopeptidases was found in the N-terminal portions of the proteins (Supplementary Fig. 11). The aminopeptidases form an extensive dimer interface, and the residues involved in its formation are largely missing in CgnB and CgnE, which rationalizes their monomeric state in the crystals. All three aminopeptidases harbour dinuclear metal centres in the same position as CgnB and contain either Zn^2+^ or Co^2+^. To test the effect of different metal ions on the reaction among CgnA, CgnB, CgnC and CgnE, we dialysed CgnB extensively with ethylenediaminetetraacetic acid and reduced the Zn^2+^ concentration to background (buffer) levels (Supplementary Fig. 10). Unfortunately, the protein denatured in the process, and activity could not be restored.

### Inference of protein complex formation between CgnB, CgnC and CgnE

The requirement for all three components—CgnB, CgnC and CgnE—to be present for efficient catalysis of the formation of **3a** or **3b** implied that the proteins may interact. We probed each protein’s ability to bind the substrate CgnA by microscale thermophoresis (MST) and found that CgnB and CgnE, but not CgnC, were able to bind CgnA (Fig. 3c and Supplementary Fig. 12). The equilibrium dissociation constant (*K*_D_) of 10.5 µM for the CgnA–CgnB interaction is approximately one order of magnitude lower than that measured by surface plasmon resonance^24^ and five times weaker than the one we determined for the CgnA–CgnE interaction (1.8 µM; Fig. 3c). When we investigated the binding of the homologues CgnB and CgnE to CgnC, we found the CgnE–CgnC *K*_D_ to be ~1 µM (Fig. 3c and Supplementary Fig. 13). The CgnB–CgnC *K*_D_ was weaker (~6 µM), and no interaction could be detected for CgnB–CgnE (Fig. 3c and Supplementary Fig. 13). We wondered if the presence of CgnA would change this behaviour and determined the *K*_D_ of CgnC to CgnB in the presence of CgnA (400 µM). The effect was an ~60-fold improved affinity to ~100 nM (Fig. 3c and Supplementary Fig. 13). Addition of CgnA (400 µM) to CgnC–CgnE had very little effect (2-fold increase) on the CgnC–CgnE affinity (Fig. 3c and Supplementary Fig. 14). It therefore appeared that CgnB and CgnE might be recruiting the substrate CgnA to CgnC. When we analysed the binding of CgnA to CgnB in the presence of a large excess of CgnE (250 µM), we did not observe binding (Fig. 3c and Supplementary Fig. 13). This implied that CgnB and CgnE cannot bind simultaneously to the same substrate molecule. To support our MST data, we attempted pull-down assays. While these confirmed the interactions between CgnA and CgnB or CgnE, we could not find conditions that prevented CgnC from binding non-specifically to resin materials. The same was observed for surface plasmon resonance measurements (Supplementary Fig. 13).

Size-exclusion chromatography data suggested that CgnB and CgnE were monomers in solution (as observed in the crystal structures), while CgnC was a dimer (Supplementary Fig. 13). Unfortunately, putative CgnB–CgnC–CgnE complexes were unstable in size-exclusion chromatography, and experiments to stabilize them for structure elucidation and detailed mechanistic studies are currently underway. The question we then asked was whether binding of CgnB and CgnE would occur at the same binding site on CgnC, as might be expected for homologues. We thus determined the affinity of CgnB–CgnC in the presence of a constant, large excess (250 µM) of CgnE. We obtained a *K*_D_ of 28 µM, which was approximately five times weaker than in the absence of CgnE, but these data still suggested that CgnB and CgnE can concurrently bind to CgnC (Supplementary Fig. 13). Taken together, our data imply that a complex may form during crocagin biosynthesis. If CgnC bound CgnB and CgnE at the same time, as MST measurements suggest it could, the dimeric nature of CgnC would likely result in a complex with the stoichiometry CgnB_2_/CgnC_2_/CgnE_2_ and have the ability to bind up to four CgnA molecules.

### Genome mining using CgnB and CgnE reveals crocagin-like BGCs

The unique features of CgnB and CgnE prompted us to investigate their evolution. Sequence similarity searches in bacterial whole-genome sequencing contigs revealed that CgnB and CgnE homologues can be found in ~100 genomes from Actinobacteria, Cyanobacteria, Betaproteobacteria, Gammaproteobacteria and Deltaproteobacteria. In all cases, the similarity stretched over almost the entire protein length, and the homologues did not contain any additional domains, which makes CgnB and CgnE the founding members of a new protein family.

In all genomes containing only one copy of a CgnB or CgnE homologue, the metal binding site was intact, despite an overall low sequence identity (between 21% and 42%). Finding two CgnB and/or CgnE homologues in the same genomic contig was much rarer (Supplementary Table 4), and in all cases, these two copies lie at a distance <2.1 kbp. To investigate the evolution of the duplicated CgnB and CgnE homologues, we constructed a bootstrapped maximum likelihood phylogenetic tree (Supplementary Fig. 14 and Supplementary Information). Low bootstrap values did not allow many internal branches to be resolved with certainty, but it appears probable that three to five duplication events occurred during the evolution of this protein family. In every case, the second copy of the protein acquired deleterious mutations in the metal binding site that were different in each case. Mutations designed to restore metal binding in CgnE or disrupt metal binding in CgnB led to insoluble protein for all mutations tested. It is remarkable that at least three duplication events happened independently, which may be a sign of strong evolutionary pressure to acquire a second, mutated copy of the protein.

Genome mining for novel RiPPs using instances of a single copy of CgnB or CgnE with the program RiPPER^31^ resulted in the discovery of a number of putative precursor peptides (Supplementary Data), which were used to create a sequence similarity network (Supplementary Fig. 15). In genomic contigs harbouring two CgnB or CgnE copies, we searched for a potential precursor and were able to find a candidate within <1.5 kbp in all cases (Supplementary Table 4). Aligning these candidate peptides at their C termini revealed a strong conservation pattern for the Tyr and Trp positions of the core peptide (Ile-Tyr-Trp for crocagin; Supplementary Table 4). The Ile position is variable, and we found an Ile to Gly mutation to be a very good substrate for CgnB, CgnC or CgnE (Supplementary Fig. 16). Two other single leader peptide point mutations in highly conserved positions also had no effect on processing (Lys2Glu and Gly17Phe; Supplementary Fig. 16).

The leader peptide is enriched with positively charged amino acids, and both CgnB and CgnE show a large, strongly negatively charged patch near the possible binding site (Supplementary Fig. 16). Of the six positively charged residues in the 18-amino-acid-long CgnA leader peptide (CgnA^LP^), three are found in the N-terminal half, while the other three are close to the core peptide (Fig. 3d). Deleting residues 1–5 of CgnA (and thus two lysines; Fig. 3d) had no effect on turnover, but removing residues 1–10, which include a third lysine, abolished processing (Supplementary Fig. 17). To analyse the importance of the positively charged residues, we reacted a CgnA variant with the three N-terminal lysines mutated to alanine (CgnA^N3A^; Lys2Ala, Lys3Ala and Lys6Ala) with CgnBCgnC–CgnE and found the reaction to be somewhat impaired (Supplementary Fig. 17). Exchanging the three C-terminal positively charged residues to alanine (CgnA^C3A^; Arg13Ala, Lys15Ala and Lys16Ala), on the other hand, severely reduced product formation (Supplementary Fig. 17). These data imply that essential CgnA residues for substrate recognition are located after position five and include four positively charged residues.

The structures of the putative crocagin-like BGCs identified here differ in all genomes, but one can identify five key architectures (Fig. 3e). The crocagin BGC is an outlier, and intriguingly all other clusters contain a predicted glycosyltransferase instead of the carbamoyltransferase present in the crocagin BGC. This suggests that crocagin-like natural products may be glycosylated, which has thus far been a very rare modification in RiPPs. Some BGCs contain a predicted decarboxylase, which may remove the negative charge of crocagin-like molecules and thus improve membrane permeability in addition to providing biosynthetic access to new structural variants.

### Protease CgnD releases the crocagin core scaffold

**Fig. 4:**
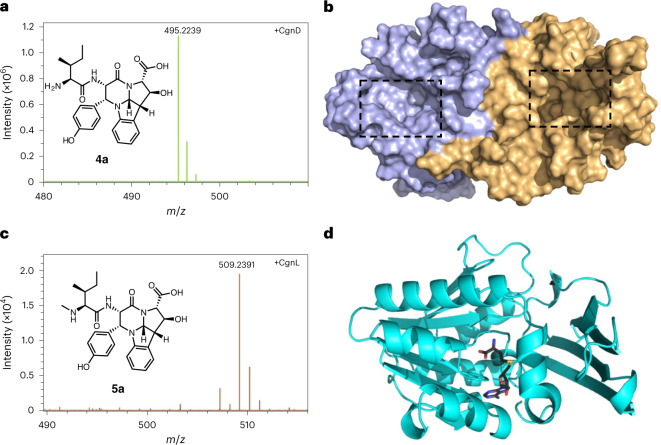
Processing of 3a and 3b by CgnD and CgnL. **a**, In vitro biosynthesis of the crocagin A core scaffold, **4a**, using CgnD. *m/z* represents mass divided by charge number and the horizontal axis in a mass spectrum is expressed in units of *m/z*. **b**, Surface representation of the CgnD dimer. Active sites are highlighted by dashed boxes. **c**, *N*-methylation of **4a** by CgnL to yield **5a**. **d**, Cartoon representation of the CgnL structure. *S*-adenosylhomocysteine is shown as black sticks. Details of **a** and **c** can be found in Supplementary Figs. 18 and 21. Representative experiments were repeated independently at least three times with similar results.

The product of the CgnB–CgnC–CgnE reaction, **3a**, contained the crocagin tetracyclic ring system, and we thus suspected that proteolytic removal of the leader peptide by CgnD might be possible. When CgnD was added to the product of CgnA–CgnB–CgnC–CgnE reactions containing **3a** and **3b**, we observed the slow production of **4a** ([M]^+^ calculated, 495.2238; observed, 495.2239; mass error (*Δ*) = 0.2 ppm), which represents the crocagin core scaffold (*des*-methyl and *des*-carbamoyl) and also corresponds to the mass produced by the *cgnE* knockout (Fig. 4a and Supplementary Figs. 2 and 18). Interestingly, we again observed a minor peak of –2 Da (**4b**; [M]^+^ calculated, 493.2082; observed, 493.2083; *Δ* = 0.2 ppm), which may be the core scaffold of crocagin B (Supplementary Fig. 18). Slow proteolytic processing of modified precursor peptides has been observed in a number of RiPP biosynthetic pathways and may be linked to biosynthetic timing^32^. In an attempt to accelerate the production of **4a**, we replaced the Asn residue preceding the core peptide with a Lys. This mutant was a very poor substrate for CgnB–CgnC–CgnE, possibly because the introduced positive charge interfered with native substrate binding. The addition of trypsin did lead to the formation of traces of **4a** (Supplementary Fig. 18). Rapid production of crocagin-like molecules will thus require the selection of alternative proteases or enzyme engineering of CgnD. To rationalize the slow turnover, we attempted to determine the affinity of CgnD for **3a**, CgnA and the leader peptide, but were unable to detect binding by microscale thermophoresis (Supplementary Fig. 19). We hoped that the crystal structure of CgnD might yield additional insights and determined the structure to 2.35 Å resolution (Supplementary Fig. 20). The two protomers found in the asymmetric unit form an intricate dimer interface (Supplementary Table 6), which is unlike that of the closest structural homologues identified by a Dali server^30^ search. Intriguingly, it appeared as if the dimer provides two antiparallel channels for substrate binding that terminated at the respective active sites (Fig. 4b). The catalytic triad comprised Asp325, His328 and Ser79, and the distance observed between the Asp325 atom Oδ2 and His328 atom Hδ2 was within the expected range (1.8 Å). The distance between His328 atom Nε2 and Ser79 atom Hγ could not be determined because a sulfate ion was bound at the active site, which led to a rotation of the Ser side chain. Removal of the sulfate in silico and subsequent rotation of the Ser side chain did not allow the distance to decrease below 2.8 Å (Supplementary Fig. 20). The resulting lack of nucleophilicity appeared a probable cause for the slow turnover and was supported by our inability to label CgnD with phenylmethylsulfonyl fluoride (Supplementary Fig. 20). This situation is reminiscent of PatA from the patellamide pathway, which also turns over very slowly^32^.

### *N*-methylation of 4a and 4b is catalysed by CgnL

The final unresolved step in the biosynthesis of **1** and **2** was the *N*-methylation of **4a** and **4b**, as the carbamoyltransferase function of CgnI had been determined previously^24^. Based on sequence alignments, CgnL, named ‘MT’ in the original crocagin publication^24^, was the likely candidate. Indeed, incubation of **4a** and **4b** with CgnL and *S*-adenosylmethionine led to the formation of **5a** ([M]^+^ calculated, 509.2395; observed, 509.2391; *Δ* = –0.8 ppm) and the –2 Da **5b** ([M]^+^ calculated, 507.2238; observed, 507.2239; *Δ* = 0.2 ppm; Fig. 4c and Supplementary Fig. 21). The mass increase of +14 Da suggested methylation and thus the production of *des*-carbamoyl **1** and **2**.

The crystal structure of CgnL in complex with *S*-adenosylhomocysteine was determined to a resolution of 1.96 Å and appeared monomeric, which was consistent with the closest structural homologues of CgnL identified in a Dali search^30^. The overall shape of CgnL was typical for class I *S*-adenosylmethionine-dependent methyltransferases with a Rossman fold (Fig. 4d and Supplementary Fig. 22). The electron density for *S*-adenosylhomocysteine was unambiguous as expected for a well-coordinated ligand (Supplementary Fig. 22). Interestingly, the closest structural homologues with a defined function are involved in natural product biosynthetic pathways: CcbJ (PDB no. 4hh4) *N*-methylates the antibiotic celesticetin^33^, while KedS8 (PDB no. 5bsz) *N*-methylates the chromophore of the anticancer chromoprotein kedaricidin^34^.

### A proposed mechanism for the biosynthesis of 3a and 3b

**Fig. 5:**
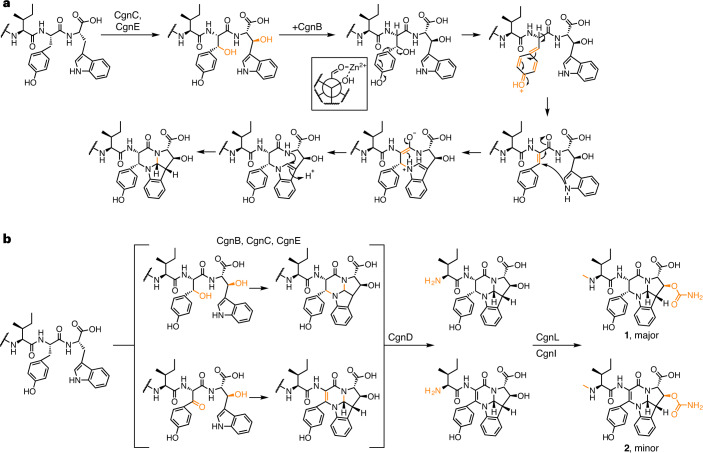
Biosynthetic proposal leading to the formation of crocagins A (1) and B (2). **a**, Proposed mechanism for installation of the tetracyclic crocagin core. **b**, CgnB, CgnC and CgnE install two β-hydroxylations and facilitate the two ring-closure reactions. CgnD cleaves the core scaffold off the leader peptide, which is subsequently methylated and carbamoylated. Crocagin B, the low abundance crocagin in extracts from the natural producer, appears to originate from oxidation before ring closure during the biosynthesis of **3a** to yield **3b**.

The precise molecular mechanism of the initial step in crocagin biosynthesis, leading to the formation of **3a** and **3b**, will require further study. We favour the following sequence of events based on the knockout, biochemical and binding data (Fig. 5a): CgnA binds to CgnE, which may either be part of a CgnC–CgnE complex, or bind to CgnC after binding CgnA. We found the binding of CgnE to the CgnA^LP^ to be almost 300 times weaker than binding to CgnA (*K*_D_ = 514 µM; Supplementary Fig. 23). The main driver for binding thus appears to be the core peptide, and it is tempting to suggest that CgnE’s role may be to recruit the substrate peptide to CgnC and present the core peptide in a particular way that enables or accelerates correctly timed catalysis. This would agree with our knockout and in vitro biochemical data, which show that the presence of CgnE without CgnB does not enable formation of the crocagin core structure. When we tested the mutant CgnA Y20F, we could only detect trace amounts of a +14 Da species, and the major product peak had a mass increase of 16 Da. (Supplementary Fig. 23). Unlike the +16 Da product of a CgnA–CgnC–CgnE reaction (Trp hydroxylation; Supplementary Fig. 6), MS^2^ analysis of this intermediate revealed a mixture with hydroxylation of either Phe or Trp (Supplementary Fig. 24). Hydroxylation at the Tyr20 Cβ position allows CgnB to catalyse an elimination reaction via its bound Zn^2+^ (or Co^2+^) ions, which results in a double bond between the Tyr20 Cα and Cβ atoms, creating a conjugated amine. The transition state, in which the Tyr20 Cα hydrogen would possess a lowered p*K*_a_, could be stabilized by the Tyr side chain, rationalizing why a Tyr20Phe mutation is detrimental to turnover (Supplementary Fig. 23). Tyr20 then undergoes conjugate addition with the Trp21 indole nitrogen, and subsequent proton transfer completes the first ring-forming reaction. The second ring-closure reaction between the Trp α-nitrogen and δ_1_ carbon atom could then occur spontaneously to complete the formation of the tetracyclic ring system. It is unclear at which point hydroxylation of the Trp21 Cβ position occurs, but the inferred biosynthetic complex would enable fast transfer from one substrate binding site to the next. Interestingly, CgnA binding by CgnB is not dependent on the core peptide to the same extent as observed for CgnE, with an 8-fold reduction in affinity for CgnA^LP^ compared to CgnA (Supplementary Fig. 23).

It has been observed in a number of RiPP systems that the leader peptide can activate the processing enzymes when it is supplied *in trans*^35^. This was not the case for CgnB–CgnC–CgnE reactions, where turnover of the core peptide was marginal when the leader peptide was added *in trans* (Supplementary Fig. 23). This suggests that a physical connection between leader and core peptide is essential for catalysis, perhaps to enable efficient transfer of the substrate between CgnE and CgnB.

## Discussion

It is intriguing to see how the world of small alkaloids merges with that of ribosomal natural products. The recent discovery that the pyrroloquinoline alkaloids ammosamides are in fact RiPPs^36^ and crocagins may just be the beginning. We propose the following biosynthetic scheme for crocagins (Fig. 5b): After expression of CgnA, the precursor peptide is processed by the proteins CgnB, CgnC and CgnE and undergoes hydroxylation at the Tyr20 and Trp21 Cβ positions, which triggers the two ring-closing reactions. It is possible that pyrroloindoline formation is the first ring-closure reaction, but we think that the seeming absence of an indole ‘activator’ (for example, C3 prenylation; Supplementary Fig. 1) and the disfavoured nature of 5-*endo*-trig ring-forming reactions makes it more likely that the pyrroloindoline forms second. In a small fraction of CgnA, the hydroxylated Tyr20 Cβ is oxidized to the ketone, which results in a double bond between the Tyr20 Cα and Cβ upon ring closure and thus ultimately the formation of **2**. This reaction would not be catalysed by CgnB, but rather be spontaneous and slow. In our view, it is likely that the observed CgnA +28 Da peak is a shunt product and an artefact of a system missing either CgnB or CgnE. It is unclear if the CgnA +16 Da peak is a shunt product or true intermediate, because we do not observe it when CgnB and CgnE are both present. The +30 Da peak, which we observe only in reactions where **3a** and **3b** are formed, appears to be an intermediate towards **2**, because MS^2^ analysis placed the keto group on the core peptide’s Tyr, while the Trp is hydroxylated (Supplementary Fig. 6). The intermediates **3a** and **3b** are then cleaved by CgnD to release the core scaffold and make the N terminus available for methylation by CgnL. It remains unclear if *N*-methylation or carbamoylation occurs first, or whether both modifications are independent of each other, since the *des*-carbamoyl **1** can be observed after knocking out *cgnI* in vivo^24^. It is also possible that carbamoylation occurs prior to leader peptide cleavage, which would offer an alternative explanation for the slow turnover of CgnD that we observed.

CgnE appears to recruit the substrate peptide CgnA to CgnC and assist in catalysis, perhaps by presenting the core peptide in a specific conformation or by ensuring appropriate timing of the hydroxylation reactions, but does not seem to possess catalytic activity itself. The CgnE homologue CgnB on the other hand appears to possess catalytic activity. Unfortunately, all attempts to obtain crystal structures of CgnA–CgnB or CgnA–CgnE complexes, which would reveal more details of the interactions, failed. A search for CgnB and CgnE in available genomic data places them in a variety of genomes that warrant further investigation. In all cases where two copies were found, they were placed in a crocagin-like BGC with a CgnC homologue, and we thus expect them to install the hallmark tetracyclic core. While these BGCs also contained a methyltransferase, likely to cap the N terminus as observed in crocagins, we were surprised by the variety of different putative modifying enzymes. These may be used for the modification of the hydroxyl group found at the Trp Cβ (glycosylation), the Trp carboxy group (decarboxylation) or the Tyr hydroxyl group. Further studies will be required to identify the natural products of these BGCs, explore their bioactivities and link predicted open reading frames to biosynthetic functions. Interestingly, the gene annotated as G in the BGC identified in *Anabaena* (Fig. 3d) shares 61% sequence identity with a bifunctional 6′-aminoglycoside-*N*-acetyltransferase/aminoglycoside-2′-phosphotransferase^37^, which confers broad-spectrum resistance to aminoglycoside antibiotics. It may serve as a self-resistance gene and hint at the bioactivity of glycosylated crocagin derivatives.

It will be fascinating to investigate the crocagin biosynthetic system in more detail, as these data could then be used in conjunction with enzymes from the newly discovered crocagin-like BGCs to access a structurally diverse family of peptide-derived pyrroloindoline alkaloids.

## Methods

The Supplementary Methods contain additional information on protein purification, production, crystallography and biophysical and biochemical methods, as well as bioinformatics.

### Protein purification of CgnB, CgnC, CgnD, CgnE and CgnL

For protein purification, the cell pellets were resuspended in the respective lysis buffers (for CgnD and CgnE, 500 mM NaCl, 20 mM Tris buffer with pH 8.0, 20 mM imidazole, 3 mM β-mercaptoethanol; for CgnB, CgnC and CgnL, 200 mM NaCl, 20 mM Tris, 20 mM imidazole, 10% glycerol, 3 mM β-mercaptoethanol). For every 25 g of wet cell mass, 100 ml of lysis buffer was added and supplemented with two cOmplete ethylenediaminetetraacetic-acid-free protease inhibitor tablets (Sigma-Aldrich) and 4 mg g^−1^ DNase (Sigma-Aldrich). Cell lysis was carried out via passage through a cell disruptor (30 kpsi, Constant Systems), and the cell debris was removed by centrifugation (43,000*g*, 15 min, 4 °C). The supernatant was decanted, filtered through a 0.45 µm filter and applied to a HisTrap HP 5 ml column (GE Healthcare) pre-equilibrated in lysis buffer at a flow rate of 5 ml min^−1^. The column was extensively washed with 150 ml lysis buffer, and the target protein was eluted using lysis buffer supplemented with 250 mM imidazole. The proteins were passed over a desalting column (16/10, GE Healthcare) at a flow rate of 10 ml min^−1^, pre-equilibrated in lysis buffer. To remove the His-SUMO tag, the desalted protein was incubated with TEV protease for 14 h and 4 °C at a 1:10 mass ratio of TEV/target-protein. Subsequent passage of the solution over a 5 ml HisTrap HP column allowed for a separation of the digested target protein and the His_6_-tagged SUMO. The proteins were then passed over a Superdex 200 16/600 size-exclusion column (GE Healthcare) at a flow rate of 1 ml min^−1^, pre-equilibrated in gel filtration buffer (150 mM NaCl, 10 mM HEPES buffer, 0.5 mM TCEP reducing agent, pH 7.4). The resulting peak was collected and concentrated to the desired concentration using a 30 kDa cut-off filter (Thermo Scientific). The protein concentration was determined using photometric analysis (Nanodrop 2000, Thermo Scientific) and subsequently analysed by sodium dodecyl-sulfate polyacrylamide gel electrophoresis. Selenomethionine variants of CgnD and CgnE were purified analogously to the native proteins.

### Biochemical reactions

All biochemical reactions were performed in 150 mM NaCl, 10 mM HEPES, 0.5 mM TCEP, pH 7.4. For CgnA–CgnB–CgnC reactions, a mixture of 50 µM CgnA, 10 µM CgnB, 5 µM CgnC, 4 mM ascorbic acid, 2.5 mM FeCl_2_ and 1 mM 2-oxoglutaric acid was incubated at 37 °C for 2 h. For CgnA–CgnB–CgnC–CgnE reactions, a mixture of 50 µM CgnA, 10 µM CgnB, 5 µM CgnC, 5 µM CgnE, 4 mM ascorbic acid, 2.5 mM FeCl_2_ and 1 mM 2-oxoglutaric acid was incubated at 37 °C for 2 h. The standard reaction volume was 50 µl. Proteins were precipitated by adding 50% acetonitrile and freezing the mixture at −80 °C for 2 h prior to liquid chromatography and mass spectrometry (LC-MS) analysis.

CgnD activity was analysed by adding 20 µM CgnD to a 100 µM solution of purified **3a** and **3b** and incubating this mixture at 37 °C for up to 96 h. Remaining enzyme was precipitated by adding 50% acetonitrile and freezing the mixture at −80 °C for 2 h prior to LC-MS analysis. CgnL reaction was performed by adding 20 µM CgnL to 100 µM **4a** and 4**b** (product of CgnD reaction), supplemented by 1 mM *S*-adenosyl-l-methionine and 1 mM MgCl_2_. The mixture was incubated at room temperature for 24 h. Remaining enzyme was precipitated by adding 50% acetonitrile and freezing the mixture at −80 °C for 2 h prior to LC-MS analysis.

For large-scale purification of **3a** and **3b**, three 9 ml reactions of the aforementioned condition were pooled and precipitated by addition of 50% acetonitrile and the mixture being frozen at −80 °C for 16 h. Thawed samples were centrifuged at 3,000*g* for 20 min at room temperature. The supernatant was removed and sent to Protein Peptide Research for high-performance liquid chromatography purification.

### Reporting summary

Further information on research design is available in the Nature Portfolio Reporting Summary linked to this article.

## Online content

Any methods, additional references, Nature Portfolio reporting summaries, source data, extended data for the sequence similarity network, supplementary information, acknowledgements, peer review information; details of author contributions and competing interests; and statements of data and code availability are available at 10.1038/s41557-023-01153-w.

## Supplementary information

Supplementary Information Supplementary Figs. 1–24, Tables 1–8 and Methods.

Reporting Summary

Supplementary Data 1 Phylogenetic tree of CgnB and CgnE homologues with accession codes.

Supplementary Table 1 Detailed data for sequence similarity network in Supplementary Fig. 15 (sheet 1). List of reagents and primer (sheet 2).

Supplementary Code 1 Custom scripts used for genome mining in this study.

## Data Availability

Data supporting the main findings of this work are available within the Article and Supplementary Information. Diffraction data and refined structural models (Supplementary Table 3) have been deposited to the PDB: CgnB (6zsv), CgnD (8a2n), CgnE (6zsu) and CgnL (7pd7). The very large raw mass spectrometry data files are available from the authors upon request.

## Code availability

The code/scripts used for genome mining are available in Supplementary Code 1.
