# Supplementary material for: Unusual peptide-binding proteins guide pyrroloindoline alkaloid formation in crocagin biosynthesis
Source: Nat Chem. 2023 Mar 9;15(4):560–8. doi: 10.1038/s41557-023-01153-w (PMC10070186; doi:10.1038/s41557-023-01153-w)
Supplement: Supplementary file 1 — Supplementary Figs. 1–24, Tables 1–8 and Methods. [file 41557_2023_1153_MOESM1_ESM.pdf]

nature chemistry

Article https://doi.org/10.1038/s41557-023-01153-w

Unusual peptide-binding proteins guide pyrroloindoline alkaloid formation in crocagin biosynthesis

In the format provided by the authors and unedited

Unusual Peptide-Binding Proteins Guide Pyrroloindoline Alkaloid Formation in Crocagin Biosynthesis

Sebastian Adam, Dazhong Zheng, Andreas Klein, Carsten Volz, William Mullen, Sally L. Shirran, Brian O. Smith, Olga V. Kalinina, Rolf Müller, Jesko Koehnke

Table of Content

Supplementary Figure 1 3
Supplementary Figure 2 4
Supplementary Figure 3 5
Supplementary Figure 4 6
Supplementary Figure 5 7
Supplementary Figure 6 8
Supplementary Figure 7 9
Supplementary Figure 8 10
Supplementary Figure 9 11
Supplementary Figure 10 12
Supplementary Figure 11 13
Supplementary Figure 12 14
Supplementary Figure 13 15
Supplementary Figure 14 16
Supplementary Figure 15 17
Supplementary Figure 16 18
Supplementary Figure 17 19
Supplementary Figure 18 20
Supplementary Figure 19 21
Supplementary Figure 20 22
Supplementary Figure 21 23
Supplementary Figure 22 24
Supplementary Figure 23 25
Supplementary Figure 24 26

METHODS 27

Cloning of Cgn constructs used in this study 27
Small scale protein expression tests of Cgn constructs 27
Ni^{2+} pull-down assay 28
Surface Plasmon Resonance (SPR) 28
Large scale protein expression of Cgn constructs 28
Seleno-methionine expression of CgnD and CgnE 29
Crystallization and data collection of CgnB, CgnD, CgnE and CgnL 29
Data processing, structure determination, refinement and structural analysis of Cgn proteins 30
NMR Spectroscopy 31
LC-MS analysis of reactions with CgnL, truncated CgnA, $CgnA^{N3A}$ and $CgnA^{C3A}$ 33
Inductively Couple Plasma-Mass Spectrometry 34
Bioinformatics 34
Supplementary Table 1 35
Supplementary Table 2 37
Supplementary Table 3 38
Supplementary Table 4 39
Supplementary Table 6 41
Supplementary Table 7 42
Supplementary Table 8 43

REFERENCES 44

FULL, UNPROCESSED GELS 45

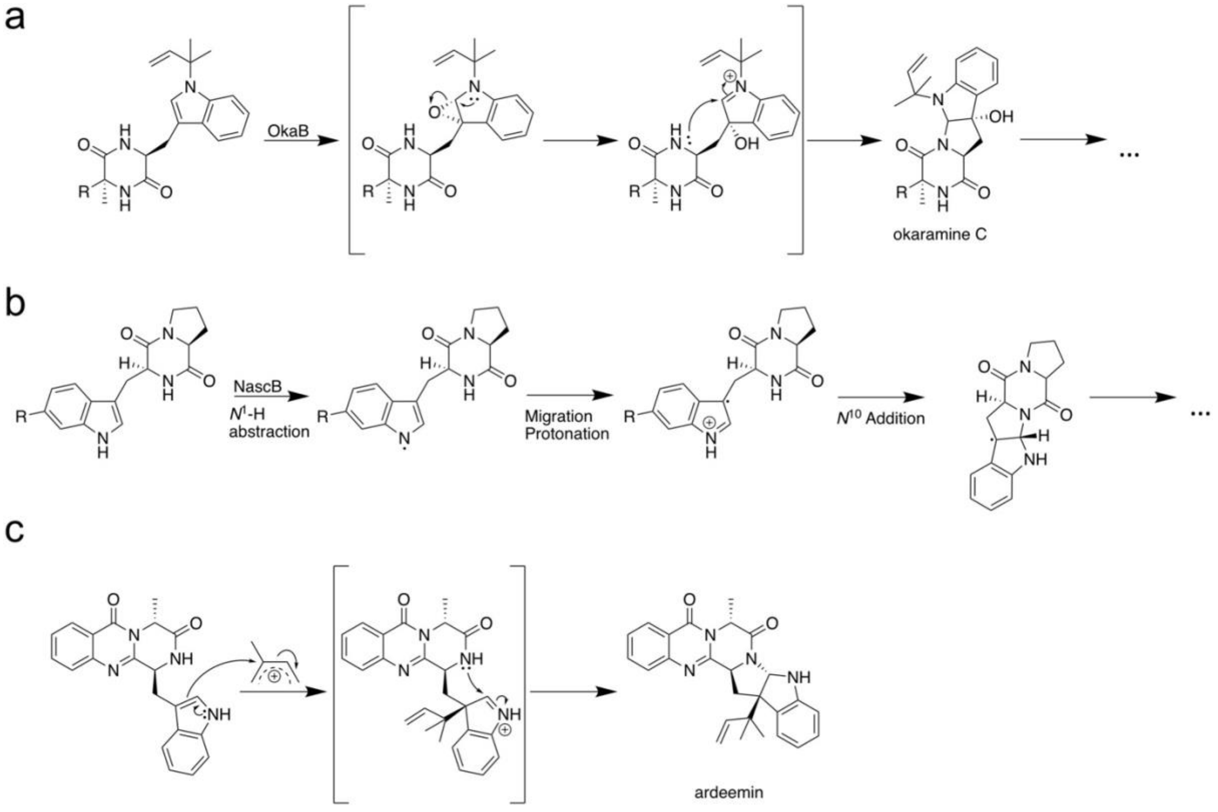

Supplementary Figure 1: Routes to pyrroloindoline alkaloids using okaramines (radical, a), naseseazines (epoxidation, b) and ardeemin (addition, c) as examples.

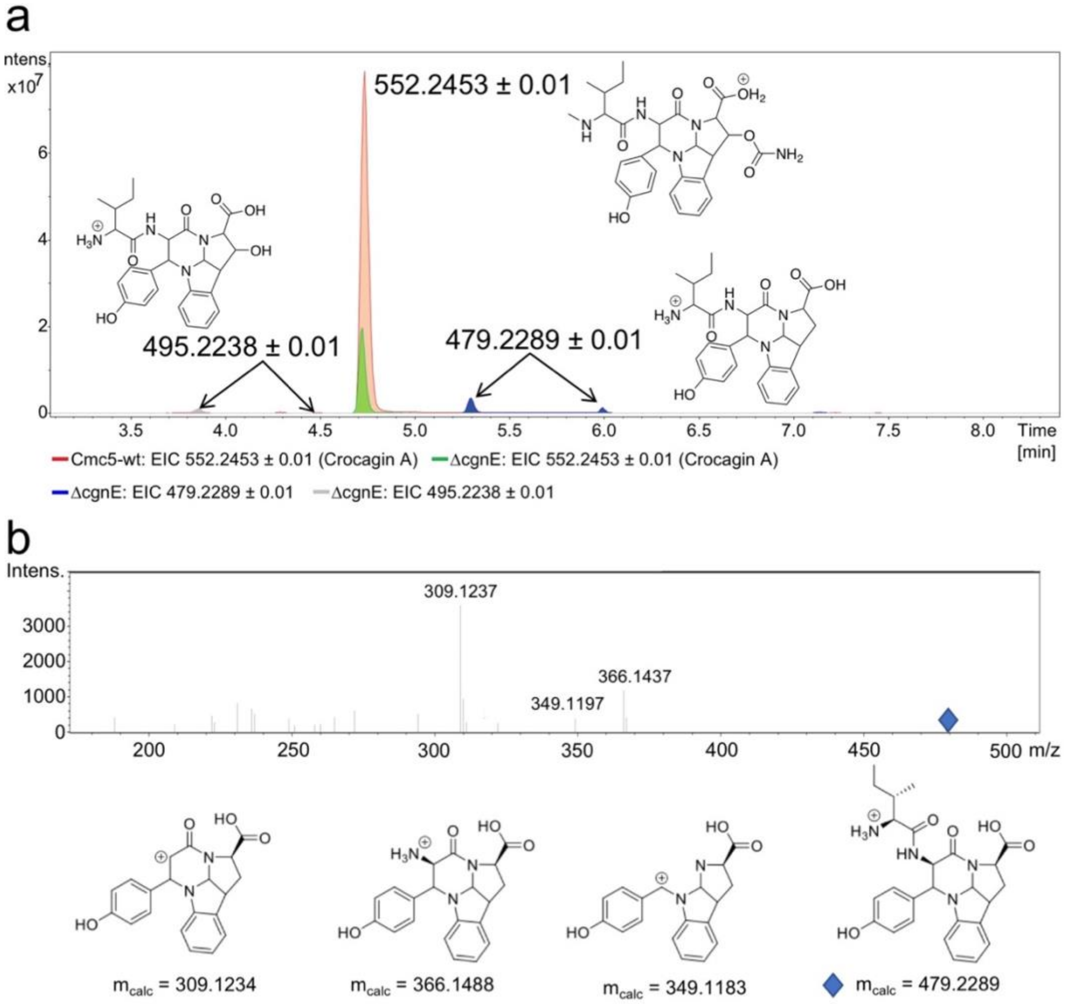

c

```
CgnB        MDVLEYFERLKNRELAFVLDDLQLSDMVTRRGFSVIPFDDFDLAREDHPPAFVLVTRLDY     60
CgnE        MNASDFYALLRGRGMPVVVDDAEAAAVVSELGFRTVPFEAFDFDSPSEDPALVIVAQMGN     60
            *:. :::  *:.* : .*:** : : :*:. ** .:**: **:    .. **:*:*:::.

CgnB        HGKLMQAWETAKGISSHLSLAKFDTSPKSVEYSLDQLLSMDFAETLKRRGDYYDSVASTN     120
CgnE        VDALHGLWERSGTPLMHLALAKFDGGLSRLRAGLARVLAVDTDAALKRRAEAYEQLFSSA     120
             . *   ** :     **:***** . . :. .* ::*::*   :****.: *:.: *:

CgnB        RMEVVTPGAVLTCDFGNEIEIANNDVEMQKGWLYSVAEFFETSVINLEADRSSYTLNGDL     180
CgnE        SVEIASGEGVLRCHIGDEVEVGNCGDTLEQGFLYSVAEFLEASVVNLEGERSTFWVEGEL     180
             :*:.:   .** *.:*:*:*:.* .   :::*:*******:*:**:***.:**:: ::*:*

CgnB        CFTGLIYLCNRPDLKERASATMDELMRMSTRGRNVVSFVDNQIVRMELGGVDMTATLREL     240
CgnE        PFDGFIHLSNSAALKERWGGMLDEFMRRSREGANLVRFADNVIDRLVVGGVDVTSALAGL     240
             * *:*:*.*   **** .. :**:** * .* *:* *.** * *: :****:*::*  *

CgnB        IVGKEREGSSTEFAMGCVEYPLAQDWTINSVMNEGSHGIHVGVGMGKEIPHMDFIAKGAE     300
CgnE        SQGEERGMAATEFGLGCADAEAAEPFGVNSLLHKSAGGAYIGIGKGLRIPHIDFIARGAT     300
              *:**   ::***.:**.:    *: : :**::::.: * ::*:* * .***:****:**

CgnB        LRIAESSDA   309
CgnE        IRFIPAAEG   309
            :*:  :::.
```

Supplementary Figure 2: *cgnE* knock-out and sequence alignment of the proteins CgnB and CgnE. a Comparison of wild-type and *cgnE* knock-out strains' production of crocagin. The cgnE knock-out was generated and analyzed as reported for the *cgnB* knock-out previously.[1] Structures corresponding to the masses are shown. b MS^2 analysis of the main new peak observed in the *cgnE* knock-out strain. c Alignment of CgnB and CgnE. The sequence identity was calculated to be 38%. The sequence alignment was generated with Clustal Omega.[2] Representative experiments were repeated independently three times with similar results.

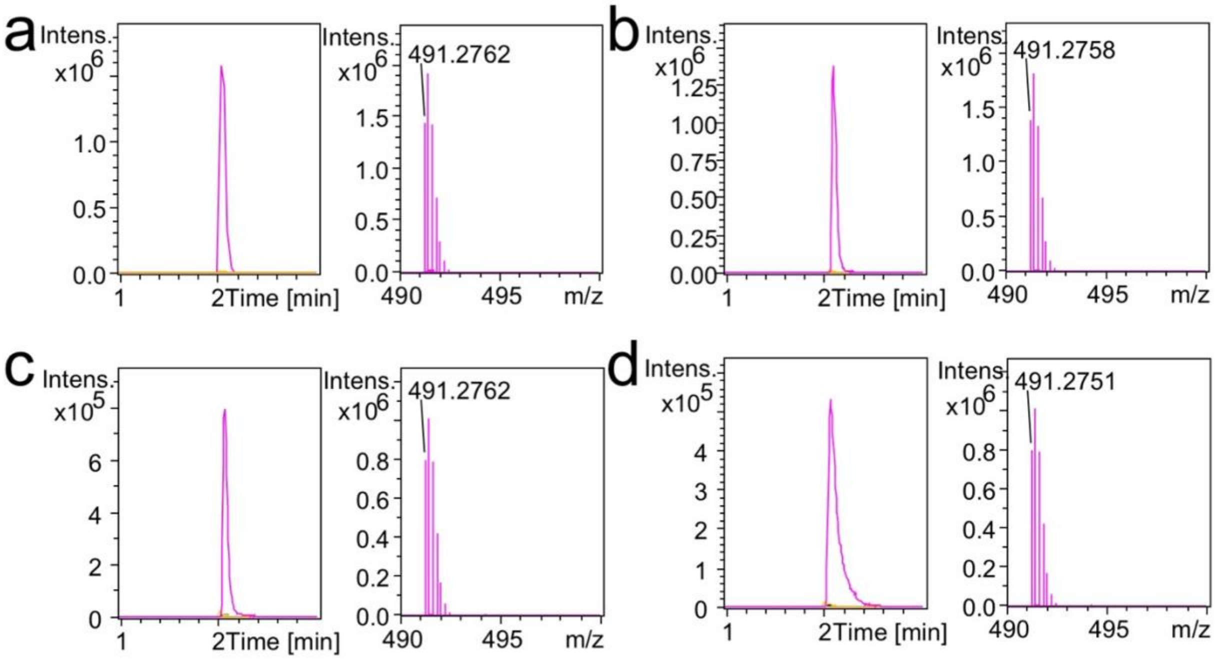

Supplementary Figure 3: Extracted ion chromatograms (EICs) and mass spectra of CgnA (magenta, a) and reactions of CgnA with CgnB (b), CgnE (c) or CgnB/E (d). EICs for CgnA (magenta) and possible products (blue, yellow, and orange) are shown in each EIC panel (± 0.01 Da). No reaction was observed for these peptide/protein combinations and the product EICs are thus flat lines. All mass errors are shown in Supplementary Table 1. Representative experiments were repeated independently at least three times with similar results.

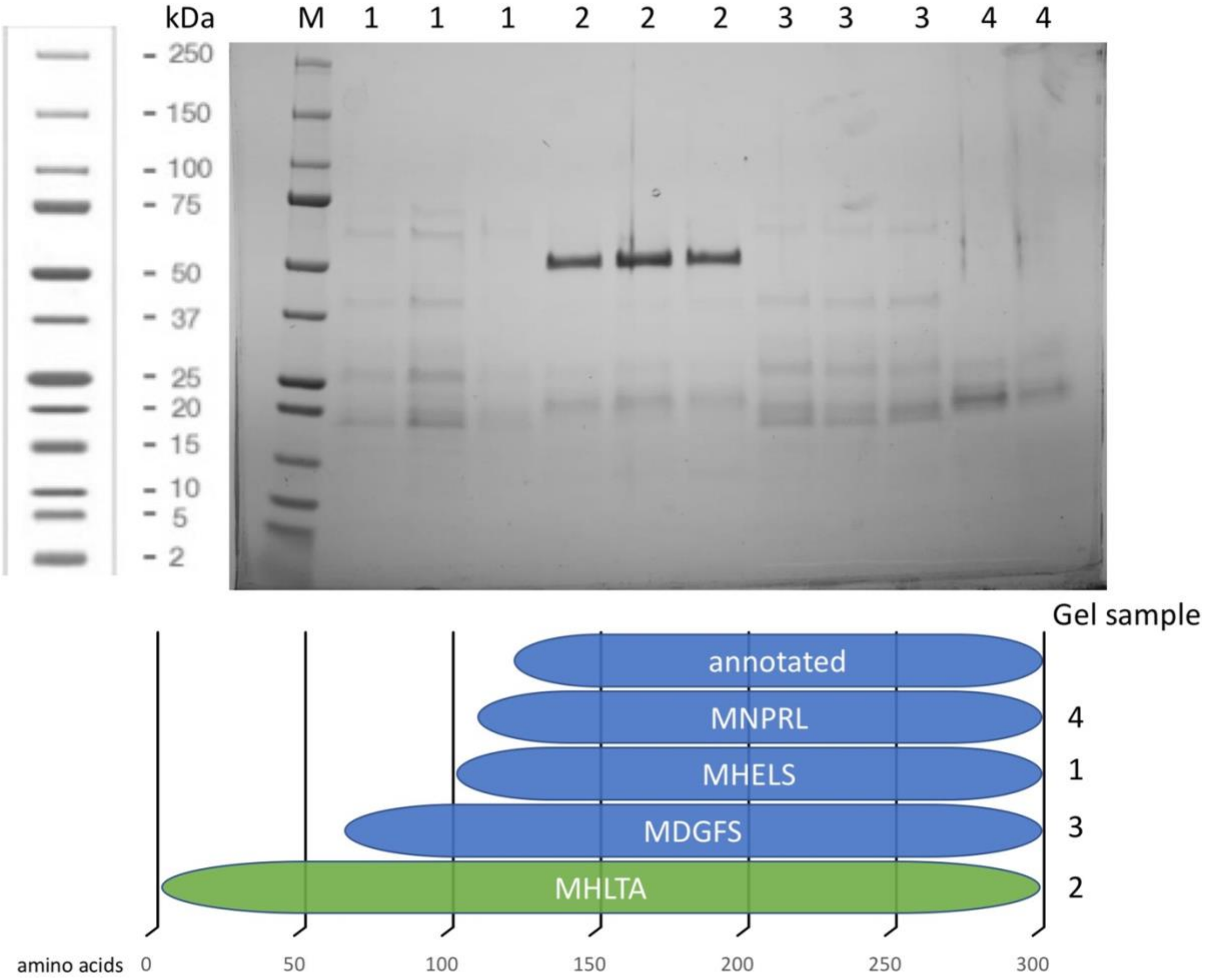

Supplementary Figure 4: Alternative start codons for CgnC. Top: SDS-PAGE of expression tests of each of the four constructs (bottom). M – marker, 1 – 4: Different expression conditions for constructs 1 – 4. Only construct 2 could be expressed in soluble form (in all three conditions). The sequence of the full-length protein, which was used for all experiments involving CgnC in this manuscript, can be found in Supplementary Table 7, the expression conditions in the supplementary online material and the purification protocol in the methods section.

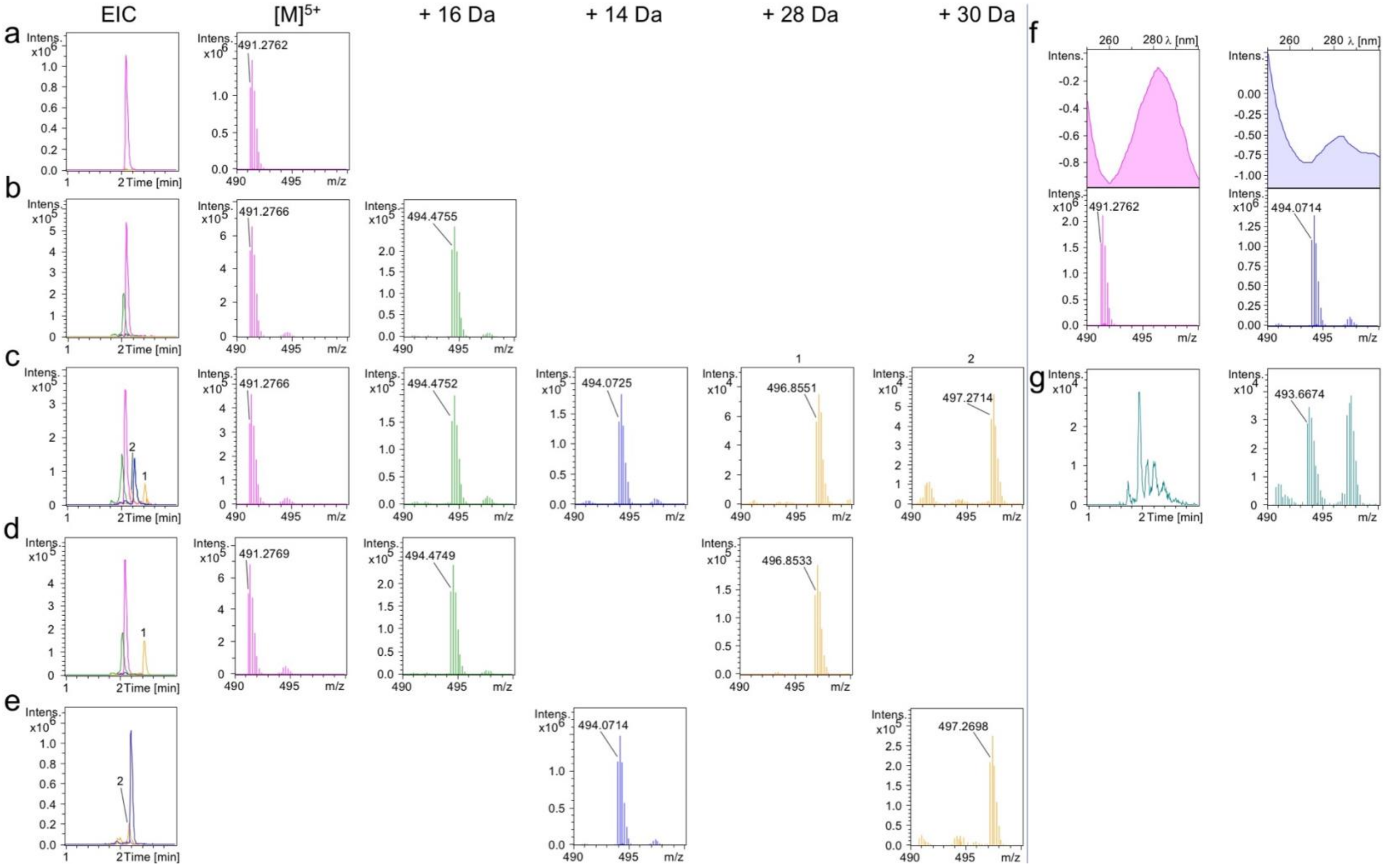

Supplementary Figure 5: a – e: EICs and mass spectra of CgnA reactions with CgnC without co-factors (a), CgnC (b), CgnB/C (c), CgnC/E (d) and CgnB/C/E (e). EICs for CgnA (magenta) and the + 14 Da (blue), + 16 Da (yellow), + 28 Da (orange, 1) and + 30 Da (orange, 2) species are shown in each EIC panel (± 0.01 Da). Errors for the displayed masses are calculated in Supplementary Table 1. f UV spectra associated with reactions shown in a and e. The + 14 Da species loses absorption at 280 nm. g A small peak at a mass shift of + 12 Da was observed in the reaction shown in e. Representative experiments were repeated independently at least three times with similar results.

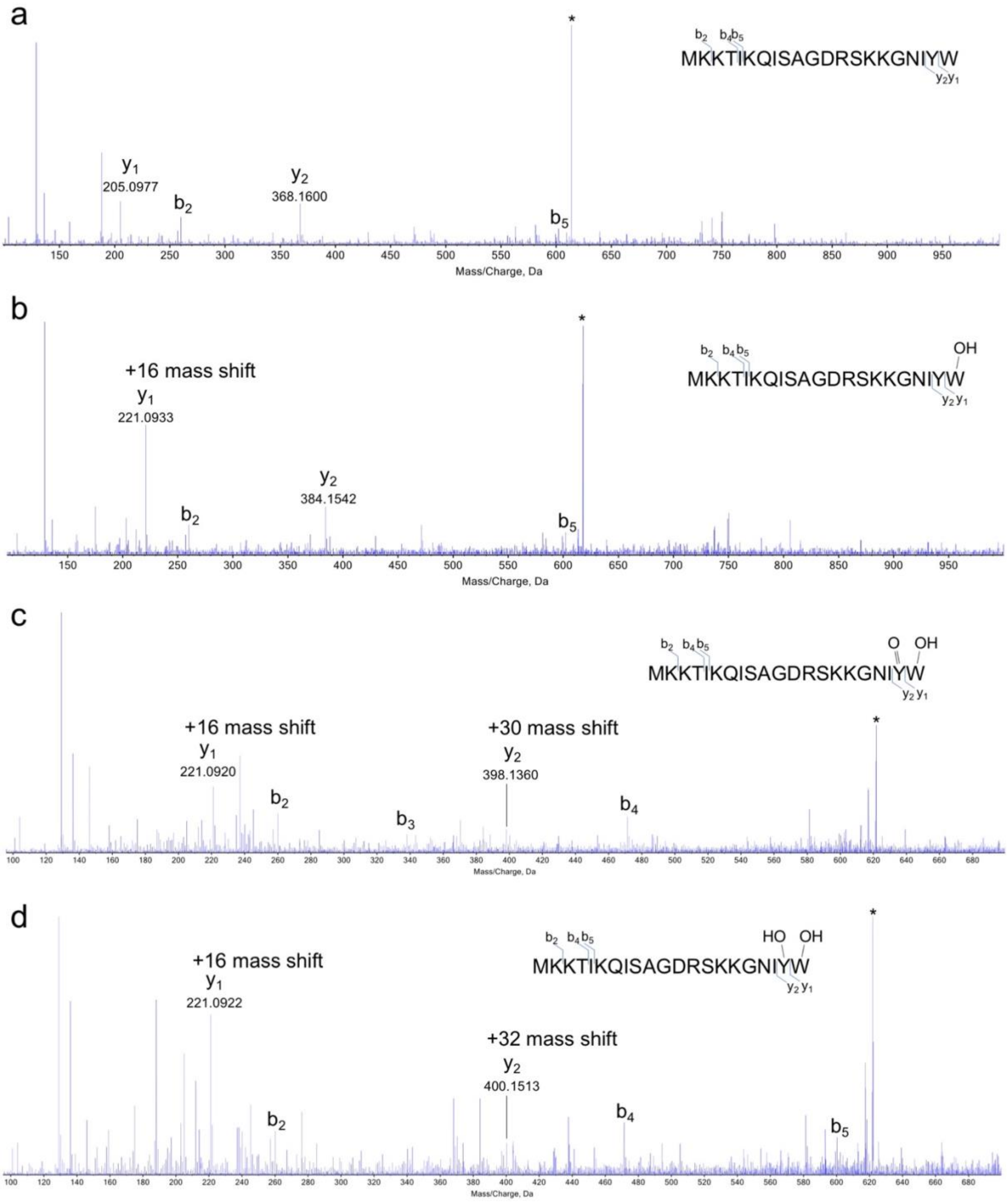

Supplementary Figure 6: MS^2 fragmentation of the 4^+ charge state of a CgnA – CgnC/E reaction. Unmodified CgnA (a, 613.8 m / z (2451.3 Da)), the + 16 Da peak (b, 617.8 m / z (2467.2 Da)), the + 30 Da peak (c, 621.3 m / z (2481.2 Da)) and the + 32 Da peak (d, 621.8 m / z (2483.2 Da)). In the + 16 Da peak, the shift of the y ion fragmentation series by +16 Da from the y_1-site indicates hydroxylation of the Trp. In the + 30 Da peak, the shift of the y ion fragmentation series by +16 Da from the y_1- and + 14 Da from the y_2-site indicates hydroxylation of the Trp, while the keto group is on the Tyr. Finally, in the + 32 Da peak, the shift of the y ion fragmentation series by +16 Da from the y_1- and + 16 Da from the y_2-site indicates hydroxylation of the Trp and Tyr. * indicates unfragmented precursor ion. Representative experiments were repeated independently three times with similar results.

```
mpT        --MDAFKRNLEKLAELAIRVGLNLEKGQEVIATAPIEAVDFVRLLAEKAYREGASLFTVI    58
AmpS        --MTNYKEKLQQYAELLVKVGMNVQPKQPVFIRSSVETLELTHLIVEEAYHCGASDVRVV    58
PepS        MVLPNFKENLEKYAKLLVANGINVQPGHTLALSIDVEQRELAHLIVKEAYALGAHEVIVQ    60
CgnB        -----------------------------------MDVLEYFERLKNR-------ELAFV    18
CgnE        ---------------MGGRRTIGIRSGEGAI----MNASDFYALLRGR-------GMPVV    34
                                               ::  :    :  .        . .

AmpT        Y----GDQELARKRLALAPEEGLDKA-----PAWLYEGMARAFREGAARLAVSGSDPKAL    109
AmpS        Y----SDPTLKRLKFENESVEHFANHE---IKSYDVEARMDYVKRGAANLALISEDPDLM    111
PepS        W----TDDVINREKFLHAPMERLDNV-----PEYKIAEMNYLLENKASRLGVRSSDPGAL    111
CgnB        LDDLQLSDMVTRRGFSVIPFDDFDLAREDHPPAFVLVTRLDYH-----------------    61
CgnE        VDDAEAAAVVSELGFRTVPFEAFDFDSPSEDPALVIVAQMGNV-----------------    77
                     : .  :     : :

AmpT        EGLPPEKVGRAQKANARAYKPALEAITEFVTNWTIVPFAHPGWARAVFPGLPEE-E-AV-    166
AmpS        DGIDSQKLQAFQQQNARAFKGYMESVQKNQFPWVVAAFPSKAWAKRVYPELSVE-EAYI-    169
PepS        NGVDADKLSASAKAMGLAMKPMRIATQSNKVSWTVAAAAGLEWAKKVFPNAASD-EEAV-    169
CgnB        --------------------------GKLMQAWETAKGISSHLSLAKFDTSPKSVEYSLD    95
CgnE        --------------------------DALHGLWERSGTPLMHLALAKFDGGLSRLRAGLA    111
                                            *          :   :       .  :

AmpT        --------------RRLWEAIFQATRADQEDPIAAWEAHNRALHEKVA----YLNARRFH    208
AmpS        --------------KF-IDEVFDIVRIDGNDPVENWRQHIANLSVYAQ----KLQQKNYH    210
PepS        --------------DFLWDQIFKTCRVYEADPVKAWEEHAAILKSKAD----MLNKEQFS    211
CgnB        QLLSMDFAETLKRRGDYYDSVASTNRMEVVTPGAVLTCDFGNEIEIANNDVEMQKGWLYS    155
CgnE        RVLAVDTDAALKRRAEAYEQLFSSASVEIASGEGVLRCHIGDEVEVGNCGDTLEQGFLYS    171
                              : : .               .               :   :

AmpT        ALHFKGPGTDLVVGLAEGHLWQGGAT-ATKGGRLCNPNLPTEEVFTAPHRERVEGVVRAS    267
AmpS        ALHYVSEGTDLTVGLAKNHIWEDATSYVNGKEQAFIANIPTEEVFTAPDRNRVDGYVTNK    270
PepS        ALHYTAPGTDLTLGLPKNHVWESAGA-VNAQGEEFLPNMPTEEVFTAPDFRRADGYVTST    270
CgnB        VAEFFE-TSVINLEADRSSYTLNGDLCFTGL--IYLCNRP-------DLKERASATMDEL    205
CgnE        VAEFLE-ASVVNLEGERSTFWVEGELPFDGF--IHLSNSA-------ALKERWGGMLDEF    221
            . .:    : : :   ..     .             *            .*  . :

AmpT        RPLALGGTLVEGIFARFERGFAVEVRAE--KGEEVLRRLLDTDEGARRLGEVALVPADNP    325
AmpS        LPLSYNGTIIDQFKLMFKDGEIIDFSAE--KGEAVLKDLINTDEGSRRLGEVALVPDDSP    328
PepS        KPLSYNGNIIEGIKVTFKDGQIVDITAE--KGDQVMKDLVFENAGARALGECALVPDPSP    328
CgnB        MRMSTRGR----NVVSFVDNQIVRMELGGVDMTATLRELIVGKEREGSSTEFAMGCVEYP    261
CgnE        MRRSREGA----NLVRFADNVIDRLVVGGVDVTSALAGLSQGEERGMAATEFGLGCADAE    277
               :  *         *  .    .     .   .:  *   .       * .:

AmpT        IAKTGLVFFDTLFDENAASHIAFGQAYQENLEGRP--SGEAFRKRGGNESLVHVDWMIGS    383
AmpS        ISNRNTIFYNTLFDENAACHLAIGSAYAFNIQGGTEMTVEEKIASGLNDSNVHVDFMIGS    388
PepS        ISQSGITFFNTLFDDNASNHLAIGAAYATSVVDGAEMSEEELEAAGLNRSDVHVDFMIGS    388
CgnB        LAQDWT--INSVMNEG---------------------SHGIHVGVGMGKEIPHMDFIAKG    298
CgnE        AAEPFG--VNSLLHKS---------------------AGGAYIGIGKGLRIPHIDFIARG    314
             ::      ::::...                     :       * .    *:*::  .

AmpT        EEMDVDGLYEDGTRTPLMRRGRWVV--     408
AmpS        SDLTIYGIFEDGSKELVFENGNWASTF     415
PepS        NQMDIDGIREDGTRVPLFRNGNWAN--     413
CgnB        AELRIAESSDA----------------     309
CgnE        ATIRFIPAAEG----------------     325
              : .    :
```

```
Percent Identity Matrix - created by Clustal2.1
     1: AmpT        100.00   37.84   40.69   14.49   16.44
     2: AmpS         37.84  100.00   47.32   16.85   14.92
     3: PepS         40.69   47.32  100.00   16.25   15.02
     4: CgnB         14.49   16.85   16.25  100.00   37.54
     5: CgnE         16.44   14.92   15.02   37.54  100.00
```

Supplementary Figure 7: Sequence alignment of CgnB and CgnE with HHPred hits AmpT, AmpS and PepS. Sequence alignments and percent identity matrices were generated with Clustal Omega.[2]

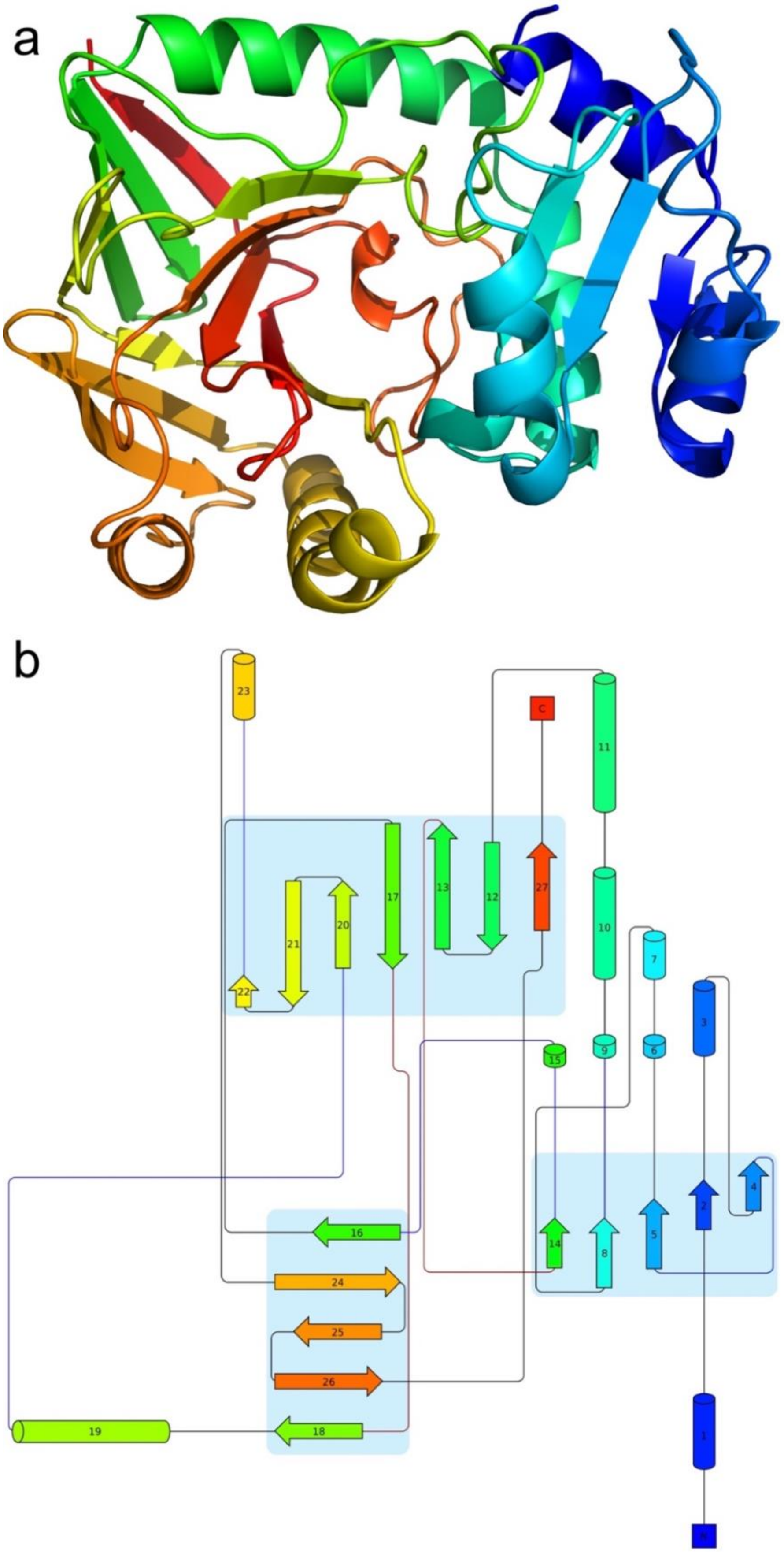

Supplementary Figure 8: Crystal structure of CgnE. a Cartoon representation of the CgnE crystal structure. b Secondary structure diagram of CgnE generated with Pro-origami.[3] β-strands that form sheets are grouped and highlighted in light blue. The rainbow color scheme is used in both panels.

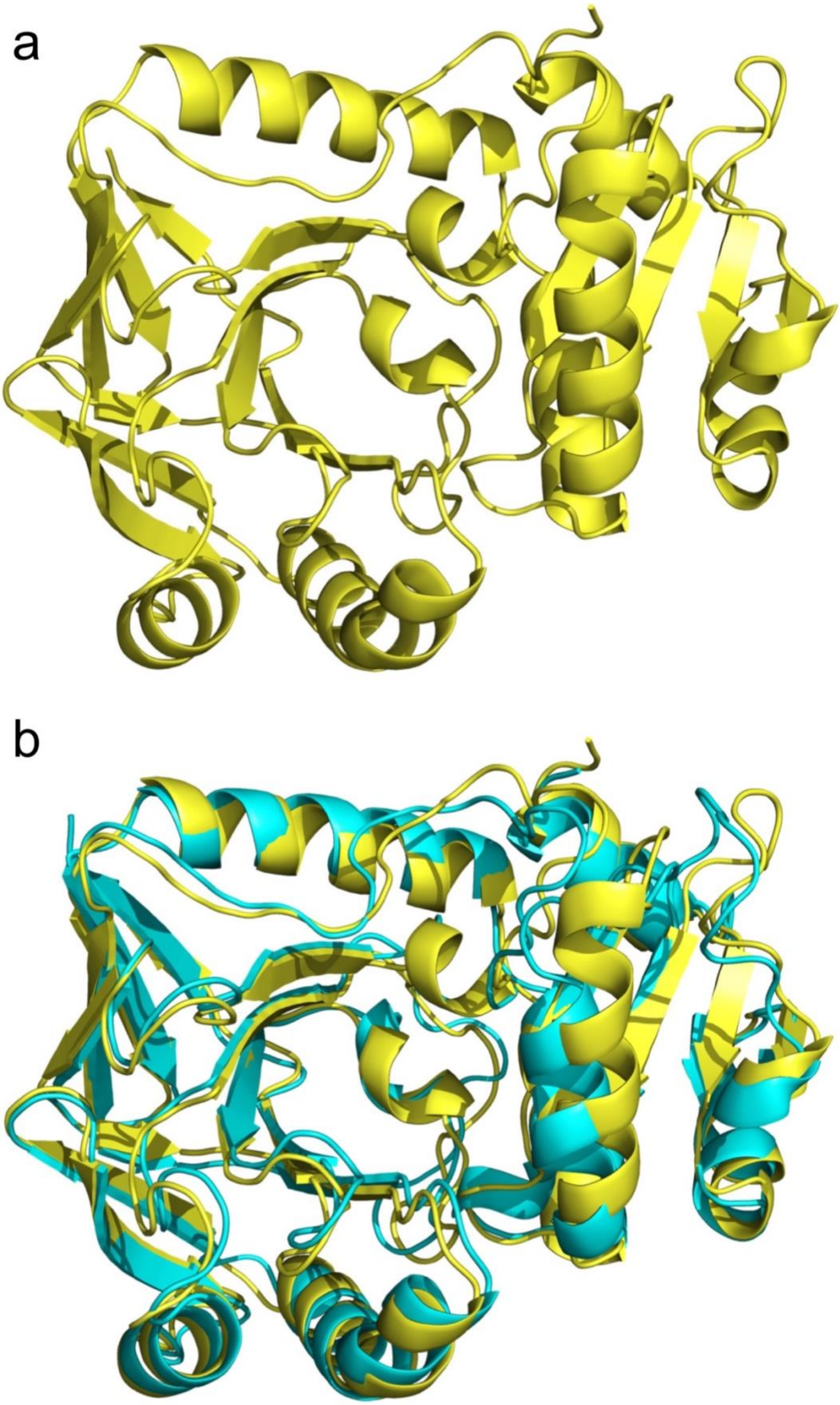

Supplementary Figure 9: The CgnB crystal structure and its comparison to CgnE. a Cartoon representation of the CgnB crystal structure. b Superposition of the crystal structures of CgnB (yellow) and CgnE (cyan).

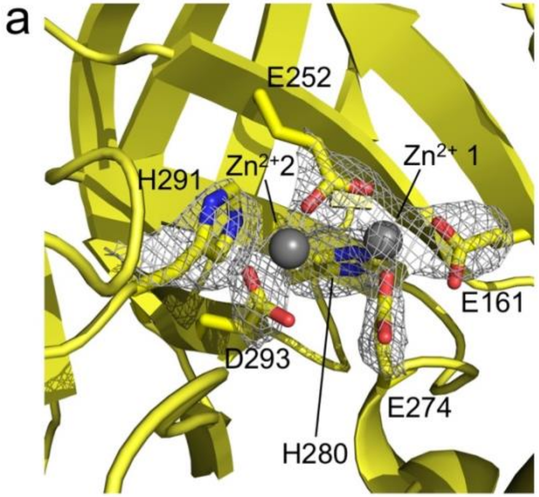

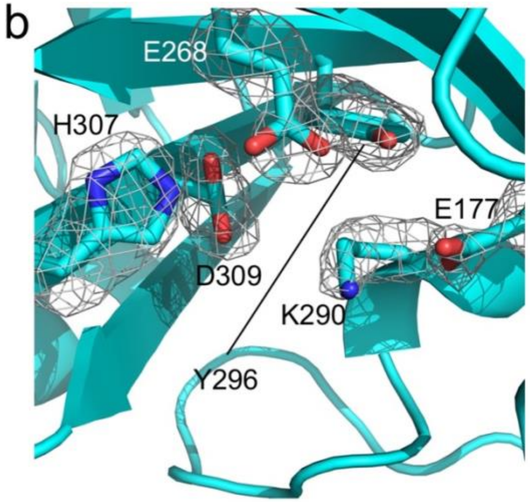

c

	ppb	Std%
	Zn concentration	
CgnB	43.0	2.9
CgnB dialyzed	21.6	1.5
Buffer	21.2	1.8
	Fe concentration	
CgnE	9.8	10.4
CgnE + iron	19.7	9.7
Buffer	19.2	7.9

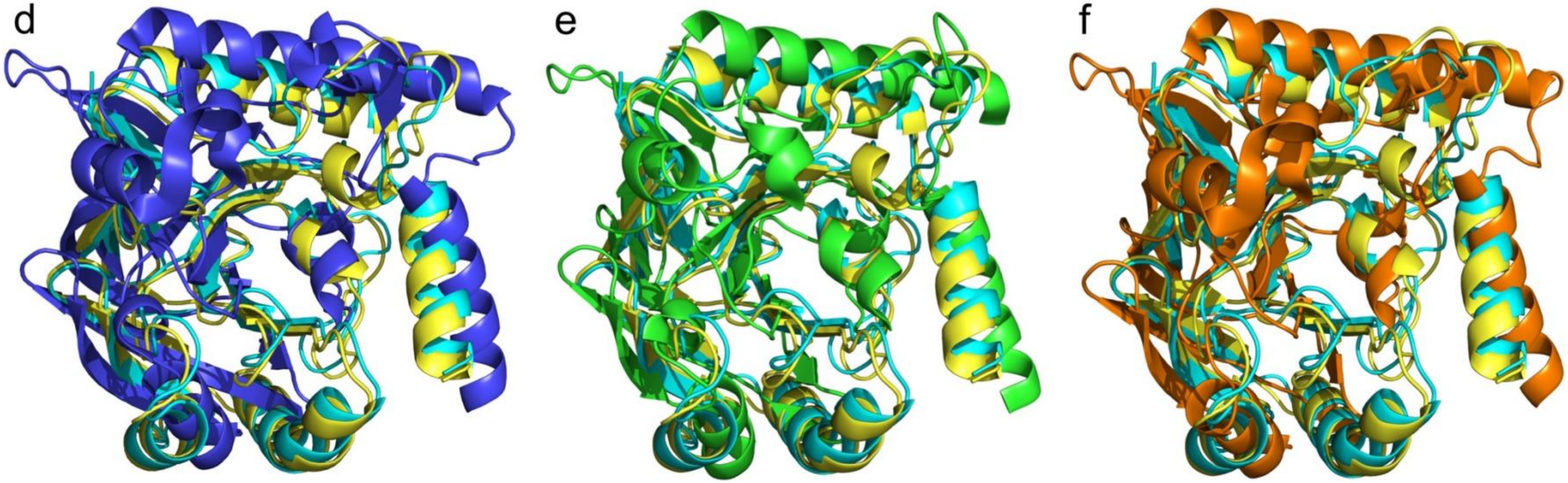

Supplementary Figure 10: Analysis of CgnB and CgnE. a and b Polder maps contoured at 3 σ of active-site residues and metal ions bound at the active-site of CgnB (a, yellow) and CgnE (b, cyan). c ICP-MS data confirming zinc bound to CgnB and CgnE's inability to bind Fe. d – f Superposition of CgnB (yellow) and CgnE (cyan) with Aminopeptidase AMPS (d, blue, PDB ID 1zjc), Aminopeptidase T (e, green, PDB ID 2ayi) and PepS (f, PDB ID 4ics). The N-termini (compare Supplementary Figure 11) are omitted for clarity.

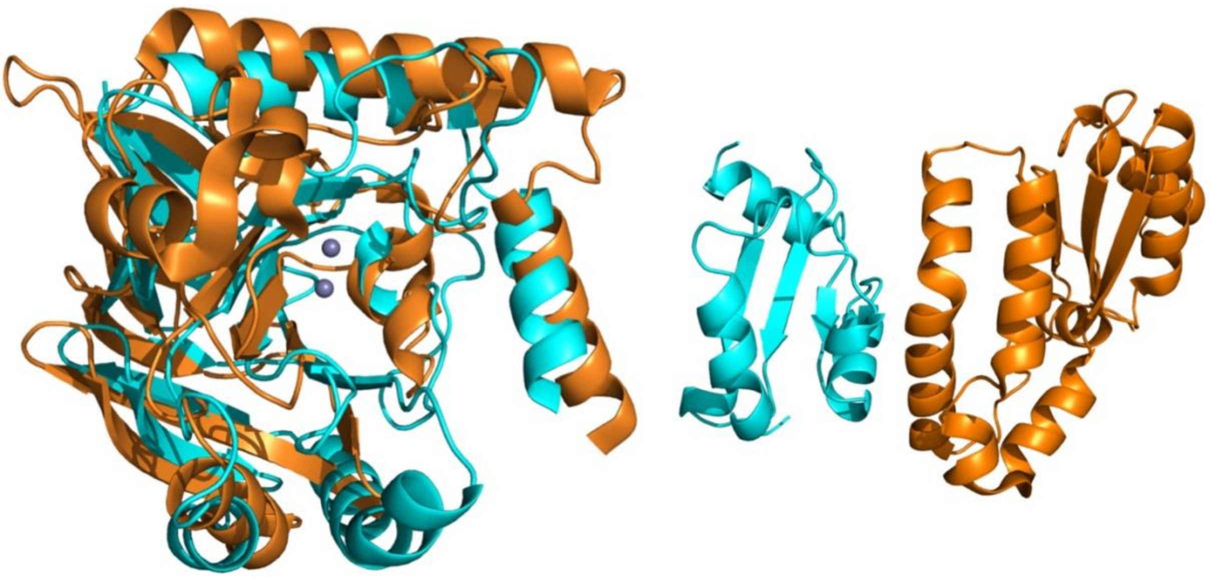

Supplementary Figure 11: Comparison of CgnE with the closest structural homologs. Superposition of the C-terminal subdomain of CgnE (cyan) with aminopeptidase PepS (orange, PDB ID 4ics). The majority of residues align well, but the N-terminal subdomains (shown on the right) are structurally unrelated. Dimerization of PepS and its homologs is facilitated by the N-terminus.

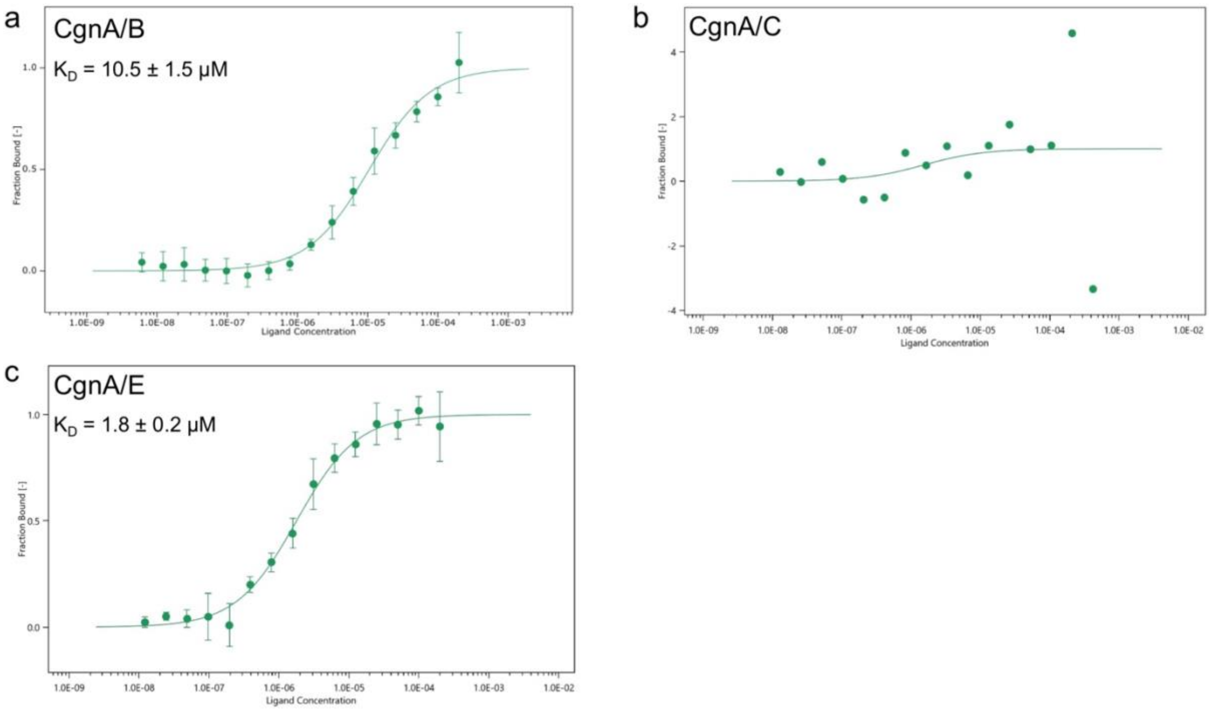

Supplementary Figure 12: a – c Analysis of the affinities of CgnB (a), CgnC (b) and CgnE (c) for CgnA by MST. Each curve represents three independent samples, data points represent the mean and the error bars represent standard deviations.

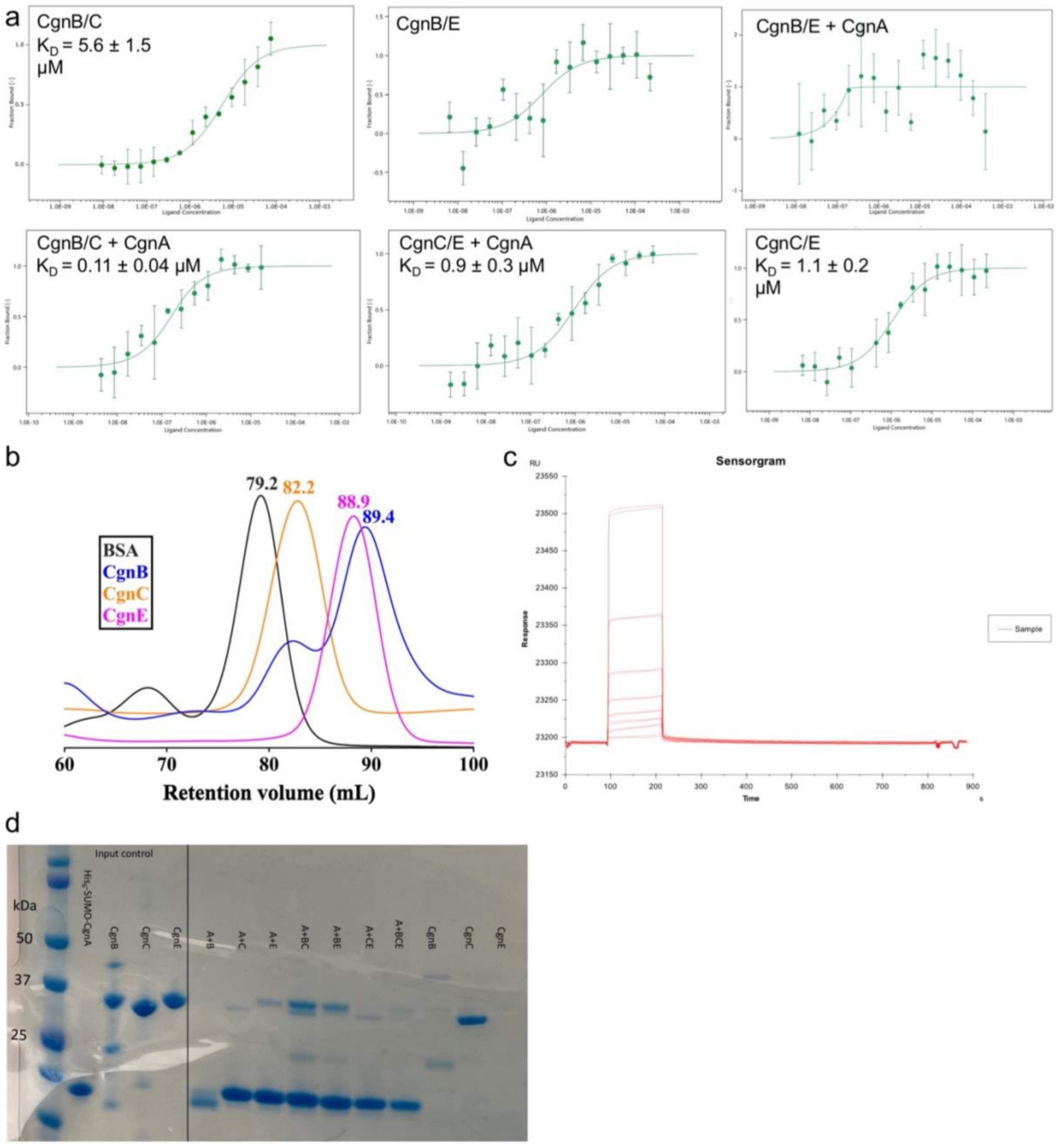

Supplementary Figure 13: Analysis of protein complexes using SEC and MST. a MST measurements to determine K_Ds of the protein pairs given. X/Y – Affinity of fluorescently labeled X to Y (titrated). + Z: Z was supplied at a constant, high concentration. Each curve represents three independent samples, data points represent the mean and the error bars represent standard deviations. b SEC analysis of CgnB (blue), CgnC (orange), CgnE (pink) and bovine serum albumin (black). CgnC elutes earlier than CgnB and CgnE, closer to bovine serum albumin, which is roughly the size of a CgnC dimer. c SPR experiment showing that CgnC binds non-specifically to the sensor chip in a concentration-dependent manner. d Pull-down experiments of CgnB, CgnC and CgnE with His_6-SUMO-CgnA. Left: Input controls. Right: Pull-downs of CgnB, CgnC, CgnE, or combinations thereof using His_6-SUMO-CgnA bound to Ni^{2+}-beads. CgnC binds to the resin non-specifically.

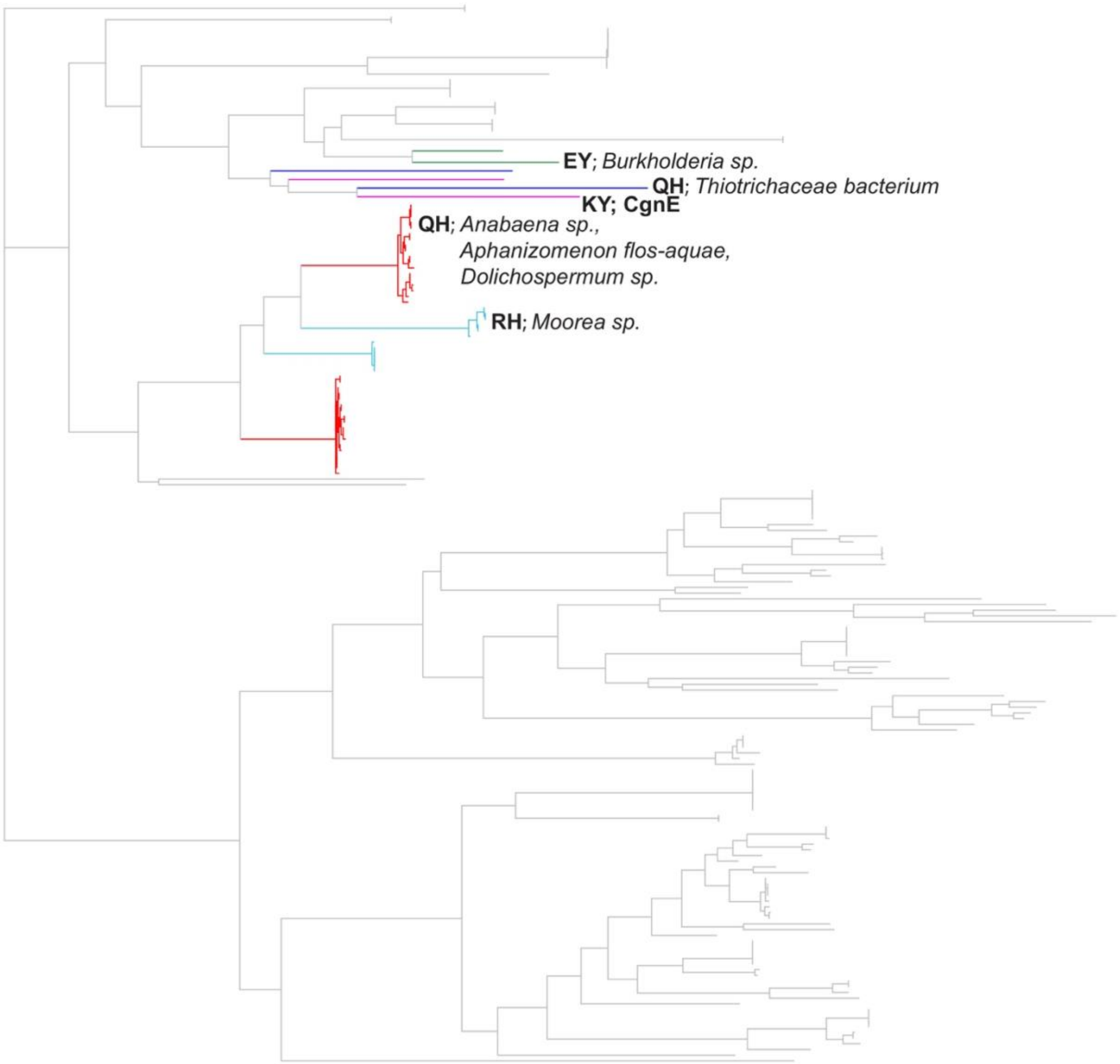

Supplementary Figure 14: Phylogenetic analysis of the distribution of CgnB/CgnE in published bacterial genomes. Instances of two copies are colored and the mutations disrupting metal binding at the active-site are given for each color using one-letter code, which describes the observed substation pattern (E.g. RH - Glu274Arg and His280His, CgnB numbering). CgnE is labeled. A phylogenetic tree for the colored section with accession codes and bootstrap values is provided as a separate supplementary file "Supplementary_Data_1.pdf".

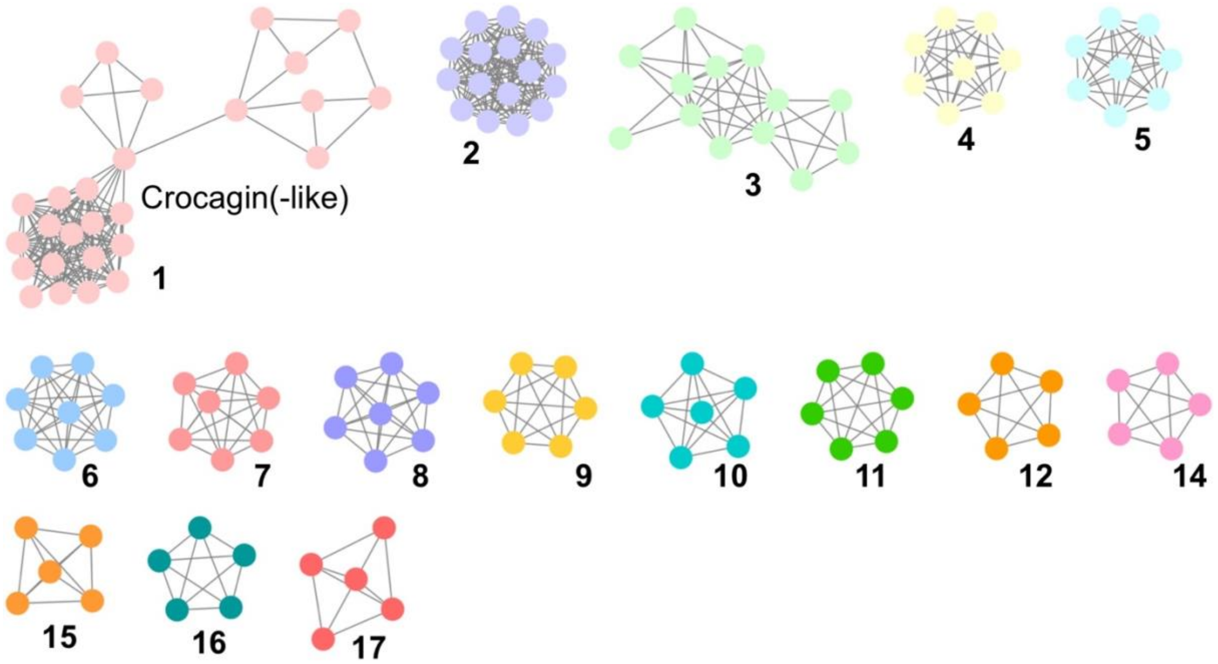

Supplementary Figure 15: Sequence similarity network of putative precursor peptides that were identified using RiPPer[4] using the default settings and the proteins CgnB/E. The crocagin-like biosynthetic gene clusters containing two copies are labeled. The other clusters are numbered. Details, such as accession codes and precursor peptide sequences, can be found in the supplementary Excel file "Supplementary_Table_1.xlsx".

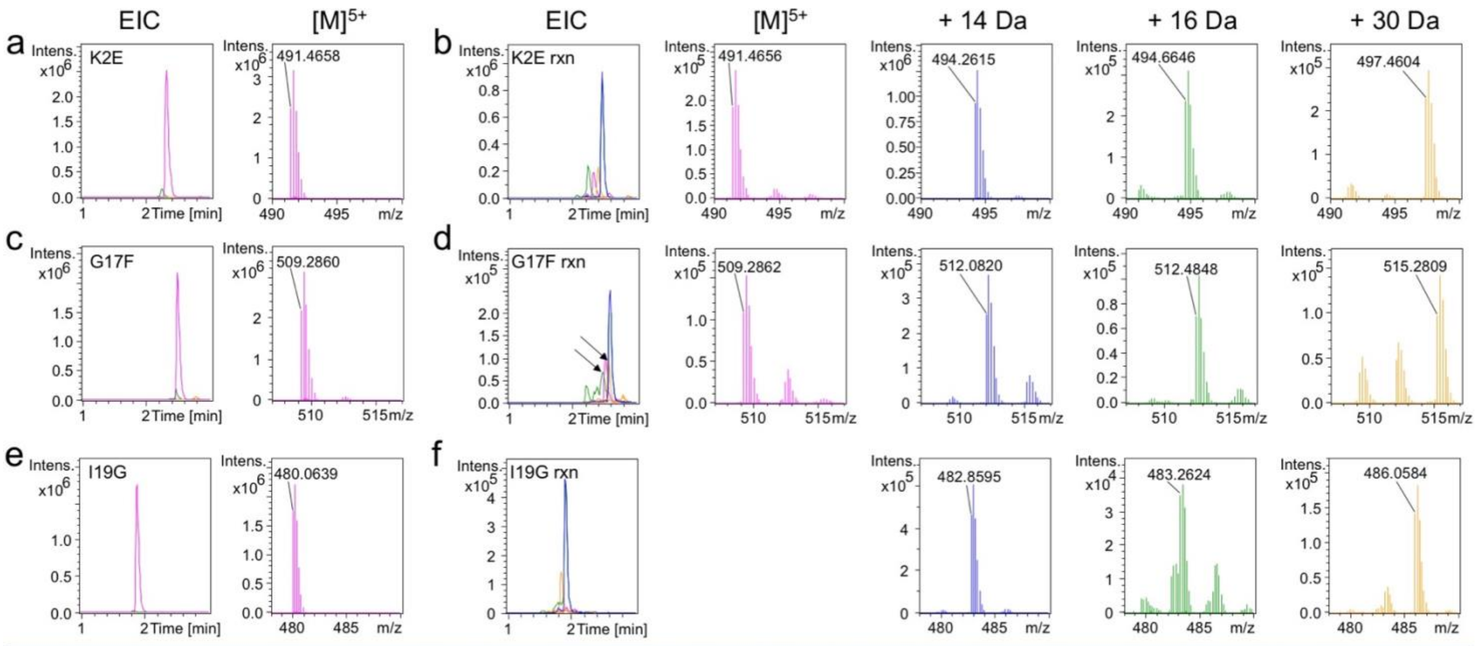

g

Accession code / protein ID	Precursor peptide sequence	% positive charges
CgnA	MKKTIKQISAGDRSKKGN**IYW**	**33**
BJCF01000037	MKKAKQVSKANRKARQN**AYW**	**41**
JAAHIG010000012	MKKEIKKETQHKETASKKRKLEQVDKNTRSKLGN**GYW**	**35**
KE384588	MNAKKTQKRKLEQVNKETRSNLGN**AYW**	**29**
FTPM01000001	MNQAQQVKVRPVSPEERRIAQN**YYW**	**18**
LJOW01000058	MSAKKTQKRKLEQVNKETRSNLGN**VYW**	**29**
CP012159	MTMKKTIKQISAGDRSKKGN**IYW**	**30**

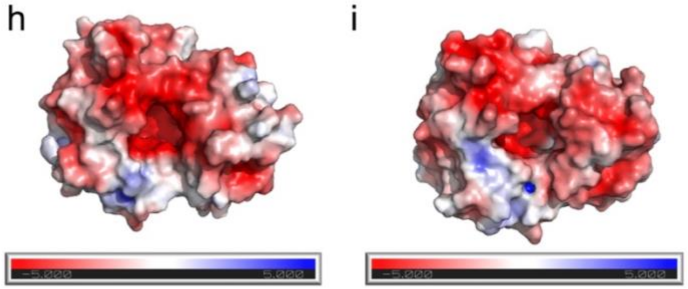

Supplementary Figure 16: Reaction of CgnA point mutants and electrostatics of the CgnA – CgnB/E interaction. a – f EICs and mass spectra of CgnA mutants and reactions (rxn) of these mutants with CgnB/C/E. EICs for CgnA (magenta) and the + 14 Da (blue), + 16 Da (green), and + 30 Da (orange) are shown in each EIC panel. Full conversion is observed for the CgnA I19G mutant (thus the missing $[M]^{5+}$ panel in f). All EICs were generated using a ± 0.01 Da threshold. Errors for all displayed masses are calculated in Supplementary Table 1. Representative experiments were repeated independently at least three times with similar results. g Representative precursor peptides from all unique groups of leader peptides shown in Supplementary Table 4. Accession codes for the selected precursor peptides are given, positively charged residues highlighted and the percentage of positively charged residues with the leader peptide calculated. Core peptide sequences are bold. With the exception of the precursor peptide found in *Burkholderia sp.* (FTPM01000001), the percentage of positively charged amino acids is ≥ 29%. h and i Electrostatic surface potentials of CgnB (h) and CgnE (i).

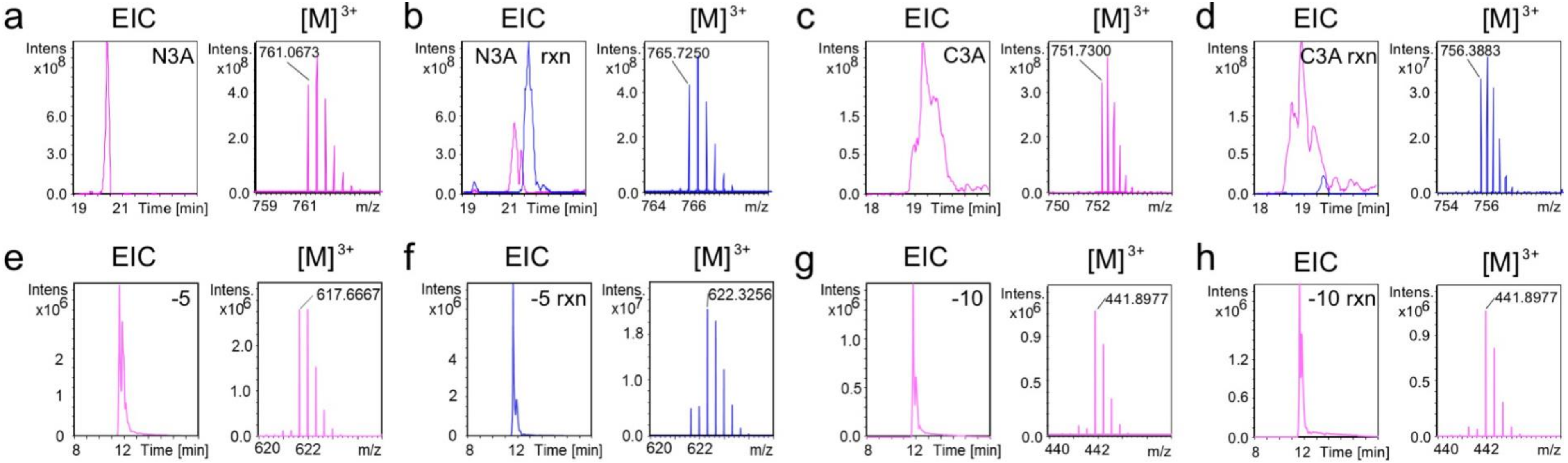

Supplementary Figure 17: EICs and mass spectra of CgnB/C/E reactions with truncated or mutated CgnA. Unreacted substrate is shown in pink, the + 14 Da product in blue. a and b CgnA^{N3A} control (a) and reaction (rxn, b). The reaction was impaired compared to wild-type substrate. c and d CgnA^{C3A} control (c) and reaction (rxn, d). While product can be detected, most of the starting material remains unreacted. e – h EICs and mass spectra of truncated CgnAs (5 and 10 amino acids removed from the N-terminus). While the -5 truncation still reacts as the native substrate, no product can be detected for the reaction with -10. Errors for all displayed masses are calculated in Supplementary Table 1 and sequences given in Supplementary Table 7. All EICs were generated using a ± 0.01 Da threshold. Representative experiments were repeated independently at least three times with similar results.

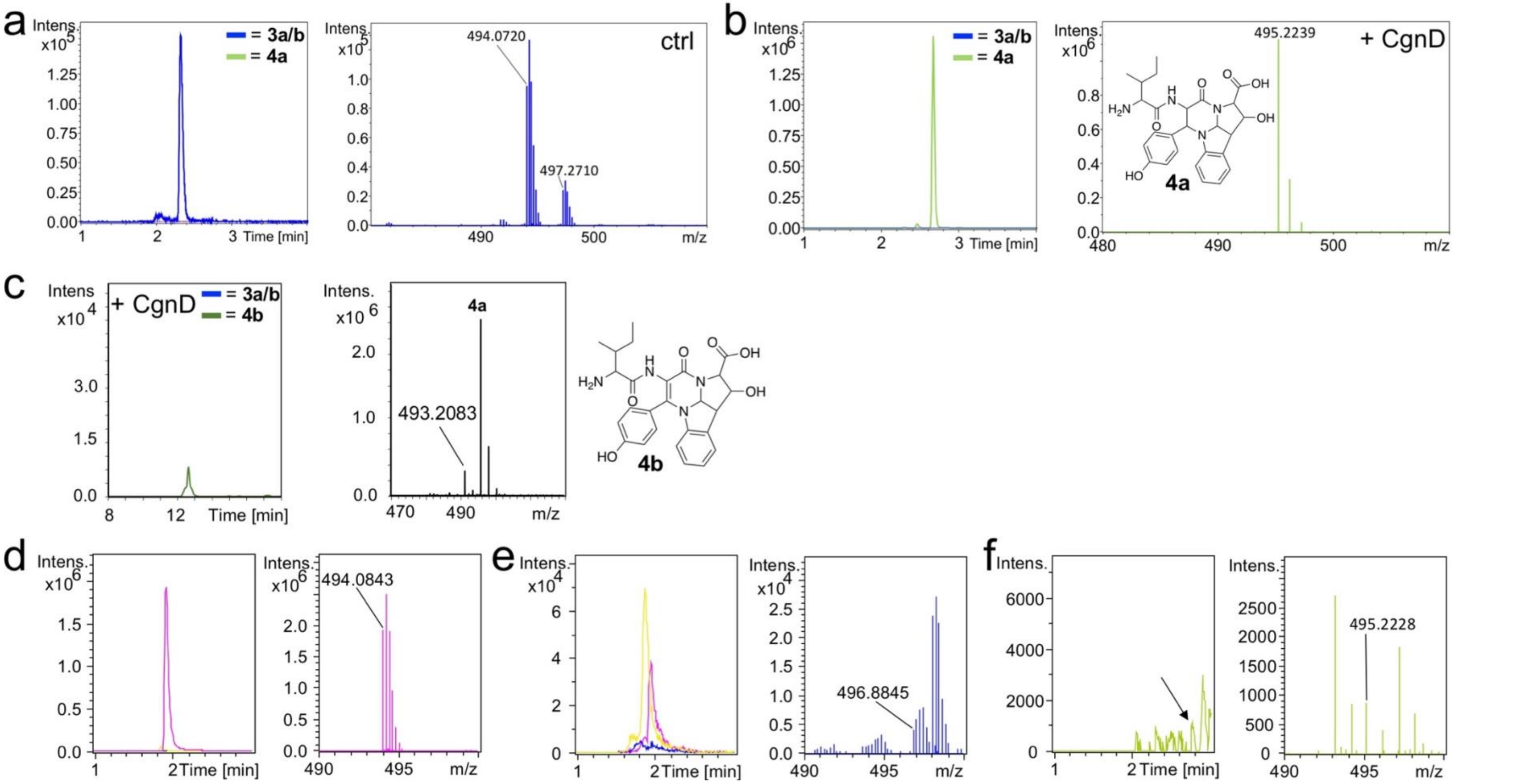

Supplementary Figure 18: Production of the crocagin core scaffold. a – c Production of the crocagin A and B core scaffolds using CgnD. d – f Production of the crocagin A core scaffold **4a** using CgnA^{N18K} as a substrate and trypsin as the protease for leader peptide removal. d EIC and mass spectrum of CgnA^{N18K}. e EIC and mass spectrum of CgnA^{N18K} after incubation with CgnB/C/E. Very little product (blue) is formed. The majority corresponds to a species displaying a mass shift of + 16 Da (yellow). f EIC and mass spectrum of the reaction from e after incubation with Trypsin. Very little **4a** is formed. Arrow indicates peak containing **4a**. All EICs were generated using the expected mass ± 0.01 Da. Mass errors can be found in Supplementary Table 1. Representative experiments were repeated independently at least three times with similar results.

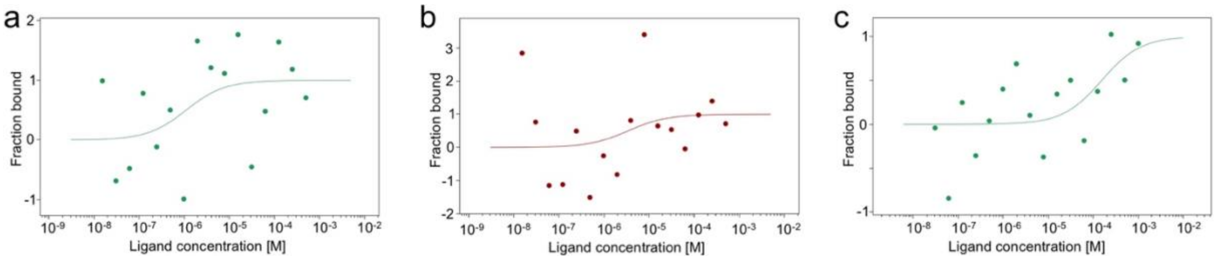

Supplementary Figure 19: MST analysis of the binding of CgnD to **3a**/**b** (a), CgnA (b) and the CgnA leader peptide (c). Affinities were too weak to be calculated. Representative experiments were repeated independently three times with similar results.

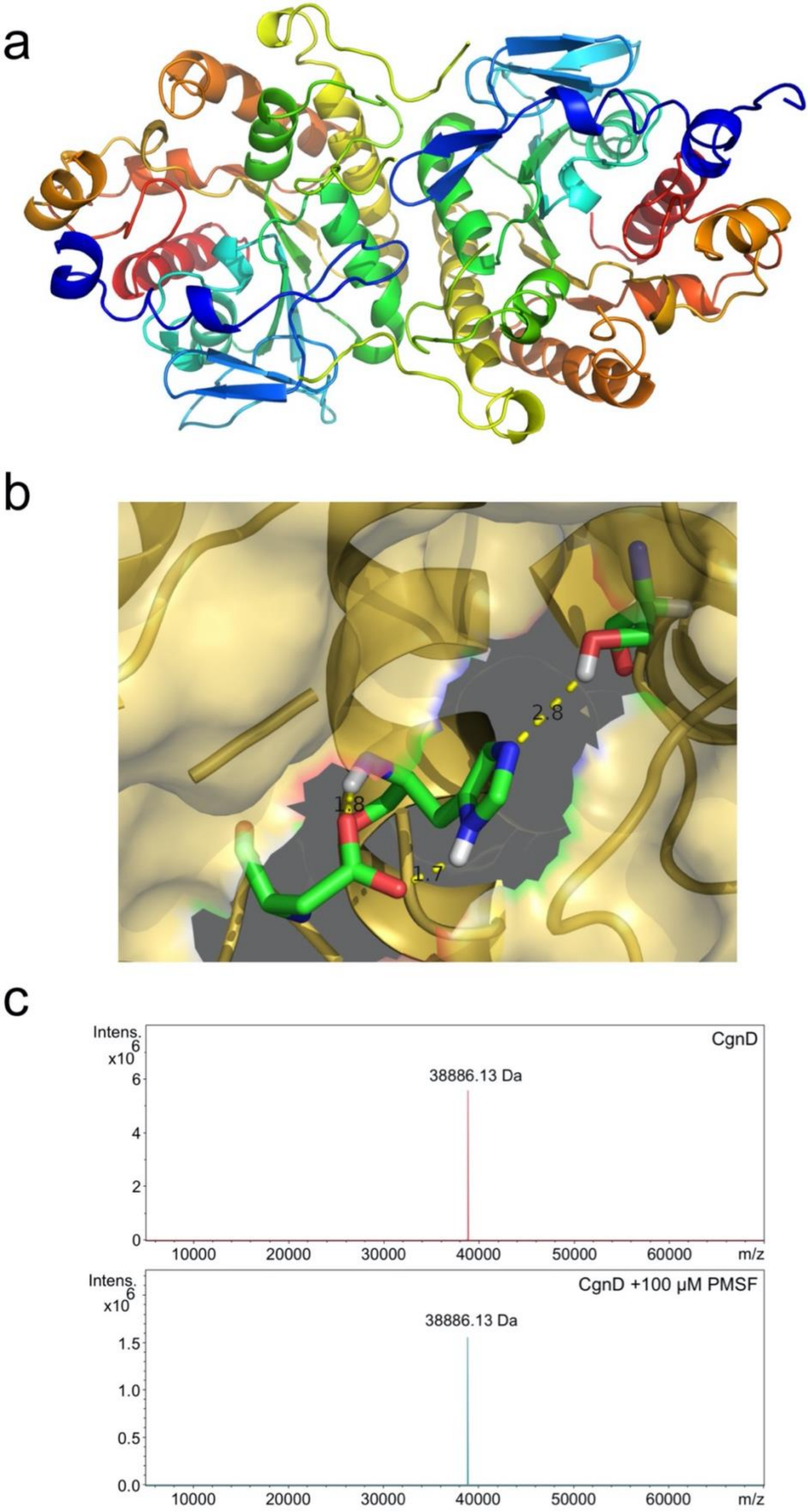

Supplementary Figure 20: Crystal structure and analysis of CgnD. a Cartoon representation of the CgnD dimer using the rainbow color scheme. b Close-up of the catalytic triad of CgnD. Distances are given in Å. c Addition of PMSF does not lead to a mass shift of CgnD. (Calculated mass 38,886.12 Da). Representative experiments were repeated independently three times with similar results.

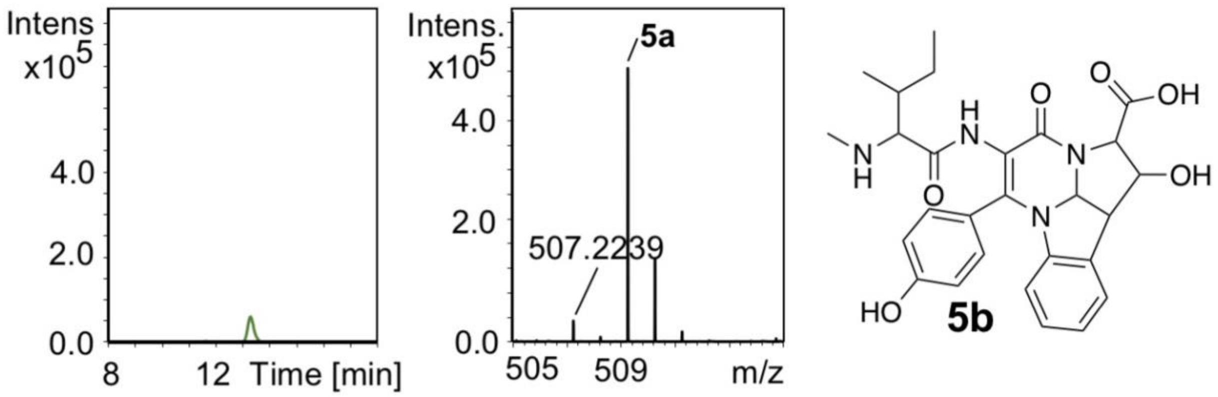

Supplementary Figure 21: Detection of **5b**. When the product of CgnD reactions with **3a/b** was incubated with CgnL a minor product peak for 5b could be observed. The EIC was generated using the expected mass ± 0.01 Da. Mass error can be found in Supplementary Table 1. Representative experiments were repeated independently three times with similar results.

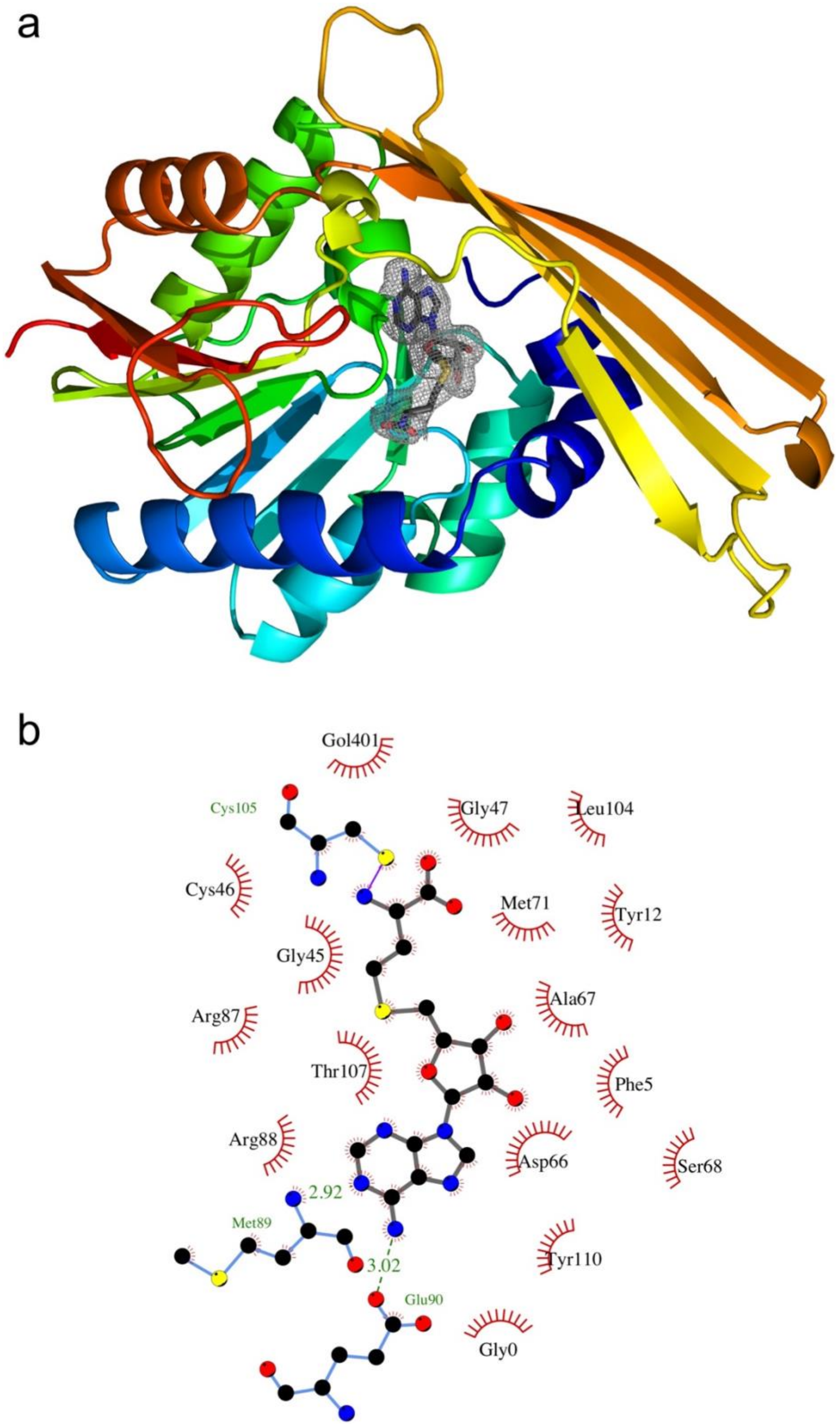

Supplementary Figure 22: Crystal structure of CgnL and binding of SAH. a Cartoon representation of the crystal structure of CgnL using the rainbow color scheme. The ligand SAH is shown as grey sticks with a POLDER map calculated at 3 σ shown as an isomesh. b LigPlus[5] diagram showing the interactions of SAH (grey bonds) with CgnL (blue bonds). Hydrogen bonds and salt bridges are indicated by green, dashed lines and distances given (in Å). Hydrophobic interactions are represented as red spoked arcs.

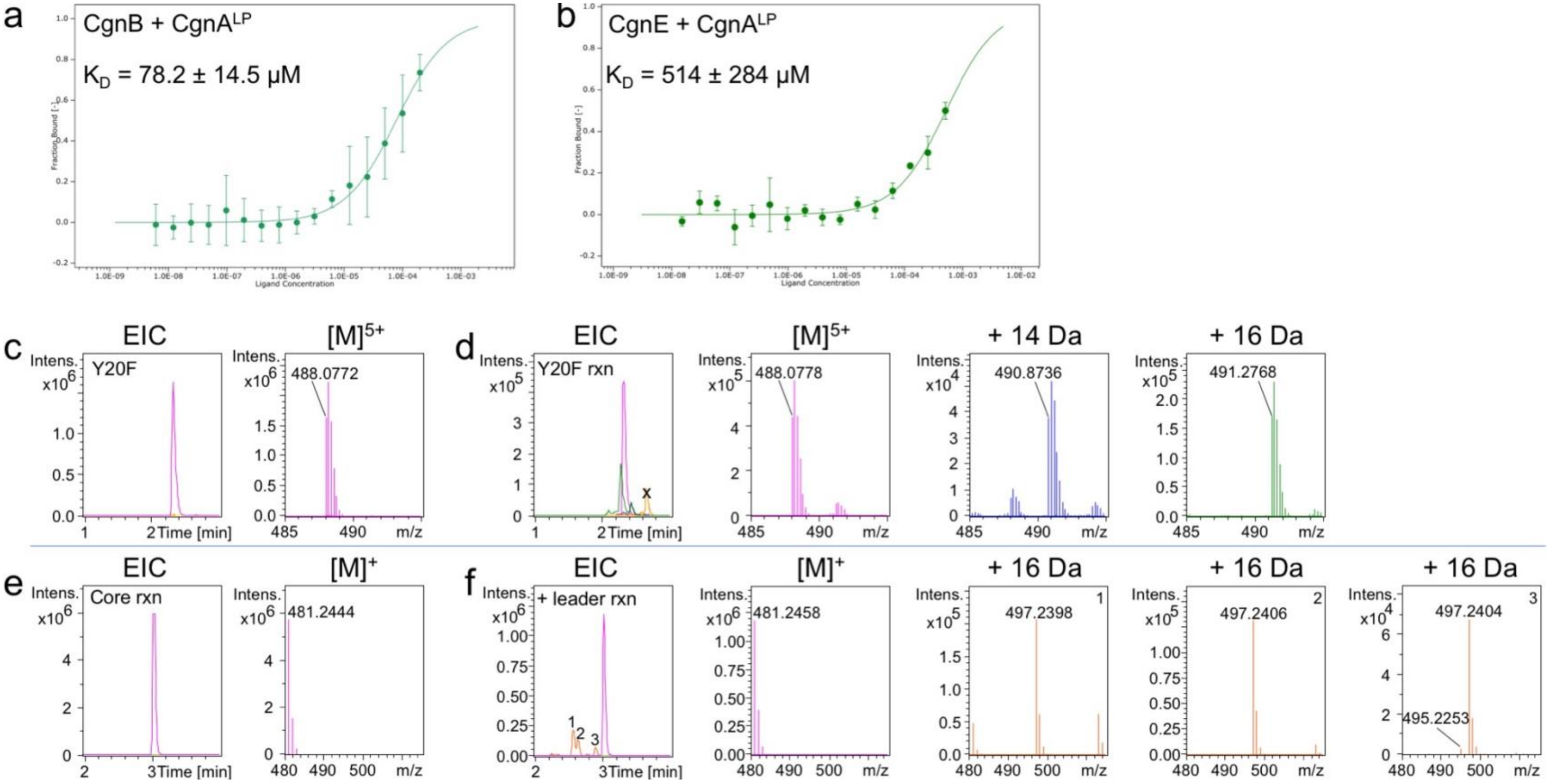

Supplementary Figure 23: Affinity of CgnB and CgnE to the leader peptide and processing of CgnA point mutation / core peptide. a and b Affinities of CgnB and CgnE for the CgnA leader peptide ($CgnA^{LP}$). Each curve represents three independent samples, data points represent the mean and the error bars represent standard deviations. c and d EICs and mass spectra of CgnA point mutant Tyr20Phe. It is unclear if the small + 14 Da peak represents true product, or oxidation of the hydroxylated + 16 Da peak to ketone (+ 14 Da). “x” indicates an unrelated peak with a similar mass. e and f Reaction of the core peptide (magenta) without (e) or with (f) leader peptide added. When leader peptide is added, three distinct species of a mass corresponding to the hydroxylated core peptide can be observed (numbered 1 – 3), but it was too little material for further analysis. It is unclear why three peaks with (slightly) different retention times are observed, but two could represent hydroxylation at the Tyr Cβ and Trp Cβ. Only putative traces of the desired product (+ 14 Da) can be found in panel 3 of f. All EICs were generated using a ± 0.01 Da threshold. Errors for all displayed masses are calculated in Supplementary Table 1 and sequences given in Supplementary Table 7. Representative experiments were repeated independently at least three times with similar results.

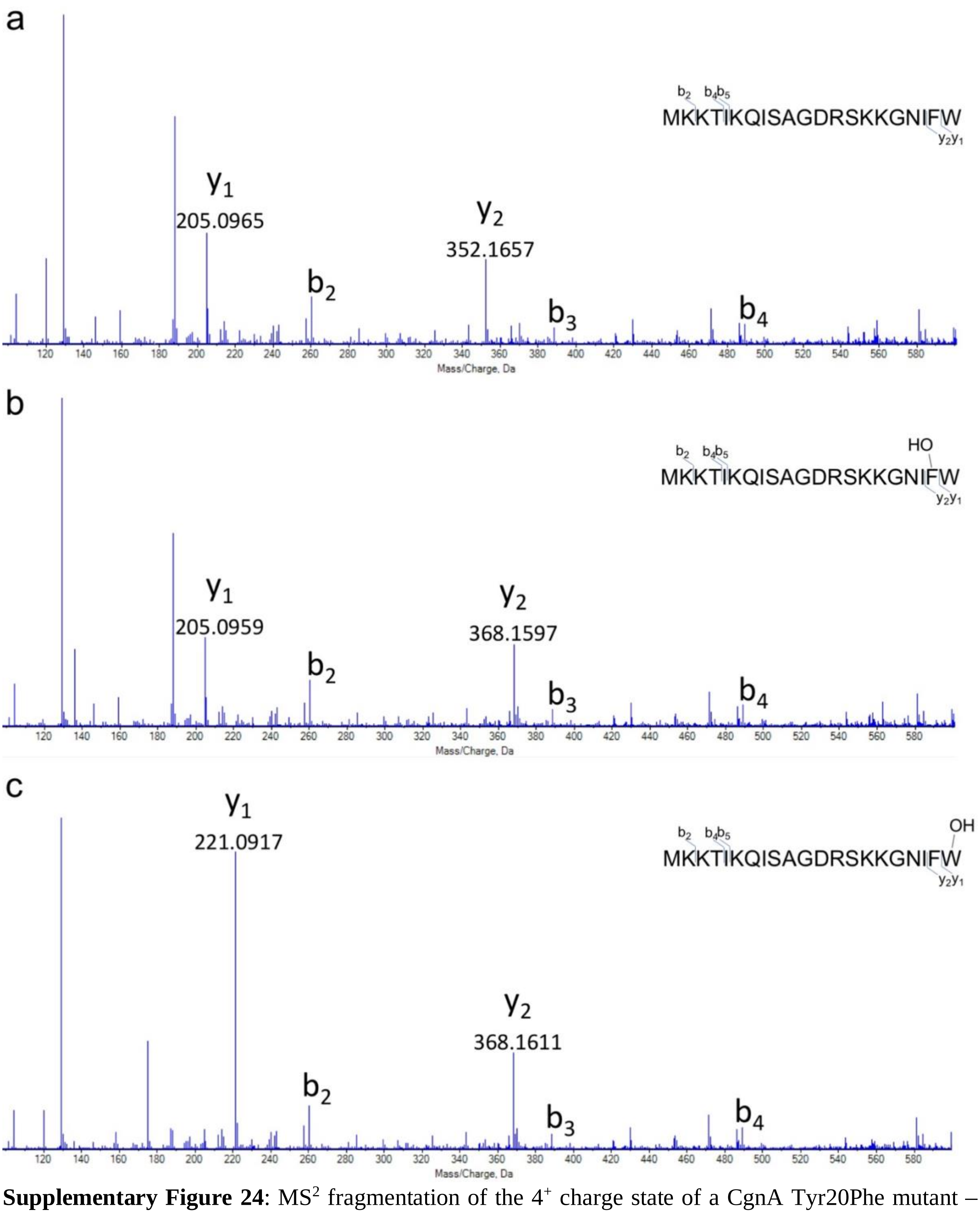

Supplementary Figure 24: MS^2 fragmentation of the 4$^+$ charge state of a CgnA Tyr20Phe mutant – CgnB/C/E reaction. Unmodified CgnAY20F (a, 609.8 m / z (2435.3 Da)) and the + 16 Da peak (b and c, 613.8 m / z (2451.2 Da)). In the + 16 Da peak, the shift of the y ion fragmentation series can be placed on either the y_1- or the y_2-site. In contrast to the wild-type substrate, where the shift can be placed on the Trp, the Tyr to Phe mutation appears to result in a mixture. Representative experiments were repeated independently three times with similar results.

Methods

Cloning of Cgn constructs used in this study

For CgnB and CgnD, only the original annotation of the open reading frame for these proteins was utilized for cloning.[1] Four glycine residues were added to the annotated start codon of CgnL to facilitate TEV cleavage during purification. In contrast, several expression constructs were designed based on genomic information on an alternative N-terminus for CgnC (Supplementary Figure 4) and CgnE (Supplementary Table 8). The corresponding primers for every construct were ordered from Sigma-Aldrich and the different gene constructs amplified by PCR with a standard Phusion polymerase protocol using adjusted annealing temperatures. DNA amplificates of *cgnC*, *cgnD*, *cgnE* and *cgnL* were subsequently digested with *NcoI* and *HindIII* in buffer R at 37 °C for 4 h and ligated into the pSUMO vector (the plasmid was a gift from Dr. David Owen, DIAMOND[6]) digested with the same enzymes and treated with alkaline phosphatase using a standard T4 DNA ligase protocol. The PCR product of *cgnB* was digested with *BamHI* and *HindIII* as described above and ligated into the pSUMO vector digested with the same enzymes and prepared as above. Ligations were transformed into chemically competent *E. coli* HS996 with a standard heat shock protocol, plated on LB-Agar with added 50 µg mL^{-1} Kanamycin and grown at 37 °C for 16 h. Single *E. coli* colonies were grown in 10 mL LB-medium with 50 µg mL^{-1} Kanamycin for 16 h at 37 °C and 200 rpm. 4 mL of grown cultures were centrifuged for 10 min at 3,000 xg and 4 °C and the cell pellet was used for a plasmid preparation by alkaline lysis. Extracted plasmids were digested with the respective restriction enzymes used for cloning using the same procedure as above and analyzed by agarose gel electrophoresis. Plasmids, which were found to carry the correct insert, were sent for sequencing by LGC genomics (Berlin) and subsequently transformed into chemically competent *E. coli* Lemo21(DE3) cells for pSUMO-CgnC variants. pSUMO-CgnD and pSUMO-CgnE variants were transformed into *E. coli* Rosetta2(DE3) cells whereas pSUMO-CgnB and pSUMO-CgnL were transformed into *E. coli* BL21(DE3) cells.

Small scale protein expression tests of Cgn constructs

Small scale protein expression testing was carried out by transferring a freshly transformed *E. coli* BL21(DE3), *E. coli* Rosetta2(DE3) or *E. coli* Lemo21(DE3) colony with the respective pSUMO-Cgn construct into 10 mL LB-media supplemented with 50 µg mL^{-1} Kanamycin and, where appropriate, 34 µg mL^{-1} Chloramphenicol. Cultures were grown for 16 h at 37 °C and 200 rpm and used to subsequently inoculate 50 mL LB-medium supplemented with the respective antibiotics at a 1:100 ratio. For small scale expression testing, a variety of parameters including temperature, IPTG concentration and point of induction were analyzed in different combinations to increase the yield of soluble protein in the Ni^{2+} pull-down assay. All cultures were then centrifuged at 3,000 xg and 4 °C for 10 min to harvest the wet cell pellet. The supernatant was discarded, and the cell pellet was frozen at - 80 °C until further use.

Magnetic Ni^{2+}-beads were utilized to separate His_6-tagged proteins from the lysate of disrupted *E. coli* cells and to test their solubilities in different lysis buffers in a medium throughput approach. *E. coli* cell

pellets were resuspended in 500 µL of a respective lysis buffer and subsequently sonicated on ice with an amplitude of 75 % using 2, 15 s burst cycles with a 30 s recovery time. Afterwards, the lysate was centrifuged at 21,000 xg and 4 °C for 10 minutes before being used in the Ni^{2+} pull-down assay on a KingFisher mL purification system (Thermo Scientific). The lysate was mixed with 50 µL of MagneHis Ni particles (Promega) before being washed twice with 500 µL lysis buffer. The His_6-tagged proteins were eluted from the magnetic Ni^{2+} particles with 50 µL of elution buffer supplemented with 250 mM Imidazole. Eluted fractions were run on an SDS-PAGE to compare yield and sizes of recombinantly expressed proteins with the Precision Plus Dual Xtra protein standards (Bio-Rad).

Ni^{2+} pull-down assay

For Ni^{2+}-pull down assays, His_6-SUMO-CgnA was used as bait, while CgnE or CgnC were used as prey. Briefly, 25 µg of His_6-SUMO-CgnA were loaded onto 5 µL of MagneHis Ni particles (Promega). 25 µg of bait protein was added in 50 µL and incubated with the beads for 20 min. The beads were then washed twice with 25 µL wash buffer (150 mM NaCl, 10 mM HEPES pH 7.4 and 20 mM imidazole). Proteins were eluted off the beads with 50 µL of elution buffer (150 mM NaCl, 10 mM HEPES pH 7.4 and 250 mM imidazole). For controls, either buffer was added as bait, or prey was added to beads that had not been loaded with His_6-SUMO-CgnA. Eluted proteins were analyzed on an SDS-PAGE.

Surface Plasmon Resonance (SPR)

SPR binding affinity measurements were carried out on a Biacore™ X100 system (GE Healthcare, Plus Package Software version 2.0.2). His_6-SUMO-CgnA, CgnB and CgnC were dialyzed in HBS-P buffer (150 mM NaCl, 10 mM HEPES, 0.0005% v/v Surfactant P20, pH 7.4). His_6-SUMO-CgnA was coupled irreversibly onto an Ni-NTA chip following the manufacturer's instructions and as described previously[1]. CgnB was applied with a maximum concentration of 10 µM using a 2-fold dilution series. The K_D was calculated with standard error with the *Biacore X100* software (GE Healthcare, version 2.0.2) and comparable with previously published results (Extended data figure)[1]. CgnC bound non-specifically to the sensor chip and variations of the buffer composition could not resolve this problem.

Large scale protein expression of Cgn constructs

Large scale protein expression of every Cgn construct started by transferring a freshly transformed *E. coli* Lemo21(DE3), *E. coli* Rosetta2(DE3) or *E. coli* BL21(DE3) colony with the respective pSUMO-Cgn construct into 100 mL LB-medium supplemented with 50 µg mL^{-1} Kanamycin and, where appropriate, 34 µg mL^{-1} Chloramphenicol. The cultures were grown for 16 h at 37 °C and 200 rpm and subsequently used to inoculate an expression culture of LB-medium supplemented with the appropriate antibiotics at a ratio of 1:100. This culture was grown at 37 °C and 200 rpm until an OD_{600}= 0.8 was reached, at which point the temperature was decreased for CgnB to 16 °C, while CgnC, CgnD, CgnE and CgnL were grown at 18 °C. CgnB and CgnC cultures were induced with 0.1 mM IPTG, whereas

CgnD, CgnE and CgnL cultures were induced with 0.4 mM IPTG and grown at the aforementioned temperatures for 16 h. Cultures were harvested by centrifugation at 6,200 xg and 4 °C for 10 min after which the respective cell pellet was collected and the supernatant discarded. Cell pellets were frozen at - 80 °C until further use.

Seleno-methionine expression of CgnD and CgnE

For seleno-methionine (SeMet) expression, a culture of LB-medium with the appropriate antibiotics was inoculated with a single, freshly transformed *E. coli* colony carrying the vector with the crystallized construct. For every liter of prepared medium for expression, 50 mL of LB-medium were grown at 37 °C and 200 rpm for 16 h. The cultures were centrifuged at 1,200 xg and 20 °C for 15 min and the cell pellet washed three times in M9-medium (8.5 g L^{-1} Na_2HPO_4, 3 g L^{-1} KH_2PO_4, 1 g L^{-1} NH_4Cl, 0.5 g L^{-1} NaCl) before inoculating M9-medium supplemented with glucose-free nutrient mix and 5% glycerol at a ratio of 1:20. The cultures were grown for 20 min at 37 °C, at which point 40 mg L^{-1} *L*-selenomethionine was added to the culture. The cultures were then grown at 37 °C and 200 rpm until an OD_{600} of 0.6 was reached, whereupon 100 mg L^{-1} each of lysine, phenylalanine and threonine and 50 mg L^{-1} each of isoleucine and valine were added. The cultures were incubated for another 20 min before the protein expression was induced by the addition of 1 mM IPTG. The temperature was reduced to 20 °C and the cells were grown for 24 h. The cells were harvested by centrifugation (6,200 xg, 4 °C, 10 min).

Crystallization and data collection of CgnB, CgnD, CgnE and CgnL

For crystallization, 200 µM CgnB was incubated with 250 µM CgnA for 2 h on ice before setting up initial crystallization trials using different sparse matrix and grid screens (Qiagen). The plates were set up using a Gryphon crystallization robot (Art Robbins Instruments) at 18 °C and were directly moved to 4 °C for further incubation. CgnB crystals were observed after two days of incubation at 4 °C in a condition with a well solution consisting of 170 mM ammonium acetate, 85 mM sodium acetate pH 4.6, 25.5% PEG 4000 and 15% glycerol. Single crystals were cryoprotected without optimization by supplementing the well solution with 32% glycerol, mounted into cryoloops (Hampton Research) and flash frozen in liquid nitrogen until further use. A full dataset of CgnB was collected at the European Synchrotron Radiation Facility (ESRF) at beamline ID-23-1.

Initial crystallization trials of CgnE were set up using a protein concentration of 220 µM at 18 °C. Crystals were observed after 2 days in 200 mM $MgCl_2$, 100 mM Tris pH 8.5 and 20% PEG 8000. Selenomethionine-labelled CgnE could be crystallized in the same condition and optimized by increasing the $MgCl_2$ concentration to 300 mM while staying at a high pH of 8.5 and lowering the PEG 8000 concentration to 15%. Single crystals were cryoprotected by supplementing the crystallization solution with 15% 2R,3R-butanediol, mounted into cryoloops (Hampton Research) and flash frozen in liquid nitrogen. SeMet-CgnE crystals gave better data than native CgnE crystals. A high-redundancy

dataset from SeMet-CgnE crystals at the selenium K-edge was collected at the Swiss Light Source (SLS) Beamline X10SA.

CgnD crystals were small and of very poor quality. To improve the crystals, reductive lysine methylation was employed.[7] Resulting CgnD-Kmeth crystallized after 3 days at 18 °C in a condition with a well solution consisting of 200 mM ammonium sulfate, 100 mM MES pH 6.5 and 30% PEG 5000-MME. Optimization plates for this condition were set up using a Dragonfly liquid handler (TTP Labtech). Single crystals were cryoprotected by supplementing the well solution with 32% glycerol, mounted into cryoloops (Hampton Research) and flash frozen in liquid nitrogen. Data sets were collected at Swiss Light Source Beamline X10SA. SeMet-CgnD-Kmeth crystals were obtained after 2 days at 18 °C in 200 mM ammonium acetate, 100 mM sodium acetate pH 4.6 and 30% PEG4000. This condition was further optimized by the addition of different additives using an Additive Screen (Hampton Research). Optimization plates were set up with further addition of 4% 1-propanole to the aforementioned condition with a Dragonfly Liquid Handler (TTP Labtech). Single crystals were cryoprotected by supplementing the well solution with 32% glycerol, mounted into cryoloops (Hampton Research) and flash frozen in liquid nitrogen. A high redundancy dataset at the selenium K-edge was collected at the Swiss Light Source Beamline X06DA.

For crystallization of CgnL, 300 µM CgnL was incubated with 10 mM SAH for 2 h on ice before setting up initial crystallization trials using different sparse matrix and grid screens (Qiagen). The plates were set up using a Gryphon crystallization robot (Art Robbins Instruments) at 18 °C and crystals were observed after seven days in condition F4 of the Morpheus screen (Molecular dimensions). Single crystals were mounted into cryoloops (Hampton Research) and flash frozen in liquid nitrogen until further use. A full dataset of CgnL was collected at the Deutsches Elektronen Synchrotron (DESY) at beamline P11.

Data processing, structure determination, refinement and structural analysis of Cgn proteins

All data were processed using XDS[8] and POINTLESS[9], AIMLESS[10] and ctruncate implemented in ccp4 (Program suite V7.0.001)[11]. The structures of SeMet-CgnD-Kmeth and SeMet-CgnE were determined using Phenix.AutoSol (Version 1.20-4459)[12] (Se-SAD) followed by several rounds of manual rebuilding in COOT (Version 0.9.6 under Xquartz 11 version 2.8.2)[13] and refinement in Phenix.refine14]. The structures of native CgnB and CgnD-Kmeth were determined by molecular replacement (*phenix.phaser*[15]) using the SeMet-CgnE and SeMet-CgnD-Kmeth structures as search models, respectively. Both structures underwent several rounds of manual rebuilding in COOT[13] and refinement in Phenix.refine[14]. The CgnL structure was determined using the *phaser.MRage*[16] implemented in Phenix followed by *phenix.autobuild*[17].

Final PDB coordinates were analyzed using *MolProbity*[18], used for detection of macromolecular assemblies with the PISA server[19] as well as for structural homology analysis on the DALI server.[20]

All structural images portrayed were rendered in *PyMOL* (The PyMOL Molecular Graphics System Version 1.8.6.0, Schrödinger, LLC).

NMR Spectroscopy

Purified, lyophilized **3a/b** (see above) was dissolved in 20 mM sodium phosphate pH 7.2 containing 5% D_2O. A similar sample of unmodified CgnA was analyzed for comparison. 1D ^1H, 2D {^1H,^1H} NOESY, TOCSY and DQF-COSY and 2D {^{13}C,^1H} HSQC spectra were recorded for each sample on a Bruker AVANCE IIIHD/Ultrashield 600 spectrometer equipped with a TCI cryoprobe using Bruker TopSpin 3.5 software. After excluding peaks from unmodified CgnA in the modified sample, the assignments of the C-terminal residues of the modified CgnA species were made using COSY, TOCSY and inter-residue NOE cross peaks using CCPNMR analysis assign v3. ^{13}C shifts were identified from the HSQC spectra where sensitivity allowed. The majority of the expected resonances were easily identified, but the near-degeneracy of the former tyrosine Hα and Hβ shifts meant that the nOe cross peaks required to identify the stereochemistry at the tyrosine Cβ were not resolved. Indeed, the observation of two separate sets of resonances that likely correspond to modified tyrosine Hδ and Hε protons suggest that either both stereoisomers are present, or that a crocagin B-like modification has been formed.

Labeling of CgnD with PMSF

Phenylmethylsulfonylfluoride (PMSF) can selectively modifying the catalytic serine of serine hydrolases via the formation of a covalent bond with the protein and can therefore be detected even in denaturing LC-MS conditions by a positive mass shift corresponding to the compound[21]. As such, it can be used as an indicator for nucleophilicity of the active site serine residue.

16.4 mg PMSF was dissolved in 1 mL ethanol (99.8%) to yield a 94.15 mM stock solution. Subsequently, the solution was diluted to a 1 mM working concentration in ethanol. For the incubation, CgnD was diluted to a 10 µM concentration in the gel filtration buffer. The final volume was adjusted to 50 µL total, including 5 µL of the 1 mM PMSF solution to reach 10-fold excess of PMSF. As a control, a second sample was prepared without PMSF. Both samples were incubated at room temperature for 10 min prior to LC-MS analysis.

Microscale Thermophoresis

MST binding affinity measurements were performed using a Monolith NT 115 system (Nanotemper Technologies) with MO.Control v1.6 software. Labeling of different proteins was performed using the Monolith Labeling Kit RED-NHS 2nd generation according to the manufacturer's instructions. For this, a 10 µM dilution of the desired protein was prepared in the supplied labeling buffer NHS (130 mM $NaHCO_3$, 50 mM NaCl, pH 8.2). To yield a 600 µM dye solution, 25 µL DMSO was added to dry powder. For the labeling procedure, 7 µL of the freshly prepared dye solution was mixed with 7 µL of

the Labeling Buffer NHS. Subsequently, 10 µL of the diluted dye solution were added to 90 µL of the diluted protein to yield a 3-fold molar excess of dye. The mixture was kept in the dark for 30 minutes at room temperature. Subsequently, the labeled protein was separated from excess dye using the supplied B-column equilibrated in gel filtration buffer + 0.05% Tween-20. The final concentration of the labeled protein was determined via UV-VIS spectrometry. Afterwards, the labeled protein was either used immediately for MST measurements or stored for further use by flash freezing in liquid N_2.

In preparation for a MST measurement, a buffer exchange of the desired ligand into the same buffer was performed. Serial ligand dilutions were created according to the manufacturer's instructions, with the maximum ligand concentration adjusted to the expected K_D. For 3-component measurements, the third component was mixed with the labeled protein prior to being added to the ligand dilutions, ensuring a steady concentration of the third component. The MST experiment was carried out at 25 °C with an automatically detected excitation power, the MST power was set to medium. The subsequent measurements required to achieve biological triplicates were performed with the previously determined excitation power. A K_D measurement with standard error was calculated using the *MO.Affinity Analysis v2.3* software.

LC-MS

All precursor peptide measurements were performed on a Dionex Ultimate 3000 RSLC system using a Waters BEH C18, 50 x 2.1 mm, 1.7 µm dp column. LC software was controlled by Eksigent control software v4.3 and the acquisition software were from Analyst TF1.8.1 from AB Sciex. Separation of 1 µl sample was achieved by a linear gradient with (A) H_2O + 0.1% FA to (B) ACN + 0.1% FA at a flow rate of 600 µL min^{-1} and 45 °C. The gradient was initiated by a 0.5 min isocratic step at 5% B, followed by an increase to 95% B in 9 min to end up with a 1 min step at 95% B before re-equilibration under the initial conditions. UV spectra were recorded by a DAD in the range from 200 to 600 nm. The LC flow was split to 75 µL min^{-1} before entering the maXis 4G hr-ToF mass spectrometer (Bruker Daltonics, Bremen, Germany) using the standard ESI source. Mass spectra were acquired in centroid mode ranging from 150 – 2500 m/z at a 2 Hz scan speed.

Intact protein mass-measurements for CgnD were performed on a Dionex Ultimate 3000 RSLC system using an Aeris Widepore XB-C8, 150 x 2.1 mm, 3.6 µm dp column (Phenomenex, USA). Separation of 2 µl sample was achieved by a linear gradient from (A) H_2O + 0.1% FA to (B) ACN + 0.1% FA at a flow rate of 300 µl min^{-1} and 45 °C. The gradient was initiated by a 1 min isocratic step at 2% B, followed by an increase to 75% B in 10 min to end up with a 3 min step at 75% B before reequilibration with initial conditions. UV spectra were recorded by a DAD in the range from 200 to 600 nm. The LC flow was split to 37.5 µl min^{-1} before entering the maXis 4G hr-ToF mass spectrometer (Bruker Daltonics, Bremen, Germany) using the standard Bruker ESI source. In the source region, the temperature was set to 200 °C, the capillary voltage was 4000 V, the dry-gas flow was 5.0 L min^{-1} and the nebulizer was set to 1.0 bar. Mass spectra were acquired in positive ionization mode ranging from

150 – 2500 m/z at 2.0 Hz scan rate. Protein masses were deconvoluted by using the Maximum Entropy algorithm (Copyright 1991-2004 Spectrum Square Associates, Inc.). MS data were recorded and analyzed using one of the following softwares: Bruker Compass HyStar 5.1.8.1 (Bruker Daltonik GmbH), Bruker Compass 4.1.0.839 (Bruker Daltonik GmbH), Bruker OtofControl Version 5.2 (Build 0.8) (Bruker Daltonik GmbH), Thermo Chromeleon 7.2.10 Build 23925 (Thermo Fisher Scientific Inc.), Peakview Software 1.2.0.3, AB Sciex, OR DataAnalysis 4.4 (Bruker).

MS^2 Analyses

The site of the oxidation of the peptide was ascertained by nLC-MS^2 analysis on a Sciex 5600+ QTOF mass spectrometer. The sample was dissolved in 0.05% trifluoracetic acid and injected using a Eksigent 2D ultra nanoLC onto a ThermoFisherScientfic C18 Acclaim Pepmap trap (100 µm x 2 cm) and washed at 5 µL / min for 5 min in loading buffer (0.05% TFA). The trap was switched in-line with the Acclaim pepmap C18 analytical column (75 µm x 150 mm) and eluted over a short gradient at 300 nL min^{-1}. A (98% water, 2% acetonitrile, 0.1% formic acid), B (98% acetonitrile, 2% water, 0.1% formic acid): starting at 100% A, linear to 60% A, 40% B over 6 min, linear to 100% B over 2 min, hold at 100% B for 2 min, returning to 100% A and re-equilibration for 10 min. MS data over 400-1250 m/z, and MS^2 in product ion scan mode with a collision voltage of 45 V were collected on 613.8 m/z and 617.8 m/z, acquired over the length of the LC run (20 min). MS^2 spectra were combined over the elution of the peaks of interest at 10.5 min and the spectra annotated manually in Peakview software.

LC-MS analysis of reactions with CgnL, truncated CgnA, $CgnA^{N3A}$ and $CgnA^{C3A}$

Samples were analysed on a Dionex Ultimate 3000 RSLS nano flow system (Dionex, Camberly UK) using Xcallbur, version 2.2 service pack 1.48 (Thermo Scientific). The samples (5 µL) were loaded onto a Dionex 100 µm x 2 cm 5 µm C18 nano trap column at a flowrate of 5 µL min^{-1} by a Ultimate 3000 RS autosampler (Dionex, Camberley UK) The composition of the loading solution was 0.1% formic acid and acetonitrile (98 : 2). Once loaded onto the trap column the sample was then washed off into an Acclaim PepMap C18 nano column 75 µm x 50 cm, 2µm 100 Å at a flowrate of 0.3 µm min^{-1}. The trap and nano flow column were maintained at 35 ºC in a column oven in the Ultimate 3000 RSLC. The samples were eluted with a gradient of solvent A: 0.1% formic acid verses solvent B: 80% acetonitrile, 20% water in 0.1% formic acid starting at 5% B rising to 60% B over 15 mins. The column was washed using 90% B before being equilibrated prior to the next sample being loaded.

The eluent from the column was directed to a Proxeon nano spray ESI source (Thermo Fisher Hemel UK) operating in positive ion mode then into an Orbitrap Velos FTMS. The ionisation voltage was 2.8 kV and the capillary temperature was 250 ºC. The mass spectrometer was operated in MS-MS mode scanning from 380 to 750 amu with multiply charged ions selected for MS/MS analysis using HCD at 35 % collision energy. Resolution in MS1 was 60,000. MS data was analysed with Xcalibur, version 2.2 service pack 1.48 from Thermo Scientific.

Inductively Couple Plasma-Mass Spectrometry

Experimental solutions were analysed using an Agilent 7500ce fitted with a self-aspire nebuliser tuned for a flow of 0.1 mL min^{-1} at the Scottish Universities Environmental Research Centre (SUERC). Samples were initially diluted to a protein concentration of 2 µM in 0.8 M HNO_3. CgnB was dialyzed 4 x for 1 h at a dilution of 1 : 5000 in buffer containing 100 mM EDTA prior to analysis. CgnE was incubated with freshly prepared $FeCl_2$ for 30 min (5 mM final $FeCl_2$ concentration) and then subjected to a PD10 desalting column in CgnE buffer before the protein was diluted in HNO_3 for analysis. For Fe^{2+} analysis, flow in the collision cell was set to 2 mL min^{-1} of H. The metal concentration was calculated by reference to a three-standard calibration line. Each sample value was corrected for procedural blank containing ultrapure water and 0.8 M HNO_3.

Bioinformatics

CgnB and CgnE sequences were compared with TBLASTN[22] to whole-genome shotgun contigs of taxa listed in (Supplementary table 4). We have retrieved the corresponding genome segments and identified tandem appearances of homologous genes, as well as searched for candidate peptides in their genomic neighborhood with custom Python and Perl scripts. Phylogenetic trees were constructed with RAxML[23] using the PROTGAMMAJTT model. Bootstrap values were computed using 100 bootstrap runs[24]. We did not consider an outgroup due to the lack of significant sequence similarity to aminopeptidases (Suppl. Figure 9), hence all trees should be considered as unrooted. For visualization we used the ape package in R[25] and placed the root at the longest internal branch.

To compare the genomic neighborhood of the identified duplicated CgnB/E homologs, we selected a genomic region of 10,000 bp upstream and downstream of the CgnA precursor peptide and predicted ORFs in it with Geneious. After that for each translated ORF, we identified all significantly similar Pfam families with HMMer[26] (significance threshold 1e-05). To calculate sequence similarity between a pair of genomic regions, we aligned all pairs of translated ORFs that share at least one Pfam annotation with MUSCLE[27] and added up fraction of identical residues for all such pairs. The heatmap was constructed with R.

Supplementary Table 1: Errors associated with mass spectra reported in this study organized by figure. Supplementary figures are abbreviated with "S". Mass shifts are simplified. Actual mass shifts are: + 12 Da = + 11.9636 Da, + 14 Da = + 13.9793 Da, +16 Da = + 15.9949 Da, +28 Da = + 27.9586 Da and +30 Da = + 29.9742 Da.

Figure		Mass shift (Da)	Calculated (Da)	Observed (Da)	Δppm
4a	**4a**		$[M]^{+}$ = 495.2238	$[M]^{+}$ = 495.2239	0.2
4c	**5a**		$[M]^{+}$ = 509.2395	$[M]^{+}$ = 509.2391	-0.8
S3a	CgnA		$[M]^{5+}$ = 491.2757	$[M]^{5+}$ = 491.2762	1.0
S3b	CgnA + CgnB		$[M]^{5+}$ = 491.2757	$[M]^{5+}$ = 491.2758	0.2
S3c	CgnA + CgnE		$[M]^{5+}$ = 491.2757	$[M]^{5+}$ = 491.2762	1.0
S3d	CgnA + CgnB/E		$[M]^{5+}$ = 491.2757	$[M]^{5+}$ = 491.2751	-1.2
S5a	CgnA + CgnC no cof		$[M]^{5+}$ = 491.2757	$[M]^{5+}$ = 491.2762	1.0
S5b	CgnA + CgnC		$[M]^{5+}$ = 491.2757	$[M]^{5+}$ = 491.2766	1.8
		+ 16 Da	$[M]^{5+}$ = 494.4747	$[M]^{5+}$ = 494.4755	1.6
S5c	CgnA + CgnB/C		$[M]^{5+}$ = 491.2757	$[M]^{5+}$ = 491.2766	1.8
		+ 16 Da	$[M]^{5+}$ = 494.4747	$[M]^{5+}$ = 494.4752	1.0
		+ 14 Da	$[M]^{5+}$ = 494.0716	$[M]^{5+}$ = 494.0725	1.8
		+ 28 Da	$[M]^{5+}$ = 496.8533	$[M]^{5+}$ = 496.8551	3.6
		+ 30 Da	$[M]^{5+}$ = 497.2705	$[M]^{5+}$ = 497.2714	1.8
S5d	CgnA + CgnC/E		$[M]^{5+}$ = 491.2757	$[M]^{5+}$ = 491.2769	2.4
		+ 16 Da	$[M]^{5+}$ = 494.4747	$[M]^{5+}$ = 494.4749	0.4
		+ 28 Da	$[M]^{5+}$ = 496.8533	$[M]^{5+}$ = 496.8533	0.0
S5e	CgnA + CgnB/C/E	+ 14 Da	$[M]^{5+}$ = 494.0716	$[M]^{5+}$ = 494.0714	-0.4
		+ 30 Da	$[M]^{5+}$ = 497.2705	$[M]^{5+}$ = 497.2698	-1.4
S5f	CgnA		$[M]^{5+}$ = 491.2757	$[M]^{5+}$ = 491.2762	1.0
S5f	CgnA + CgnB/C/E	+ 14 Da	$[M]^{5+}$ = 494.0716	$[M]^{5+}$ = 494.0714	-0.4
S5g	CgnA + CgnB/C/E	+ 12 Da	$[M]^{5+}$ = 493.6684	$[M]^{5+}$ = 493.6674	-2.0
S16a	CgnA_K2E		$[M]^{5+}$ = 491.4652	$[M]^{5+}$ = 491.4658	1.2
S16b	CgnA_K2E + CgnB/C/E		$[M]^{5+}$ = 491.4652	$[M]^{5+}$ = 491.4656	0.8
		+ 14 Da	$[M]^{5+}$ = 494.2611	$[M]^{5+}$ = 494.2615	0.8
		+ 16 Da	$[M]^{5+}$ = 494.6642	$[M]^{5+}$ = 494.6646	0.8
		+ 30 Da	$[M]^{5+}$ = 497.4600	$[M]^{5+}$ = 497.4604	0.8
S16c	CgnA_G17F		$[M]^{5+}$ = 509.2851	$[M]^{5+}$ = 509.2860	1.8
S16d	CgnA_G17F + CgnB/C/E		$[M]^{5+}$ = 509.2851	$[M]^{5+}$ = 509.2862	2.2
		+ 14 Da	$[M]^{5+}$ = 512.0801	$[M]^{5+}$ = 512.0820	3.7
		+ 16 Da	$[M]^{5+}$ = 512.4841	$[M]^{5+}$ = 512.4848	1.4
		+ 30 Da	$[M]^{5+}$ = 515.2799	$[M]^{5+}$ = 515.2809	1.9
S16e	CgnA_I19G		$[M]^{5+}$ = 480.0632	$[M]^{+}$ = 480.0639	1.5

S16f	CgnA_I19G	+ 14 Da	$[M]^{5+}$ = 482.8590	$[M]^{+}$ = 482.8595	1.0
		+ 16 Da	$[M]^{5+}$ = 483.2622	$[M]^{+}$ = 483.2624	0.4
		+ 30 Da	$[M]^{5+}$ = 486.0580	$[M]^{+}$ = 486.0584	0.8
S17a	CgnA_N3A		$[M]^{3+}$ = 761.0634	$[M]^{3+}$ = 761.0673	5.1
S17b	CgnA_N3A + CgnB/C/E	+ 14 Da	$[M]^{3+}$ = 765.7231	$[M]^{3+}$ = 765.7250	2.5
S17c	CgnA_C3A		$[M]^{3+}$ = 751.7281	$[M]^{3+}$ = 751.7300	2.5
S17d	CgnA_C3A+ CgnB/C/E	+ 14 Da	$[M]^{3+}$ = 756.3878	$[M]^{3+}$ = 756.3883	0.7
S17e	$CgnA^{-5}$		$[M]^{3+}$ = 617.6673	$[M]^{3+}$ = 617.6663	-1.6
S17f	$CgnA^{-5}$ + CgnB/C/E	+ 14 Da	$[M]^{3+}$ = 622.3270	$[M]^{3+}$ = 622.3256	-2.2
S17g	$CgnA^{-10}$		$[M]^{3+}$ = 441.8983	$[M]^{3+}$ = 441.8978	-1.1
S17h	$CgnA^{-10}$ + CgnB/C/E		$[M]^{3+}$ = 441.8983	$[M]^{3+}$ = 441.8976	-1.6
S18a	**3a**		$[M]^{5+}$ = 494.0716	$[M]^{5+}$ = 494.0720	0.8
S18b	**4a**		$[M]^{+}$ = 495.2238	$[M]^{+}$ = 495.2239	0.2
S18c	**4b**		$[M]^{+}$ = 493.2082	$[M]^{+}$ = 493.2083	0.2
S18d	CgnA_N18K		$[M]^{5+}$ = 494.0861	$[M]^{5+}$ = 494.0843	-3.6
S18e	CgnA_N18K + CgnB/C/E	+ 14 Da	$[M]^{5+}$ = 496.8820	$[M]^{5+}$ = 496.8845	5.0
S18f	**4a**		$[M]^{+}$ = 495.2238	$[M]^{+}$ = 495.2228	-2.0
S21	**5b**		$[M]^{+}$ = 507.2238	$[M]^{+}$ = 507.2239	0.2
S23c	CgnA_Y20F		$[M]^{5+}$ = 488.0767	$[M]^{5+}$ = 488.0772	1.0
S23d	CgnA_Y20F + CgnB/C/E		$[M]^{5+}$ = 488.0767	$[M]^{5+}$ = 488.0778	2.3
		+ 14 Da	$[M]^{5+}$ = 490.8726	$[M]^{5+}$ = 490.8736	2.0
		+ 16 Da	$[M]^{5+}$ = 491.2757	$[M]^{5+}$ = 491.2768	2.2
S23e	Core + CgnB/C/E		$[M]^{+}$ = 481.2445	$[M]^{+}$ = 481.2444	-0.2
S23f	Core + CgnB/C/E + leader		$[M]^{+}$ = 481.2445	$[M]^{+}$ = 481.2458	2.7
		+ 14 Da (3)	$[M]^{+}$ = 495.2238	$[M]^{+}$ = 495.2253	3.0
		+ 16 Da (1)	$[M]^{+}$ = 497.2395	$[M]^{+}$ = 497.2398	0.6
		+ 16 Da (2)	$[M]^{+}$ = 497.2395	$[M]^{+}$ = 497.2406	2.2
		+ 16 Da (3)	$[M]^{+}$ = 497.2395	$[M]^{+}$ = 497.2404	1.8

Supplementary Table 2: NMR table

NmrResidue	NmrChain	Sequence	Type	Name	Mod #	Mod	CgnA	CgnA #
M.20.TYR	M	20	TYR	CA	13	56,61	56,76	14
M.20.TYR	M	20	TYR	CB	14	62,58	61,96	15
M.20.TYR	A	20	TYR	CD%	16/20	n.d.	133,51	17/21
M.20.TYR	A	20	TYR	CE%	17/19	n.d.	117,31	18/20
M.20.TYR	M	20	TYR	HA	13	5,31	5,32	14
M.20.TYR	M	20	TYR	HBx	14	5,31	5,27	15
M.20.TYR	A	20	TYR	HD%	16/20	two sets	7,11	17/21
M.20.TYR	A	20	TYR	HE%	17/19	two sets	6,57	18/20
M.21.TRP	M	21	TRP	CA	2	72,44	66,65	2
M.21.TRP	M	21	TRP	CB	3	83,86	84,36	3
M.21.TRP	M	21	TRP	CD1	5	79,48	78,46	5
M.21.TRP	M	21	TRP	CE3	7	127,39	127,11	7
M.21.TRP	M	21	TRP	CG	4	56,12	54,36	4
M.21.TRP	M	21	TRP	CH2	9	n.d.	129,81	9
M.21.TRP	M	21	TRP	CZ2	10	115,02	113,21	10
M.21.TRP	M	21	TRP	CZ3	8	121,60	120,01	8
M.21.TRP	M	21	TRP	HA	2	4,26	4,25	2
M.21.TRP	M	21	TRP	HBx	3	4,79	5,38	3
M.21.TRP	M	21	TRP	HD1	5	5,88	5,98	5
M.21.TRP	M	21	TRP	HE3	7	7,24	7,24	7
M.21.TRP	M	21	TRP	HGx	4	3,91	3,96	4
M.21.TRP	M	21	TRP	HH2	9	6,75	6,68	9
M.21.TRP	M	21	TRP	HZ2	10	5,46	5,39	10
M.21.TRP	M	21	TRP	HZ3	8	6,69	6,54	8

Supplementary Table 3: Data collection and refinement statistics

	**CgnB**	**SeMet-CgnE**	**CgnD-Kmeth**	**SeMet-CgnD-Kmeth**	**CgnL**
**PDB code**	6zsv	6zsu	8a2n	8a2n	7pd7
**Data collection**					
Space group	$P2_1$	$P2_12_12_1$	$P2_12_12_1$	$P2_1$	$P2_1$
Cell dimensions					
a, *b*, *c* (Å)	52.4, 67.5, 86.2	70.4, 75.3, 112.4	46.5, 102.1, 150.8	102.3, 61.1, 118.9	41.0, 128.1, 94.3
α, β, γ (°)	90.0, 96.0, 90.0	90.0, 90.0, 90.0	90.0, 90.0, 90.0	90.0 90.2 90.0	90.0, 90.39, 90.0
Wavelength (Å)	0.9724	0.9780	1.1801	0.9783	1.0332
Resolution (Å)	36.17 – 2.30 (2.38 – 2.30)	45.06 – 2.00 (2.05 – 2.00)	39.56 – 2.35 (2.41 – 2.35)	48.03 – 2.60 (2.74 – 2.60)	47.12 – 1.96 (2.00 – 1.96)
$CC_{1/2}$	0.060 (0.454)	0.117 (0.409)	0.998 (0.955)	0.119 (0.467)	0.997 (0.519)
I / σI	9.8 (2.1)	16.9 (7.0)	21.2 (8.0)	31.7 (8.8)	8.1 (1.0)
Completeness (%)	96.5 (97.4)	99.9 (99.8)	99.8 (98.2)	100.0 (99.9)	100.0 (100.0)
Redundancy	2.9 (2.9)	12.7 (13.0)	12.6 (11.5)	28.0 (28.8)	9.2 (9.3)
**Refinement**					
Resolution (Å)	36.17 – 2.30	45.06 – 2.00	39.56 – 2.35		47.12 – 1.96
No. reflections	25558 (2558)	41108 (4055)	30658 (2992)		69552 (6931)
R_{work} / R_{free}	0.283 / 0.305	0.153 / 0.200	0.172 / 0.224		0.185/0.223
No. atoms	4899	5111	5097		8167
Protein	4834	4597	4836		7723
Ligand/ion	4	-	10		42
Water	61	514	251		402
B-factors	61.24	24.64	36.07		38.25
Protein	61.37	23.95	35.97		37.99
Ligand/ion	32.78	-	43.79		47.69
Water	52.48	30.83	37.62		42.19
R.m.s. deviations					
Bond lengths (Å)	0.005	0.014	0.015		0.012
Bond angles (°)	0.98	1.46	1.53		1.27
MolProbity clash score	10.4	4.5	8.6		5.9
Ramachandran (%)					
Favoured	96.56	97.70	96.07		97.57
Allowed	3.28	2.30	3.56		2.33
Outliers	0.16	0.00	0.37		0.10

Statistics for the highest resolution shell are shown in parentheses. Data for SeMet-CgnD-Kmeth has been deposited to the PDB as a second dataset for 8a2n.

Supplementary Table 4: Genomic loci with identified two copies of CgnB/E homologs and precursor peptides. The core peptide is marked in bold red.

Genome identifier	Coordinates, copy 1		Coordinates, copy 2		Candidate precursor peptide	Position	Direction	Organism
APIY01000009	24244	25176	21722	22621	MNAKKTQKRKLEQVNKETRSNLGN**VYW**	22793	complement	Dolichospermum circinale AWQC131C
BJCF01000037	39455	40387	36939	37838	MNAKKTQKRKLEQVNKETRSNLGN**VYW**	38010	complement	Dolichospermum planctonicum NIES-80
CACVAY010000112	59452	60354	60379	61302	MKKAKQVSKANRKARQN**AYW**	61434	complement	uncultured Thiotrichaceae bacterium isolate
CP011456	3953702	3954634	3951067	3951966	MNAKKTQKRKLEQVNKETRSNLGN**IYW**	3952139	complement	Anabaena sp. WA102
CP012159	3375719	3376693	3378736	3379662	MTMKKTIKQISAGDRSKKGN**IYW**	3379825	complement	Chondromyces crocatus strain Cm c5
FTPM01000001	2442254	2443153	2443650	2444552	MNQAQQVKVRPVSPEERRIAQN**YYW**	2444643	complement	Burkholderia sp. b13
JAAHHU010000011	20291	21199	19326	20255	MKKEIKKETQHKETASKKRKLEQVDKNTRSKLGN**VYW**	21610	complement	Moorea sp. SIO3I6
JAAHHV010000001	12556	13467	11591	12520	MKKEIKKETQHKETASKKRKLEQVDKNTRSKLGN**VYW**	13878	complement	Moorea sp. SIO3I7
JAAHHW010000011	29612	30523	28647	29576	MKKEIKKETQHKETASKKRKLEQVDKNTRSKLGN**VYW**	30934	complement	Moorea sp. SIO3I8
JAAHHX010000048	29436	30344	28471	29400	MKKEIKKETQHKETASKKRKLEQVDKNTRSKLGN**VYW**	30755	complement	Moorea sp. SIO4A1
JAAHHZ010000002	104120	105028	103155	104084	MKKEIKKETQHKETASKKRKLEQVDKNTRSKLGN**VYW**	105439	complement	Moorea sp. SIO4A5
JAAHIG010000012	25820	26728	24855	25784	MKKEIKKETQHKETASKKRKLEQVDKNTRSKLGN**GYW**	27127	complement	Moorea sp. SIO4G3
KE384588	83314	84246	80792	81691	MNAKKTQKRKLEQVNKETRSNLGN**VYW**	82290	complement	Dolichospermum circinale AWQC131C
LJOQ01000040	77606	78541	80279	81178	MNAKKTQKRKLEQVNKETRSNLGN**IYW**	80103	forward	Anabaena sp. AL09
LJOS01000033	34781	35713	37629	38528	MSAKKTQKRKLEQVNKETRSNLGN**VYW**	37444	forward	Anabaena sp. WA113
LJOU01000015	42230	43162	39593	40492	MNAKKTQKRKLEQVNKETRSNLGN**IYW**	40667	complement	Anabaena sp. AL93
LJOW01000058	12755	13687	15610	16509	MSAKKTQKRKLEQVNKETRSNLGN**VYW**	15425	forward	Aphanizomenon flos-aquae WA102
LJOX01000057	5835	6767	8855	9754	MNAKKTQKRKLEQVNKETRSNLGN**IYW**	8679	forward	Aphanizomenon flos-aquae MDT14a
LJOY01000036	22890	23822	20102	21001	MNAKKTQKRKLEQVNKETRSNLGN**IYW**	21176	complement	Aphanizomenon flos-aquae LD13
VILE01000010	46019	46951	48691	49590	MNAKKTQKRKLEQVNKETRSNLGN**VYW**	48413	forward	Dolichospermum planctonicum UHCC 0167
VILF01000001	1460095	1461027	1462805	1463704	MNAKKTQKRKLEQVNKETRSNLGN**AYW**	1462620	forward	Dolichospermum flos-aquae UHCC 0037

Supplementary Table 5: Gene annotations of the crocagin gene cluster and the homologous gene clusters shown in Figure 3d. The closest homologue was identified with HHpred.

Gene	Closest homologue	Sequence identity (%)	UniprotKB
CgnB	Aminopeptidase	15	A0A0H2UN95
CgnC	Monooxygenase	17	A0A1J4PXK4
CgnD	Hydrolase	14	D1BPP2
CgnE	Aminopeptidase	11	A0A0H3K3S3
CgnF	ABC transporter	19	Q9WYC3
CgnG	ABC transporter	17	Q9WYC3
CgnJ	Methyltransferase	15	Q97GJ5
TetR	Transcriptional regulator	17	Q7CR15
CgnI	Carbamoyltransferase	35	Q70IY1
CgnK	Nuclease inhibitor	12	P12669
CgnL	Oxidoreductase	29	Q5FQJ0
ThioB	Aminopeptidase	15	A0A0H2UN95
ThioC	Aminopeptidase	13	A0A0H2UN95
ThioD	Methyltransferase	17	Q97GJ5
ThioE	Glycosyltransferase	23	Q8KNE0
ThioF	Monooxygenase	18	A0A1J4PXK4
ThioG	Sulfotransferase	17	B7ZWN4
ThioH	Methyltransferase	13	Q9BV86
ThioI	MFS transporter	13	A0LNN5
ThioJ	Hydrolase	36	Q15661
ThioK	Hypothetical protein	27	Q142Q8
MooB	Aminopeptidase	15	A0A0H3K3S3
MooC	Aminopeptidase	15	A0A0H3K3S3
MooD	Glycosyltransferase	23	Q8KNE0
MooE	Dioxygenase	34	A0A0E3URV8
MooF	Methyltransferase	18	Q5VVY1
MooG	Lyase	17	P84193
MooH	MFS transporter	15	P31122
AnaB	Aminopeptidase	17	A0A0H3K3S3
AnaC	Glycosyltransferase	25	Q8KNE0
AnaD	Dioxygenase	35	A0A0E3URV8
AnaE	Methyltransferase	15	Q6D6E7
AnaF	MATE transporter	17	Q8U2X0
AnaG	Acetyltransferase	40	Q7ATH7
AnaH	Oxidoreductase	25	P32340
AnaI	Dehalogenase	19	Q9I5C9
AnaJ	Methyltransferase	16	Q5VVY1
AnaK	Aminopeptidase	11	A0A0H3K3S3
AnaL	Orotidine-5-phosphate decarboxylase	37	E9BCQ9
AnaM	Phosphatidylserine synthase	12	P44704
AnaN	Cysteine desulfurase	39	O25008
DolB	Aminopeptidase	17	A0A0H3K3S3
DolC	Glycosyltransferase	25	Q8KNE0
DolD	Dioxygenase	35	A0A0E3URV8
DolE	Methyltransferase	17	Q6D6E7
DolF	MATE transporter	17	Q8U2X0
DolG	Orotidine-5-phosphate decarboxylase	37	E9BCQ9
DolH	Methyltransferase	17	Q5VVY1
DolI	Aminopeptidase	11	A0A0H3K3S3
DolJ	MATE transporter	16	Q8U2X0

Supplementary Table 6: Hydrogen bonds and salt bridges found at the CgnD dimer interface that were identified by PISA.

HYDROGEN BONDS	CHAIN B	DIST. [Å]	CHAIN A
1	B:TYR 29[HH]	2.17	A:SER 159[O]
2	B:ARG 35[HH11]	2.22	A:PRO 202[O]
3	B:PHE 124[O]	2.44	A:ARG 224[HH11]
4	B:SER 159[O]	2.20	A:TYR 29[HH]
5	B:PRO 202[O]	1.82	A:ARG 35[HH11]
6	B:GLU 223[OE1]	1.93	A:ARG 127[HH22]

SALT BRIDGES	CHAIN B	DIST. [Å]	CHAIN A
1	B:ARG 48[NE]	3.28	A:ASP 156[OD2]
2	B:ARG 35[NH1]	3.29	A:GLU 203[OE2]
3	B:ARG 127[NH2]	3.83	A:GLU 223[OE1]
4	B:ASP 156[OD2]	3.36	A:ARG 48[NE]
5	B:GLU 203[OE2]	3.50	A:ARG 35[NH1]
6	B:GLU 223[OE1]	2.56	A:ARG 127[NH2]

Supplementary Table 7: Primary amino acid sequences of synthetic peptides used in this manuscript.

**Construct**	**Primary amino acid sequence**
CgnA	MKKTIKQISAGDRSKKGNIYW
CgnA_leader	MKKTIKQISAGDRSKKGN
CgnA_core	IYW
CgnA-5	KQISAGDRSKKGNIYW
CgnA-10	GDRSKKGNIYW
CgnA_K2E	MEKTIKQISAGDRSKKGNIYW
CgnA_G17F	MKKTIKQISAGDRSKKFNIYW
CgnA_N18K	MKKTIKQISAGDRSKKGKIYW
CgnA_I19G	MKKTIKQISAGDRSKKGNGYW
CgnA_F20Y	MKKTIKQISAGDRSKKGNIFW
CgnA_N3A	MAATIAQISAGDRSKKGNIYW
CgnA_C3A	MKKTIKQISAGDASAAGNIYW

Supplementary Table 8: Primary amino acid sequences of recombinant Cgn enzymes used in this manuscript.

Construct	Primary amino acid sequence
CgnB	GAMADIGSMDVLEYFERLKNRELAFVLDDLQLSDMVTRRGFSVIPFDDFD<br>LAREDHPPAFVLVTRLDYHGKLMQAWETAKGISSHLSLAKFDTSPKSVEY<br>SLDQLLSMDFAETLKRRGDYYDSVASTNRMEVVTPGAVLTCDFGNEIEIA<br>NNDVEMQKGWLYSVAEFFETSVINLEADRSSYTLNGDLCFTGLIYLCNRP<br>DLKERASATMDELMRMSTRGRNVVSFVDNQIVRMELGGVDMTATLRELI<br>VGKEREGSSTEFAMGCVEYPLAQDWTINSVMNEGSHGIHVGVGMGKEIP<br>HMDFIAKGAELRIAESSDA
CgnC	GAMVHLTATDLHVDFSGDLEAALAKARHLYATFGGFVAARLFDAADLTP<br>IHEELGRLIALAAREVDGFSLPSPRTRFDEGFHALHEEAPAAADAVIQAAR<br>RLTTVHELSVNPRLLSVSRRLMGTELVMSNPYKPIRVDYAEREHFLLPWH<br>QDYPYAQDSMDAVVHWIPLQDVDEQNGCLKVAPGSHELGVVPVKMILPP<br>EGSTHGIRGLQIADPSVVERFPQVSLPMKFGDVLVFSTLLLHRSQLNLTGE<br>ARWTAQVRHGNFEHPLSVQKRWPRGHYERHWFDETHPEYVRPAGS
CgnD	GAMASTTLETRDELTPQMKEYDRAGRVWVPYLNYFHRPNHRSPVVNTDS<br>RGFRFVVGKDGRTFSEFEREPGERVRALVGGSTVFGVGATGDAATLPSLL<br>SQRGPARWLNFGGRAFSSTQELMLFLFHARSLGALEKVTLLSGVNNLLLF<br>YLSRDYAKDYGSFFSATEVRRAFAGDAPSPAKSGVIGRLKSIAGGRRAKV<br>EAPEPEIVLPIVDHDAQKTDLLHAIERDLSTWKLLSGALQFELCYVLQPLA<br>GWVRKKPSPEETRLFADLDDQQGEAWRQILREKMDLAQYAWFSKSLADI<br>CRTQEIPFLDMNATLSALDLDGRWIFVDRVHLTDEGNEVLTQALVEGGAT
CgnE	GAMGGRRTIGIRSGEGAIMNASDFYALLRGRGMPVVVDDAEAAAVVSEL<br>GFRTVPFEAFDFDSPSEDPALVIVAQMGNVDALHGLWERSGTPLMHLALA<br>KFDGGLSRLRAGLARVLAVDTDAALKRRAEAYEQLFSSASVEIASGEGVL<br>RCHIGDEVEVGNCGDTLEQGFLYSVAEFLEASVVNLEGERSTFWVEGELP<br>FDGFIHLSNSAALKERWGGMLDEFMRRSREGANLVRFADNVIDRLVVGG<br>VDVTSALAGLSQGEERGMAATEFGLGCADAEAAEPFGVNSLLHKSAGGA<br>YIGIGKGLRIPHIDFIARGATIRFIPAAEG
CgnL	GAMGGGGYHLFNELAHSYDLHTPPENFQHDHAFVLEEARSLGTPCRLLD<br>VGCGTGALLEKARNAGILATGIDASPKMVELAQARVGQEAVTLRRMEEI<br>DEEGAYDLVVSLCWTIHYSAGRAGLLDVLKRIHRALRPGGRGIIQIAHAA<br>HAPKRTLESRIPGPNGEPDDVVMLFRFRPAPTEEPSMHAEYVYACKSLNE<br>LLYENHLLSMTDAHAFAACAREAGFAQVTVYDSSKRAPFSAAPNPLVCV<br>EKAHDVGR

References

[1] Viehrig K, Surup F, Volz C, Herrmann J, Abou Fayad A, Adam S, Köhnke J, Trauner D, Müller R *Angew Chem Int Ed Engl.* **2017** *56*(26):7407-7410.

[2] Sievers F, Wilm A, Dineen D, Gibson TJ, Karplus K, Li W, Lopez R, McWilliam H, Remmert M, Söding J, Thompson JD, Higgins DG. *Mol Syst Biol.* **2011** *7*:539.

[3] Stivala A, Wybrow M, Wirth A, Whisstock JC, Stuckey PJ. *Bioinformatics.* **2011** *27*(23):3315-6.

[4] Santos-Aberturas J, Chandra G, Frattaruolo L, Lacret R, Pham TH, Vior NM, Eyles TH, Truman AW. *Nucleic Acids Res.* **2019** *47*(9):4624-4637.

[5] Laskowski RA, Swindells MB. *J Chem Inf Model.* **2011** *51*(10):2778-86.

[6] Liu, H; Naismith, JH *Protein Expr Purif* **2009** *6*(2):102-11.

[7] Walter TS, Meier C, Assenberg R, Au KF, Ren J, Verma A, Nettleship JE, Owens RJ, Stuart DI, Grimes JM *Structure.* **2006** *14*(11):1617-22.

[8] Kabsch W *Acta Cryst.* **2010** *D66*:125-132.

[9] Evans PR *Acta Cryst.* **2006** *D62*:72-82.

[10] Evans PR, Murshudov GN *Acta Crystallogr D Biol Crystallogr.* **2013** *69*(Pt 7):1204-14.

[11] Collaborative Computational Project, Number 4. *Acta Crystallogr D Biol Crystallogr.* **1994** *50*(Pt 5):760-3.

[12] Terwilliger TC, Adams PD, Read RJ, McCoy AJ, Moriarty NW, Grosse-Kunstleve RW, Afonine PV, Zwart PH, Hung LW *Acta Crystallogr D Biol Crystallogr.* **2009** *65*(Pt 6):582-601.

[13] Emsley P, Lohkamp B, Scott WG, Cowtan K *Acta Crystallogr D Biol Crystallogr.* **2010** *66*(Pt 4):486-501.

[14] Afonine PV, Grosse-Kunstleve RW, Echols N, Headd JJ, Moriarty NW, Mustyakimov M, Terwilliger TC, Urzhumtsev A, Zwart PH, Adams PD *Acta Crystallogr D Biol Crystallogr.* **2012** *68*(Pt 4):352-67.

[15] McCoy AJ, Grosse-Kunstleve RW, Adams PD, Winn MD, Storoni LC, Read RJ *J Appl Crystallogr.* **2007** 40(Pt 4):658-674.

[16] Bunkóczi G, Echols N, McCoy AJ, Oeffner RD, Adams PD, Read RJ. *Acta Crystallogr D Biol Crystallogr.* **2013** *69*(Pt 11):2276-86.

[17] Terwilliger TC, Grosse-Kunstleve RW, Afonine PV, Moriarty NW, Zwart PH, Hung LW, Read RJ, Adams PD. *Acta Crystallogr D Biol Crystallogr.* **2008** *64*(Pt 1):61-9.

[18] Chen VB, Arendall WB 3rd, Headd JJ, Keedy DA, Immormino RM, Kapral GJ, Murray LW, Richardson JS, Richardson DC *Acta Crystallogr D Biol Crystallogr.* **2010** *66*(Pt 1):12-21.

[19] Krissinel E, Henrick K *J. Mol. Biol.* **2007** *372*, 774-797.

[20] Holm L, Laakso LM *Nucleic Acids Res.* **2016** *44*(W1):W351-5.

[21] Ryan A, Keany S, Eleftheriadou O, Ballet R, Cheng HY, Sim E. *FEMS Microbiol Lett.* **2014** *350*(1):42-7.

[22] Camacho C, Coulouris G, Avagyan V, Ma N, Papadopoulos J, Bealer K, Madden TL. *BMC Bioinformatics.* **2009** *10*:421.

[23] Stamatakis A. *Bioinformatics.* **2014** *30*(9):1312-3.

[24] Stamatakis A, Hoover P, Rougemont *J. Syst Biol.* **2008** *57*(5):758-71.

[25] Paradis E, Schliep K. *Bioinformatics.* **2019** *35*(3):526-528.

[26] Eddy SR. *PLoS Comput Biol.* **2011** *7*(10):e1002195.

[27] Edgar RC. *Nucleic Acids Res.* **2004** *32*(5):1792-7.

Full, unprocessed gels

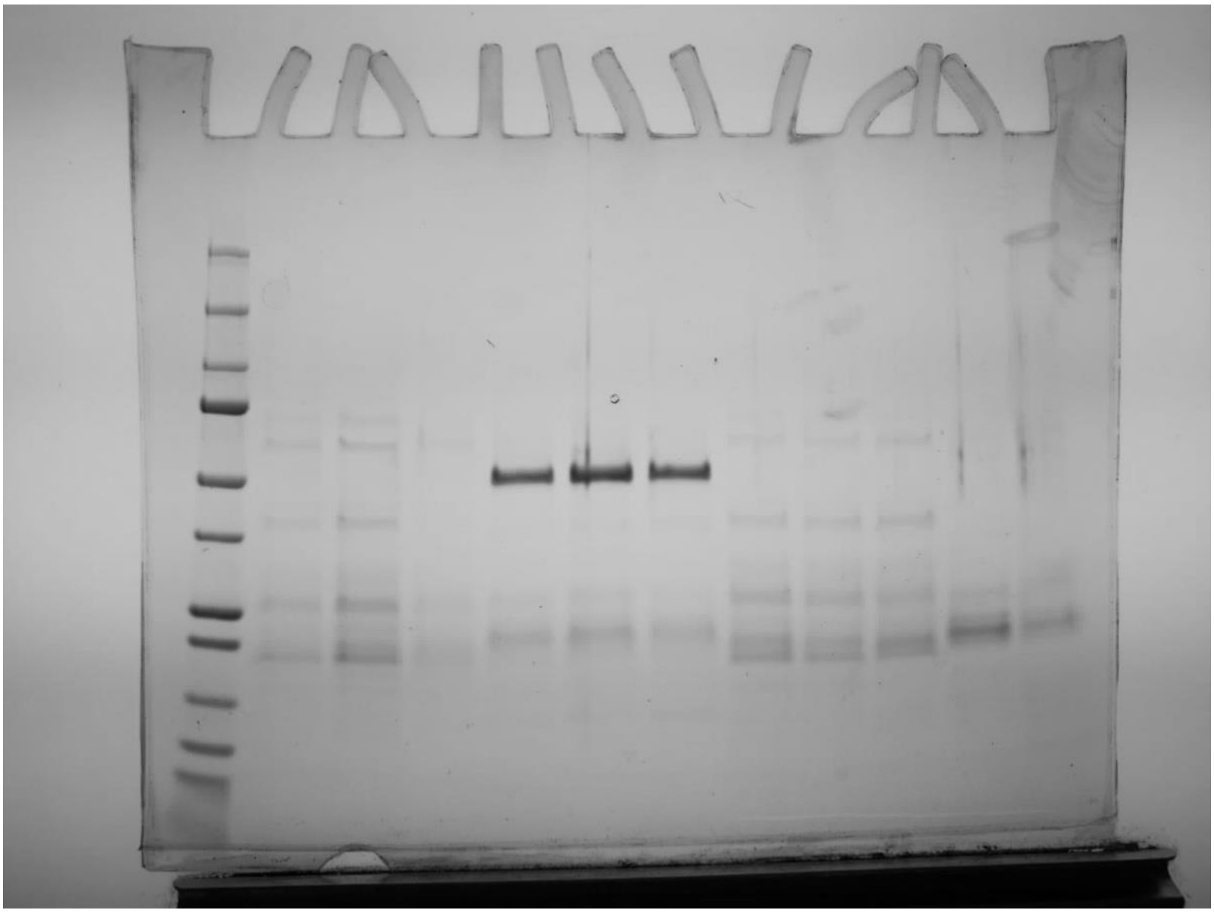

Unprocessed, uncropped version of the gel of CgnC expression trials shown in shown in Supplementary Figure 4.

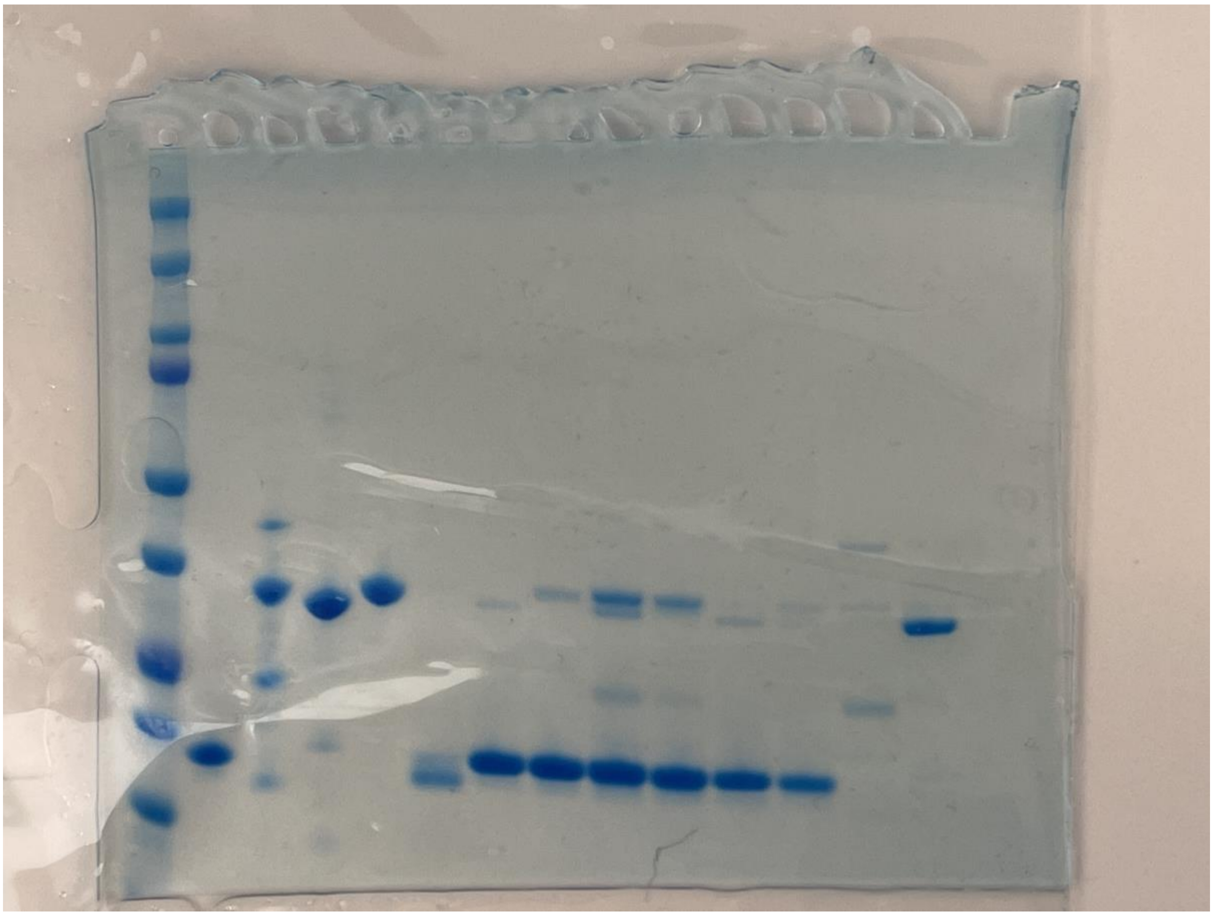

Unprocessed, uncropped gel of the results of a pull-down experiment shown in Supplementary Figure 13.
