# Supplementary material for: Unusual peptide-binding proteins guide pyrroloindoline alkaloid formation in crocagin biosynthesis
Source: Nat Chem. 2023 Mar 9;15(4):560–8. doi: 10.1038/s41557-023-01153-w (PMC10070186; doi:10.1038/s41557-023-01153-w)
Supplement: Supplementary file 2 — Reporting Summary [file 41557_2023_1153_MOESM2_ESM.pdf]

nature research

Corresponding author(s): Jesko Koehnke

Last updated by author(s): Nov 3, 2022

Reporting Summary

Nature Research wishes to improve the reproducibility of the work that we publish. This form provides structure for consistency and transparency in reporting. For further information on Nature Research policies, see our Editorial Policies and the Editorial Policy Checklist.

Statistics

For all statistical analyses, confirm that the following items are present in the figure legend, table legend, main text, or Methods section.

n/a	Confirmed	
☐	☒	The exact sample size (n) for each experimental group/condition, given as a discrete number and unit of measurement
☐	☒	A statement on whether measurements were taken from distinct samples or whether the same sample was measured repeatedly
☒	☐	The statistical test(s) used AND whether they are one- or two-sided<br>*Only common tests should be described solely by name; describe more complex techniques in the Methods section.*
☒	☐	A description of all covariates tested
☒	☐	A description of any assumptions or corrections, such as tests of normality and adjustment for multiple comparisons
☐	☒	A full description of the statistical parameters including central tendency (e.g. means) or other basic estimates (e.g. regression coefficient) AND variation (e.g. standard deviation) or associated estimates of uncertainty (e.g. confidence intervals)
☒	☐	For null hypothesis testing, the test statistic (e.g. F, t, r) with confidence intervals, effect sizes, degrees of freedom and P value noted<br>*Give P values as exact values whenever suitable.*
☒	☐	For Bayesian analysis, information on the choice of priors and Markov chain Monte Carlo settings
☒	☐	For hierarchical and complex designs, identification of the appropriate level for tests and full reporting of outcomes
☒	☐	Estimates of effect sizes (e.g. Cohen's d, Pearson's r), indicating how they were calculated

Our web collection on statistics for biologists contains articles on many of the points above.

Software and code

Policy information about availability of computer code

Data collection	NMR data were acquired and processed using Bruker TopSpin 3.5.<br>SPR data were collected using the Biacore™ X100 Plus Package Software version 2.0.2.<br>Crystal diffraction data were collected on the following plateforms: the European Synchrotron Radiation Facility (ESRF) at beamline ID-23-1, the Swiss Light Source (SLS) Beamline X10SA, the Swiss Light Source Beamline X06DA or the Deutsches Elektronen Synchrotron (DESY) at beamline P11.<br>MST data were collected using MO.Control v1.6 on a Monolith NT 115 system (Nanotemper Technologies).<br>MS data were acquired using Xcalibur, version 2.2 service pack 1.48 from Thermo Scientific OR LC software was controlled by Eksigent control software v4.3 and the acquisition software were from Analyst TF1.8.1 from AB Sciex OR Bruker Compass HyStar 5.1.8.1 (Bruker Daltonik GmbH), Bruker Compass 4.1.0.839 (Bruker Daltonik GmbH), Bruker OtofControl Version 5.2 (Build 0.8) (Bruker Daltonik GmbH) and Thermo Chromeleon 7.2.10 Build 23925 (Thermo Fisher Scientific Inc.). Protein masses were deconvoluted by using the Maximum Entropy algorithm.

Data analysis	NMR data were assigned using CCPNMR analysis assign v3.<br>SPR Data were analysed using Biacore X100 software version 2.0.2<br>Crystal data processing, structure determination, refinement and structural analysis were done using CCP4 Program suite V7.0.001, Phenix Version 1.20-4459 and COOT Version 0.9.6 under Xquartz 11 version 2.8.2. Final coordiante were checked using Molprobity website, PISA server and DALI server. All structural images portrayed were rendered in PyMOL (The PyMOL Molecular Graphics System Version 1.8.6.0, Schrödinger, LLC). Sequences comparison were done using TBBLASTN. Phylogenetic trees were constructed with RAxML[23] using the PROTGAMMAJTT model. ORF prediction was done with Geneious, Pfam families similarity with HMMer and genomic region pairs with MUSCLE<br>MST data were analysed using MO.Affinity Analysis v2.3 software for kD determination along the standart error evaluation.<br>MS data was analyzed with Xcalibur, version 2.2 service pack 1.48 from Thermo Scientific OR the display software Peakview Software 1.2.0.3, AB Sciex, OR DataAnalysis 4.4 (Bruker). Bioinformatics software that is freely available has been used as published and cited accordingly.<br>Custom scripts are available are available as part of the supplementary online files

For manuscripts utilizing custom algorithms or software that are central to the research but not yet described in published literature, software must be made available to editors and reviewers. We strongly encourage code deposition in a community repository (e.g. GitHub). See the Nature Research guidelines for submitting code & software for further information.

Data

Policy information about availability of data

All manuscripts must include a data availability statement. This statement should provide the following information, where applicable:

- Accession codes, unique identifiers, or web links for publicly available datasets
- A list of figures that have associated raw data
- A description of any restrictions on data availability

Diffraction data and refined structural models (See Supplementary Table 3) were deposited to the PDB: CgnB (6zsv), CgnD (8a2n), CgnE (6zsu), CgnL (7pd7). The very large raw mass spectrometry data files are available from the authors upon request.

Field-specific reporting

Please select the one below that is the best fit for your research. If you are not sure, read the appropriate sections before making your selection.

☒ Life sciences ☐ Behavioural & social sciences ☐ Ecological, evolutionary & environmental sciences

For a reference copy of the document with all sections, see nature.com/documents/nr-reporting-summary-flat.pdf

Life sciences study design

All studies must disclose on these points even when the disclosure is negative.

Sample size	No sample size calculation was performed.
Data exclusions	No data were excluded from the analysis.
Replication	All experiments were repeated at least three times with independent samples and the results could be fully replicated. The use of freshly prepared co-factor solutions was required for reproducibility of the biochemical experiments. The protein CgnB may aggregate if put through repeated freeze-thaw cycles.
Randomization	No experiments that were part of this study required randomization.
Blinding	No experiments that were part of this study required blinding.

Reporting for specific materials, systems and methods

We require information from authors about some types of materials, experimental systems and methods used in many studies. Here, indicate whether each material, system or method listed is relevant to your study. If you are not sure if a list item applies to your research, read the appropriate section before selecting a response.

Materials & experimental systems

n/a	Involved in the study
☒	☐ Antibodies
☒	☐ Eukaryotic cell lines
☒	☐ Palaeontology and archaeology
☒	☐ Animals and other organisms
☒	☐ Human research participants
☒	☐ Clinical data
☒	☐ Dual use research of concern

Methods

n/a	Involved in the study
☒	☐ ChIP-seq
☒	☐ Flow cytometry
☒	☐ MRI-based neuroimaging
