# Supplementary material for: Unusual peptide-binding proteins guide pyrroloindoline alkaloid formation in crocagin biosynthesis
Source: Nat Chem. 2023 Mar 9;15(4):560–8. doi: 10.1038/s41557-023-01153-w (PMC10070186; doi:10.1038/s41557-023-01153-w)
Supplement: Supplementary file 3 — Phylogenetic tree of CgnB and CgnE homologues with accession codes. [file 41557_2023_1153_MOESM3_ESM.pdf]

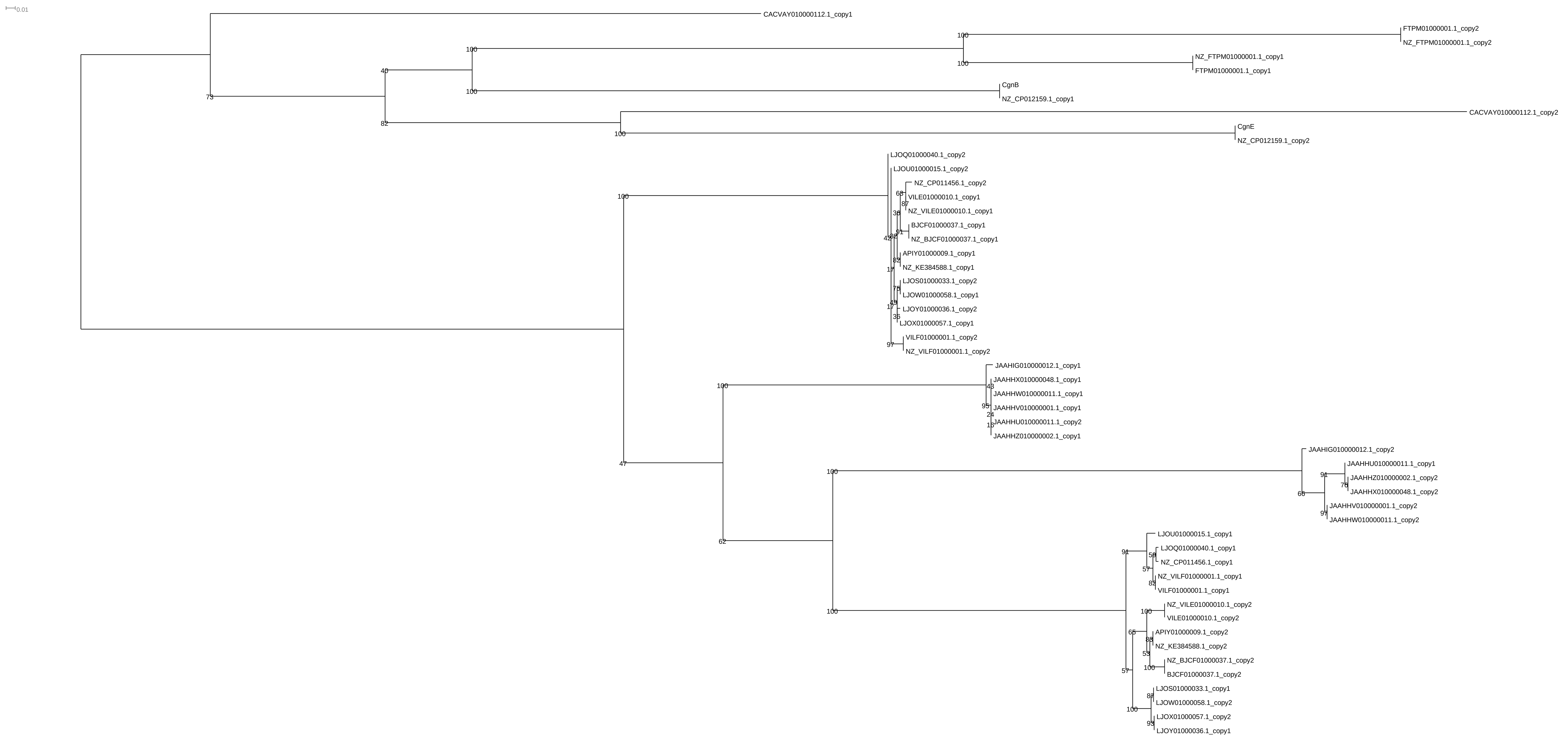
0.01
CACVAY010000112.1_copy1
FTPM01000001.1_copy2
NZ_FTPM01000001.1_copy2
NZ_FTPM01000001.1_copy1
FTPM01000001.1_copy1
CgnB
NZ_CP012159.1_copy1
CACVAY010000112.1_copy2
CgnE
NZ_CP012159.1_copy2
LJOQ01000040.1_copy2
LJOU01000015.1_copy2
NZ_CP011456.1_copy2
VILE01000010.1_copy1
NZ_VILE01000010.1_copy1
BJCF01000037.1_copy1
NZ_BJCF01000037.1_copy1
APIY01000009.1_copy1
NZ_KE384588.1_copy1
LJOS01000033.1_copy2
LJOW01000058.1_copy1
LJOY01000036.1_copy2
LJOX01000057.1_copy1
VILF01000001.1_copy2
NZ_VILF01000001.1_copy2
JAAHIG010000012.1_copy1
JAAHHX010000048.1_copy1
JAAHHW010000011.1_copy1
JAAHHV010000001.1_copy1
JAAHHU010000011.1_copy2
JAAHHZ010000002.1_copy1
JAAHIG010000012.1_copy2
JAAHHU010000011.1_copy1
JAAHHZ010000002.1_copy2
JAAHHX010000048.1_copy2
JAAHHV010000001.1_copy2
JAAHHW010000011.1_copy2
LJOU01000015.1_copy1
LJOQ01000040.1_copy1
NZ_CP011456.1_copy1
NZ_VILF01000001.1_copy1
VILF01000001.1_copy1
NZ_VILE01000010.1_copy2
VILE01000010.1_copy2
APIY01000009.1_copy2
NZ_KE384588.1_copy2
NZ_BJCF01000037.1_copy2
BJCF01000037.1_copy2
LJOS01000033.1_copy1
LJOW01000058.1_copy2
LJOX01000057.1_copy2
LJOY01000036.1_copy1
